# A quantitative reactivity scale for electrophilic fluorinating reagents†
†Electronic supplementary information (ESI) available. CCDC 1857922–1857928. For ESI and crystallographic data in CIF or other electronic format see DOI: 10.1039/c8sc03596b


**DOI:** 10.1039/c8sc03596b

**Published:** 2018-09-14

**Authors:** Neshat Rozatian, Ian W. Ashworth, Graham Sandford, David R. W. Hodgson

**Affiliations:** a Chemistry Department , Durham University , South Road , Durham , DH1 3LE , UK . Email: d.r.w.hodgson@durham.ac.uk; b AstraZeneca , Pharmaceutical Technology & Development , Macclesfield , SK10 2NA , UK

## Abstract

Electrophilic N–F fluorination agents underpin the introduction of fluorine in aliphatic systems across drug and academic research. The choice of N–F reagent is currently determined through empirical experimentation in the absence of quantitative values for electrophilicities. Here we report an experimentally-determined kinetic reactivity scale for ten N–F fluorinating reagents, including Selectfluor™, NFSI, Synfluor™ and several *N*-fluoropyridinium salts, in CH_3_CN. The reactivity scale, which covers eight orders of magnitude, employs *para*-substituted 1,3-diaryl-1,3-dicarbonyl derivatives to measure relative and absolute rate constants. The *para*-substituted 1,3-diaryl-1,3-dicarbonyl scaffold delivers a convenient, sensitive spectrophotometric reporter of reactivity that also led to the discovery of a unique form of tautomeric polymorphism.

## Abstract 2

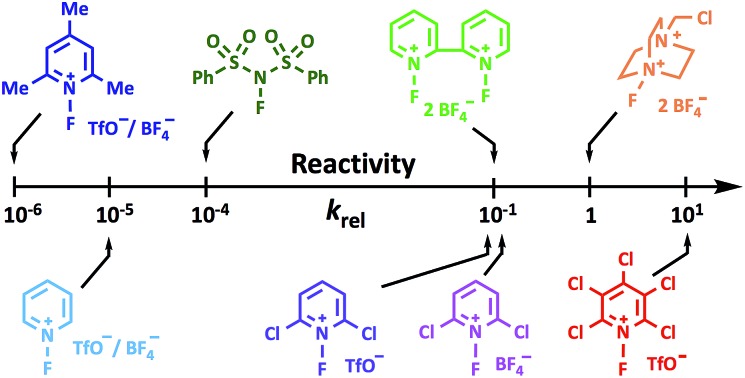
Through kinetic studies we provide a quantitative reactivity scale for ten electrophilic fluorination reagents.

## 1. Introduction

Organofluorine compounds have critically enabling roles in medicinal, agrochemical and material sciences due to the unique properties of the fluorine atom.^1^ The presence of a fluorine atom can impart beneficial changes to chemical properties and biological activities of drug molecules, such as improved metabolic stability and enhanced binding interactions.^1^ Consequently, pharmaceuticals bearing fluoro-aliphatic, -aromatic and -heterocyclic units have become widespread, *e.g.* ciprofloxacin, 5-fluorouracil, Prozac™. However, organofluorine compounds are very scarce in nature;^2^ therefore, the selective introduction of a fluorine atom is a key challenge in organic chemistry. While fluoroaromatic derivatives are synthesised industrially using anhydrous hydrogen fluoride and nucleophilic halogen exchange processes that were first reported a century ago, electrophilic strategies are less well-grounded. Electrophilic fluorination represents one of the most direct methods for the selective introduction of fluorine into organic compounds.^1^ Early work centred on reagents bearing an O–F bond (*e.g.* CF_3_OF, HOF, CsSO_4_F) or an Xe-F bond (*i.e.* XeF_2_); however, these reagents were often too reactive, unselective, difficult to prepare and not available commercially—all of which limited their adoption. Molecular fluorine (F_2_) is readily accessible, however, in order to use it safely, specialist equipment and training are required, and these factors limit its general applicability. A breakthrough came in the 1980s, with the introduction of bench-stable electrophilic fluorinating reagents containing an N–F bond.^3^ These reagents have since emerged as effective, selective and easy-to-handle sources of electrophilic fluorine, that are now commercially available and do not require specialized handling procedures.

Electrophilic N–F reagents such as Selectfluor™,^4^*N*-fluoropyridinium salts^5^–^7^ and NFSI^8^ have been widely utilised by the pharmaceutical industry in both discovery and manufacturing processes. However, the choice of reagents for the fluorination of a new scaffold at the discovery stage has generally been based on a “trial and error” approach rather than an understanding of reactivities of the electrophilic fluorinating reagent and its nucleophilic substrate. Other fundamental transformations such as nitration, alkylation, halogenation, sulfonation and Friedel–Crafts processes have been studied extensively by kinetic approaches and predictive reactivity profiles for many reagents are well established.^9^–^12^ Given the importance of fluorination reactions in the chemical, pharmaceutical and materials industries, the lack of predictive reactivity data is surprising. We now present a firm kinetic underpinning for these widely-exploited reagents.

Umemoto^13^ initiated comparative reactivity studies with his power-variable scale for *N*-fluoropyridinium salts, which centred on the electron-donating or electron-withdrawing natures of substituents on the pyridinium rings; however, the approach reflected reaction yields rather than kinetic parameters. In 1992, Lal *et al.*^14^ reported reduction potentials, *E*_p_, as measures of the relative reactivities of N–F reagents; and others have reported similar studies.^15^ Unfortunately, access to data relating to the fluorinating strength is often precluded by experimental problems. Early kinetics studies by Stavber *et al.*^16^–^19^ on the fluorination of phenols and alkenes with Selectfluor™ and Accufluor™ focused on the mechanisms of F transfer rather than reactivity comparisons. Togni and co-workers^20^ obtained the relative rate constants of seven N–F reagents for competitive halogenations of β-keto esters in the presence of a titanium catalyst. However, the *k*_rel_ values captured the whole catalytic cycle rather than individual fluorination rate constants. Most recently, a computational reactivity scale was proposed by Cheng *et al.*^21^ based on calculated fluorine plus detachment values, however, nucleophiles were not included in the models.

Our strategy focuses on utilising a common nucleophile scaffold for the correlation of the fluorinating abilities of N–F reagents. We chose 1,3-diaryl-1,3-dicarbonyls as the nucleophile basis set for our fluorination kinetics owing to the ability to enhance or subdue nucleophilicity based on the introduction of electron-donating or -withdrawing substituents. The extended conjugation within these systems offered sensitive spectrophotometric output, where keto and enol tautomers have markedly different absorption profiles. We capitalised upon the dominant enol content of the 1,3-diaryl-1,3-dicarbonyl starting materials being consumed during fluorination to afford fluoro-ketonic products.

Previous work involving the α-fluorination of carbonyl, α′-ketocarbonyl, β-dicarbonyl and related carbonyl derivatives using oxidizing fluorinating agents such as fluorine,^22^,^23^ XeF_2_,^24^,^25^ alkyl hypofluorite,^26^ perchloryl fluoride^27^ and fluoroxysulfate^28^ generally yielded mixtures of undesirable α,α-difluorinated products in addition to the α-monofluorinated products.^24^ However, N–F reagents such as *N*-fluoropyridinium salts, NFSI and Selectfluor™ have been successfully employed for the selective α-monofluorination of carbonyl derivatives.^29^,^30^ Banks *et al.* first reported the selective monofluorination of 1,3-diketones using Selectfluor™.^31^ An important field of study that has emerged is the asymmetric α-fluorination of carbonyl substrates, which has been explored with both chiral electrophilic fluorinating agents and chiral catalysts.^32^–^35^ Since the synthetic applications of N–F reagents are too numerous to cover in this paper, we refer to the excellent reviews from the recent literature to give an indication of topical fluorination reactions.^36^–^38^ Furthermore, in general, the fluorination of 1,3-dicarbonyl derivatives offers a convenient vehicle for the delivery of building blocks for the preparation of fluoro-aliphatic and -heteroaromatic systems^39^ (*e.g.* voriconazole – a billion dollar drug marketed by Pfizer^40^).

## 2. Results and discussion

### 2.1 Development of the 1,3-diaryl-1,3-dicarbonyl platform

**Fig. 1:**
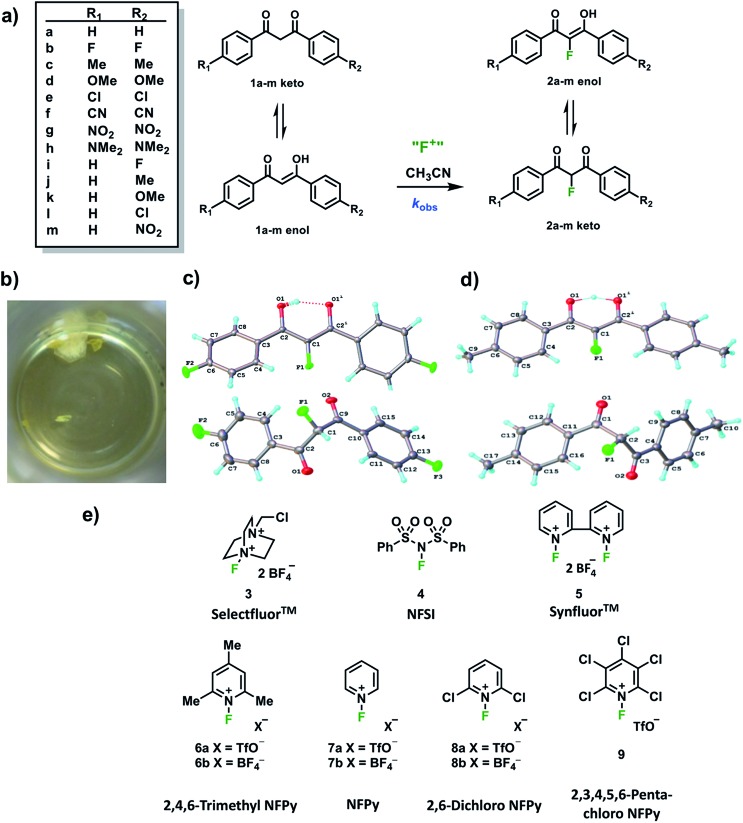
(a) Fluorination reactions of 1,3-dicarbonyls **1a–m**, monitored by UV-vis spectrophotometry. (b) Image of keto (white) and enol (yellow) crystals of **2b**, as obtained from the same solution (recrystallization from chloroform and hexane). (c) Keto (bottom) and enol (top) X-ray crystal structures for **2b**. (d) Keto (bottom) and enol (top) X-ray crystal structures for **2c** (only one position of the disordered OH hydrogen is shown). (e) Fluorinating reagents investigated in this study (NFPy = *N*-fluoropyridinium).

In order to capture the breadth of reactivities of commonly-used N–F reagents, we adopted the 1,3-diaryl-1,3-dicarbonyl derivatives **1a–m**. These systems offered the potential to tune nucleophilicity in a predictable manner through the introduction of substituents that could be amenable to Hammett correlation. The 1,3-diaryl-1,3-dicarbonyl derivatives **1a–m** (Fig. 1a) were synthesised using previously reported methods, in good yields.^41^ Compounds **1a–m** exist as mixtures of keto and enol tautomers and the ratio for each system was determined by ^1^H NMR spectroscopy in CD_3_CN (see ESI Section 2.6†). Each tautomer is easily distinguishable, with peaks at ∼4.5 ppm and ∼7 ppm corresponding to the keto and enol forms, respectively, and the OH signal of the enol form at ∼16 ppm. Compounds **1a–m** exist in ∼90% enol form in CH_3_CN, except **1h** which exists as ∼60% enol. Mono-fluorinated products **2a-f** were synthesised using Selectfluor™ (compound **3** in Fig. 1e) and the ratios of tautomers were determined by ^1^H and ^19^F NMR methods (see ESI Section 2.6†).

During the recrystallization of the fluorinated 1,3-dicarbonyls we found that the keto and enol forms of **2b** (R_1_ = R_2_ = F) and **2c** (R_1_ = R_2_ = Me) crystallized separately from the same solution. For both compounds, the keto and enol tautomers formed white and yellow crystals, respectively (Fig. 1b). On the basis of the colour differences, crystals of each tautomer were picked from the supernatant solution and analysed spectroscopically. We found that both tautomers were stable with respect to tautomerization in CDCl_3_ over the course of several days. So-called “tautomeric polymorphs” where tautomers crystallise in different crystal structures^42^ are very rare, with the CSD containing only 16 examples.^43^,^44^ We believe compounds **2b** and **2c** (Fig. 1c and d) represent the first examples of fluorinated molecules to exhibit this phenomenon.

The propensity for systems **2b** and **2c** to produce crystals of both tautomers rests on many kinetic and thermodynamic factors. In order to gauge the influence of the intrinsic stabilities of each tautomer, calculations were carried out on enol and keto monomers and dimers of **2b** using the procedures described elsewhere.^25^ The enol form is more stable as a monomer by 2.0 kJ mol^–1^ but the keto form is more stable as a dimer by 2.0 kJ mol^–1^ when the dielectric constant of *ε* = 3 is applied in the solvent model. The dielectric constant of *ε* = 3 is typical in neutral organic crystals.^25^ The very small relative energies support the possibility that crystals of both forms may be observed experimentally. The keto forms become more favourable as the solvent polarity (dielectric constant) is increased (see ESI Section 3†).

With knowledge of the differing keto–enol tautomeric equilibria of starting materials and fluorinated products in hand, we anticipated that the 1,3-diaryl-1,3-dicarbonyls should give a convenient nucleophile scaffold on which to base kinetics experiments.

### 2.2 Kinetics studies

Kinetic studies were performed on Selectfluor™, NFSI, Synfluor™, 2,6-dichloro-*N*-fluoropyridinium triflate, 2,6-dichloro-*N*-fluoropyridinium tetrafluoroborate, 2,3,4,5,6-pentachloro-*N*-fluoropyridinium triflate, *N*-fluoropyridinium triflate, *N*-fluoropyridinium tetrafluoroborate, 2,4,6-trimethyl-*N*-fluoropyridinium triflate and 2,4,6-trimethyl-*N*-fluoropyridinium tetrafluoroborate (Fig. 1e). All reagents were commercially available, except for 2,3,4,5,6-pentachloro-*N*-fluoropyridinium triflate **9**, which we synthesised from pentachloropyridine and elemental fluorine following the literature procedure.^6^

**Fig. 2:**
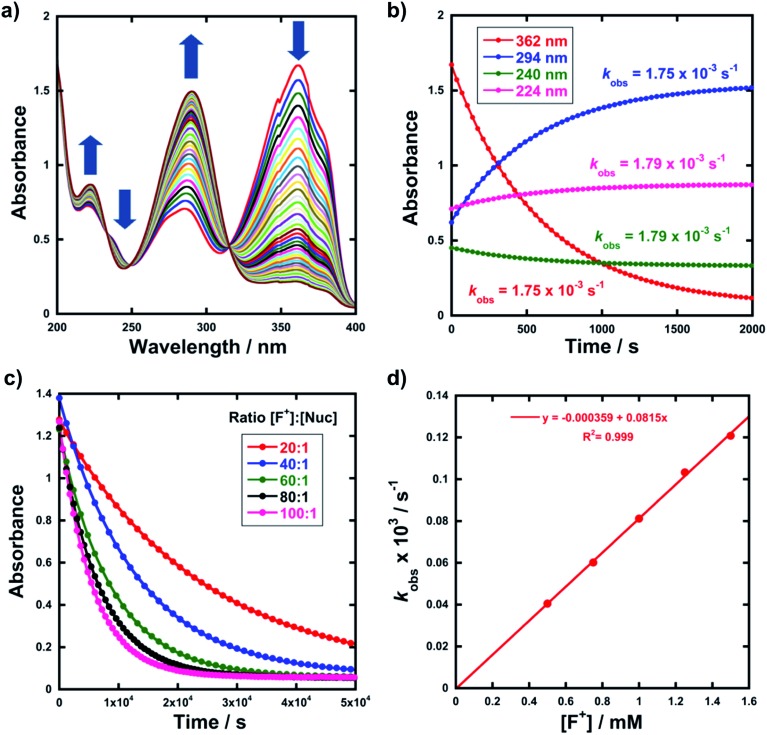
(a) UV-vis spectra during the reaction of **1d** (0.05 mM) with Selectfluor™ (in CH_3_CN at 25 °C), each spectrum acquired at 30 s intervals. The shoulders at 350 nm are artefacts that correspond to the spectrophotometer switching from UV to VIS lamps. (b) Exponential behaviour at 4 different wavelengths. (c) Representative exponential decays of absorbance with different concentrations of F^+^. (d) Representative correlation of *k*_obs_ with [F^+^].

The rates of fluorination of nucleophiles **1a–m** with electrophilic fluorinating reagents **3–9** in CH_3_CN were monitored by UV-vis spectrophotometry. A representative time-arrayed multi-wavelength study of the fluorination of **1d** by Selectfluor™ **3** (Fig. 2a) shows clean, isosbestic behaviour, suggesting that no intermediate species are built up. The nucleophiles **1a–h** show absorption bands at *λ*_max_ = 340–360 nm, corresponding to their enol forms and at *λ*_max_ = 250–270 nm, associated with a π* ← π transition of the diketone forms, as well as additional transitions due to the enol tautomer.^45^,^46^ As each fluorination reaction progresses, the absorption band at ∼250 nm increases in intensity, corresponding to the formation of the diketone form of the monofluoro-products **2a–h**, and the starting enol nucleophile signals at *λ* ∼ 350 nm decrease. Plots of absorbance changes at four *λ* values over time are shown in Fig. 2b, and fitting of these data affords identical first-order rate constants (*k*_obs_). Similar behaviours were seen across the range of 1,3-dicarbonyl derivatives and fluorinating agents.

By monitoring the decays in absorbance of the enol tautomer at *λ* ∼ 350 nm, the kinetics of fluorination reactions were conveniently monitored by UV-vis spectrophotometry. All kinetics experiments were carried out with excess electrophile in order to achieve pseudo-first order conditions. Clean exponential decays of absorbance of the UV-active nucleophile were observed in all runs (Fig. 2c), and the first-order rate constants *k*_obs_ were obtained from the fitting of plots of absorbance *versus* time. When *k*_obs_ values were plotted against Selectfluor™ concentration, a simple linear (*i.e.* first order) correlation was observed (Fig. 2d). The direct dependence upon F^+^ concentration demonstrates rate-limiting fluorination and thus the slopes of these graphs give second-order rate constants *k*_2_ [M^–1^ s^–1^] that report on both nucleophilic and electrophilic partners, according to the second-order rate eqn (1). The rate constants for the reactions of **1a–m** with each fluorinating reagent are summarized in Table 1.1
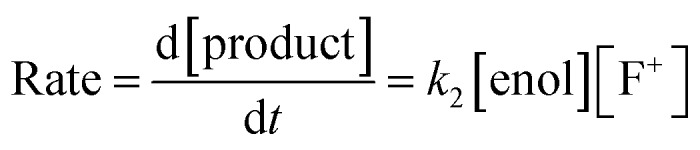



**Table 1:** Second-order rate constants (*k*_2_) for the reactions of fluorinating reagents **3–9** with nucleophiles **1a–m** in CH_3_CN, at up to four different temperatures (20 °C, 25 °C, 30 °C and 35 °C)

Nucleophile (R group)	Electrophile	*k*_2_ (20 °C)/M^–1^ s^–1^	*k*_2_ (25 °C)/M^–1^ s^–1^	*k*_2_ (30 °C)/M^–1^ s^–1^	*k*_2_ (35 °C)/M^–1^ s^–1^
**1a-enol** (R_1_ = R_2_ = H)	Selectfluor™ **3**	2.68 × 10^–2^	4.20 × 10^–2^	6.55 × 10^–2^	1.00 × 10^–1^
	NFSI **4**		9.87 × 10^–6^		
	Cl_2_-NFPy TfO^–^**8a**	5.26 × 10^–3^			
	Cl_2_-NFPy BF_4_^–^**8b**	7.98 × 10^–3^			
	Cl_5_-NFPy TfO^–^**9**	2.35	3.53		
**1b-enol** (R_1_ = R_2_ = F)	Selectfluor™ **3**	2.05 × 10^–2^	3.28 × 10^–2^	5.08 × 10^–2^	7.14 × 10^–2^
	NFSI **4**		8.14 × 10^–6^		
	Cl_2_-NFPy TfO^–^**8a**	2.23 × 10^–3^	3.35 × 10^–3^		
	Cl_2_-NFPy BF_4_^–^**8b**	8.67 × 10^–3^	1.30 × 10^–2^		
**1c-enol** (R_1_ = R_2_ = Me)	Selectfluor™ **3**	8.32 × 10^–2^	1.17 × 10^–1^	1.91 × 10^–1^	2.86 × 10^–1^
	NFSI **4**		3.08 × 10^–5^		
	Cl_2_-NFPy TfO^–^**8a**	1.68 × 10^–2^			
	Cl_2_-NFPy BF_4_^–^**8b**	2.66 × 10^–2^			
	Cl_5_-NFPy TfO^–^**9**		5.91		
**1d-enol** (R_1_ = R_2_ = OMe)	Selectfluor™ **3**	4.31 × 10^–1^	6.43 × 10^–1^	9.55 × 10^–1^	1.40
	NFSI **4**		1.38 × 10^–4^		
	Synfluor™ **5**		6.76 × 10^–2^		
	triMe-NFPy TfO^–^**6a**		1.34 × 10^–6^		
	triMe-NFPy BF_4_^–^**6b**		2.63 × 10^–6^		
	NFPy TfO^–^**7a**		6.90 × 10^–6^		
	NFPy BF_4_^–^**7b**		6.29 × 10^–6^		
	Cl_2_-NFPy TfO^–^**8a**	8.12 × 10^–2^			
	Cl_2_-NFPy BF_4_^–^**8b**	9.33 × 10^–2^			
	Cl_5_-NFPy TfO^–^**9**		2.72 × 10^1^		
**1e-enol** (R_1_ = R_2_ = Cl)	Selectfluor™ **3**	1.23 × 10^–2^	1.82 × 10^–2^	3.00 × 10^–2^	4.27 × 10^–2^
	NFSI **4**		5.75 × 10^–6^		
	Cl_2_-NFPy TfO^–^**8a**	1.96 × 10^–3^	2.94 × 10^–3^		
	Cl_2_-NFPy BF_4_^–^**8b**	3.65 × 10^–3^	5.47 × 10^–3^		
	Cl_5_-NFPy TfO^–^**9**	1.12	1.42		
**1f-enol** (R_1_ = R_2_ = CN)	Selectfluor™ **3**	1.07 × 10^–3^	1.60 × 10^–3^		
**1g-enol** (R_1_ = R_2_ = NO_2_)	Selectfluor™ **3**	5.99 × 10^–4^	8.99 × 10^–4^		
**1h-enol** (R_1_ = R_2_ = NMe_2_)	Selectfluor™ **3**	7.03 × 10^1^	1.05 × 10^2^		
	NFSI **4**		1.41 × 10^–2^		
**1i-enol** (R_1_ = H, R_2_ = F)	Selectfluor™ **3**		3.71 × 10^–2^		
**1j-enol** (R_1_ = H, R_2_ = Me)	Selectfluor™ **3**		7.70 × 10^–2^		
	NFSI **4**		1.82 × 10^–5^		
	Cl_2_-NFPy BF_4_^–^**8b**		2.39 × 10^–2^		
**1k-enol** (R_1_ = H, R_2_ = OMe)	Selectfluor™ **3**		1.89 × 10^–1^		
	NFSI **4**		4.18 × 10^–5^		
	Synfluor™ **5**		2.44 × 10^–2^		
	Cl_2_-NFPy BF_4_^–^**8b**		4.50 × 10^–2^		
**1l-enol** (R_1_ = H, R_2_ = Cl)	Selectfluor™ **3**		2.81 × 10^–2^		
**1m-enol** (R_1_ = H, R_2_ = NO_2_)	Selectfluor™ **3**		8.86 × 10^–3^		

Compounds **1a–g** and **1i–m** exist in ∼90% enol form whereas **1h** exists as ∼60% enol. We confirmed that keto–enol tautomerism was rapid under our reaction conditions by using discontinuous LCMS assays on a number of systems. We found constant keto : enol ratios throughout the reaction courses (see ESI Section 7†), where the keto and enol forms interchanged under the initially highly aqueous, acidic conditions of the LC elution gradient. Using the same LCMS approach, the fluorinated products showed only small amounts of enol form. Furthermore, we monitored a reaction mixture containing **2a-keto** and Selectfluor™ by ^19^F NMR, and found that **2a-keto** did not react to form the difluoro product over the course of 5 days. Hence, this suggests that difluorination does not occur in the UV-vis experiments (for further detailed discussion see ESI Section 8†).

We attempted to monitor the kinetics of fluorination reactions involving reagents **6a**, **6b**, **7a** and **7b** by UV-vis spectrophotometry; however, the reactions were very slow at the low concentrations required by the UV-vis method. These studies were then conducted at higher concentrations using a discontinuous NMR reaction monitoring method, where the fluorination reactions proceeded faster and at more measurable rates. Only nucleophile **1d** was used in these kinetics reactions. An initial rates method by UV-vis gave a corroborating rate constant for the reaction of **7a**, hence the UV-vis and NMR methods are in agreement (for all methods, spectra and rate constant graphs see ESI†).

### 2.3 Product analyses: reaction monitoring by NMR and LCMS

**Fig. 3:**
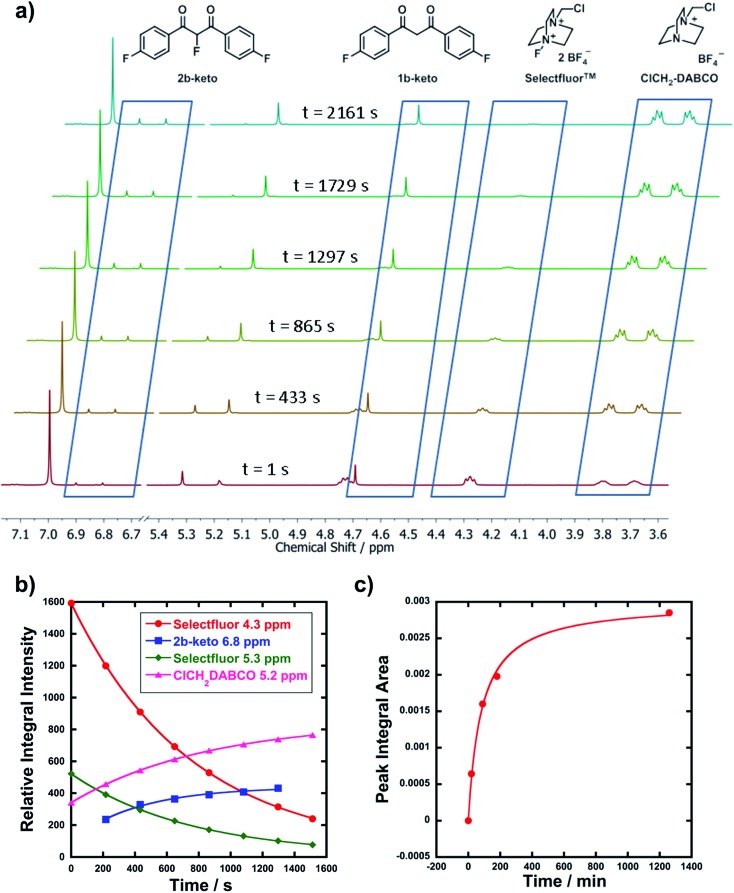
(a) Time-arrayed ^1^H NMR experiment with **1b** and Selectfluor™ **3** under pseudo-first order conditions, with a 10-fold excess of **1b**. Spectra were acquired at 3.6 min intervals and illustrative spectra from this time-course are shown above. Key signals are indicated with their associated structures. The enol form of **1b** corresponds to the peak at 7 ppm. Peaks at 5.3 and 5.2 ppm correspond to disappearance of Selectfluor™ **3** and appearance of its defluorinated product, respectively. (b) Reaction profile by ^1^H NMR (reaction of **1b** with Selectfluor™ **3**). (c) Reaction profile for LCMS analysis of the reaction between diOMe substrate **1d** and Cl_2_-NFPy BF_4_^–^**8b** under bimolecular reaction conditions ([Nuc] = [F^+^] = 3 mM).

In order to corroborate and validate our findings from UV-vis methods, NMR and LCMS experiments were employed to confirm the rates of progress of the fluorination reactions and the identities of the expected mono-fluorination products. NMR reactions were conducted in NMR tubes under pseudo-first order conditions using excess nucleophile, at 25 °C. A representative example is given in Fig. 3a, where compound **1b** (R_1_ = R_2_ = F) was reacted with Selectfluor™. Relative peak integrals from time-arrayed ^1^H NMR experiments gave exponential behaviours for the fluorination reactions (Fig. 3b), where each curve corresponds to a ^1^H signal present in Fig. 3a. The *k*_obs_ values for each curve are in the range of 1.2–1.3 × 10^–3^ s^–1^, hence they correspond to the same process. The second-order rate constant obtained was *k*_2_ = 2.2 × 10^–2^ M^–1^ s^–1^, which is in very good agreement with that obtained from UV-vis studies (3.3 × 10^–2^ M^–1^ s^–1^). The multiplets at 3.7–3.8 ppm correspond to ClCH_2_-DABCO, which is the defluorinated product of Selectfluor™. Given that the fluorination reaction was rapid, this species was already in evidence in the first NMR spectrum that was acquired.

LCMS experiments showed that keto and enol forms of both starting materials and products are clearly resolved, with their identities being confirmed through diode array analyses and the use of standards **1a–m** and **2a–f** (see ESI† for chromatogram traces). Reaction profiles for fluorination reactions were constructed *via* integration of peak areas. An example is shown in Fig. 3c, where nucleophile **1d** (R_1_ = R_2_ = OMe) was reacted with **8b** under bimolecular conditions (at 15 °C). The increase in concentration of the fluorinated product **2d** is shown, and fitting the data gave *k*_2_ = 3.4 × 10^–2^ M^–1^ s^–1^, compared to *k*_2_ = 9.3 × 10^–2^ M^–1^ s^–1^ obtained from UV-vis kinetics studies (at 20 °C). The two values are in good agreement considering the temperature differences.

### 2.4 Structure–activity correlations

**Fig. 4:**
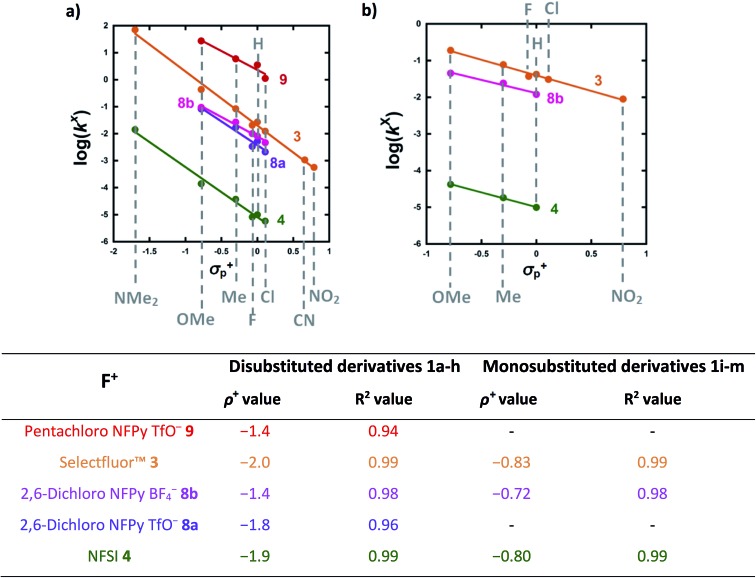
(a) Hammett correlations corresponding to fluorination of di-substituted 1,3-dicarbonyls **1a–h** by fluorinating reagents **3**, **4**, **8a**, **8b** and **9**. All rate constants used in the correlations were obtained in CH_3_CN, at 20 °C for **3**, **8a** and **8b** and at 25 °C for **4** and **9**. (b) Hammett correlations corresponding to fluorination of mono-substituted 1,3-dicarbonyls **1i–m** by fluorinating reagents **3**, **4** and **8b** in CH_3_CN at 25 °C. The corresponding *ρ*^+^ values for all Hammett plots are shown above, where *σ*^+^ values were taken from the literature.^47^

The effects of the *para*-substituents on the rates of fluorination were studied by Hammett correlation analyses of the reactions. Hammett plots were constructed for the reactions of di-substituted enols **1a–h** with fluorinating reagents **3**, **4**, **8a**, **8b** and **9** using *σ*_p_^+^ constants (Fig. 4a). The use of *σ*_p_^+^ values led to slightly better correlations than with *σ*_p_ constants in all cases (see ESI Section 5.2† where representative Hammett plots for Selectfluor™ are shown). The *ρ*^+^ values obtained for reactions involving each fluorinating reagent are between –1.4 and –2 (Fig. 4), where these negative values indicate moderate reductions in electron density on the substrates during the rate determining fluorination steps. This magnitude of electron deficit at the transition state is consistent with the S_N_2-like mechanistic behaviors that are commonly attributed to N–F reagents.

For the mono-substituted enols **1i–m**, Hammett plots were constructed using both *σ*_p_ and *σ*_p_^+^ values, with the latter giving better correlations (see ESI Section 5.2†). Hammett plots constructed for reagents **3**, **4** and **8b** are shown in Fig. 4b. The *ρ^+^* values obtained were –0.83, –0.80 and –0.72 for reactions of **3**, **4** and **8b**, respectively. The similarity in each set of *ρ^+^* values suggests that the fluorination mechanisms are analogous across the range of 1,3-dicarbonyl derivatives, which is a critical requirement for the construction of a predictive reactivity scale.

### 2.5 Reactivity scale for N–F reagents

**Fig. 5:**
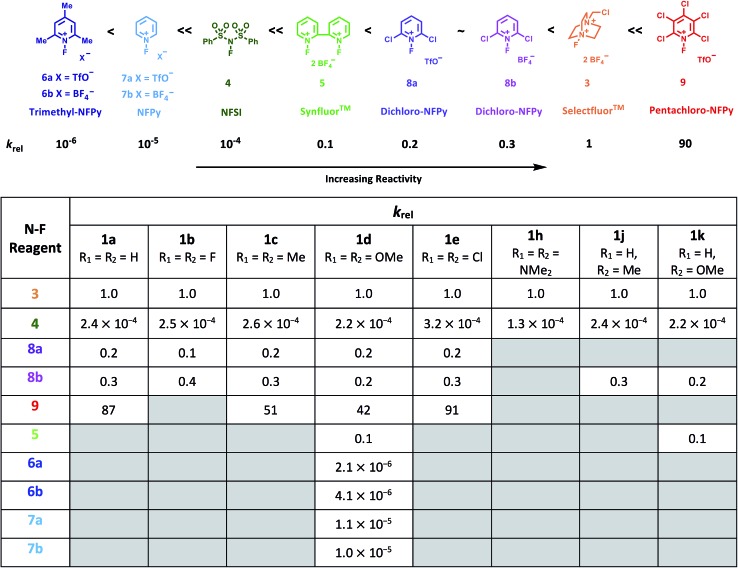
Quantitative reactivity scale of N–F fluorinating reagents. Relative rate constants were calculated based on the absolute rate constants shown in Table 1, with Selectfluor™ **3** as the reference electrophile.

Using the absolute rate constants obtained from kinetics studies *via* UV-vis reaction monitoring, relative rate constants (*k*_rel_) were calculated, using eqn (2), with Selectfluor™ as the reference electrophile (Fig. 5). Across the range of 1,3-dicarbonyl compounds **1a–m**, the *k*_rel_ values for each fluorinating reagent are in good agreement, showing the predictive potential of the scale towards nucleophiles of differing potencies.2
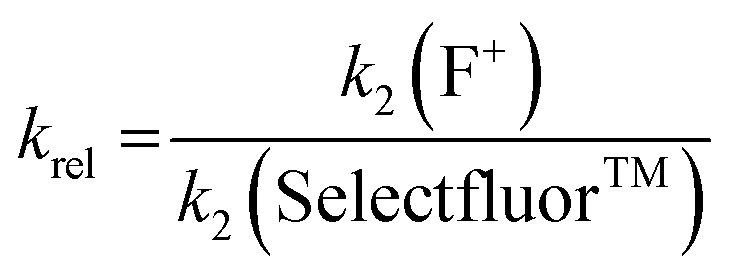



With the *k*_rel_ values in hand, we constructed a reactivity scale for fluorinating abilities of the N–F reagents (Fig. 5), in CH_3_CN. The most reactive fluorinating reagent on the scale is 2,3,4,5,6-pentachloro-*N*-fluoropyridinium triflate **9**. Selectfluor™ **3**, 2,6-dichloro-*N*-fluoropyridinium triflate **8a** and 2,6-dichloro-*N*-fluoropyridinium tetrafluoroborate **8b** have very similar reactivities, with the counter-ion having little effect on the reactivity of the *N*-fluoropyridinium salts. Synfluor™ **5** is around 10 times less reactive than Selectfluor™, although Synfluor™ is very moisture sensitive and problems arose with competing decomposition reactions when using this reagent in our studies. Therefore, rate constants with this reagent were only obtained with the most reactive nucleophiles (R_1_ = R_2_ = OMe and R_1_ = OMe, R_2_ = H), where competitive hydrolysis processes were least significant.

At the other extreme, NFSI **4** and *N*-fluoropyridinium systems **6a**, **6b**, **7a** and **7b** were 4–6 orders of magnitude less reactive than Selectfluor™ **3**. Despite the low reactivity of NFSI **4**, kinetic profiles with nucleophiles **1a–e**, **1h**, **1j** and **1k** could be obtained using UV-vis monitoring within one week, owing to its high level of solubility in CH_3_CN, which allowed large concentrations of NFSI **4** to be used with consequent enhancement of observed rates. Selectfluor™ **3**, on the other hand, shows relatively low solubility in CH_3_CN thus, although it is more reactive, reaction rates are limited because of its poorer solubility.

Although their reactivities are similar to Selectfluor™ **3**, Synfluor™ **5** and the 2,6-dichloro-*N*-fluoropyridinium salts **8a** and **8b** are very moisture sensitive. Therefore, Selectfluor™ **3** remains the most bench-stable and easy-to-handle fluorinating reagent, as water can even be used as a solvent for fluorination reactions involving this reagent.^48^ Reagents **6a**, **6b**, **7a** and **7b** are less moisture-sensitive than the dichloro-derivatives, and our NMR studies show that they remain stable in CH_3_CN solution for several weeks. Furthermore, owing to their higher levels of solubility in CH_3_CN, appreciable rates of fluorination can be achieved with these less reactive reagents through the use of higher concentrations. 2,3,4,5,6-Pentachloro-*N*-fluoropyridinium triflate **9** is highly reactive, even showing reactivity towards glass (as determined by our NMR studies – tetrafluoroborate peaks are present due to fluorination of borosilicate glass). We therefore suggest the use of plastic containers for transportation of this material. Compound **9** decomposes when heated in CH_3_CN, thus limiting the use of this reagent for reactions in this solvent at temperatures above ∼40 °C.

### 2.6 Further insight into fluorination of dicarbonyl compounds **1a–m**

**Fig. 6:**
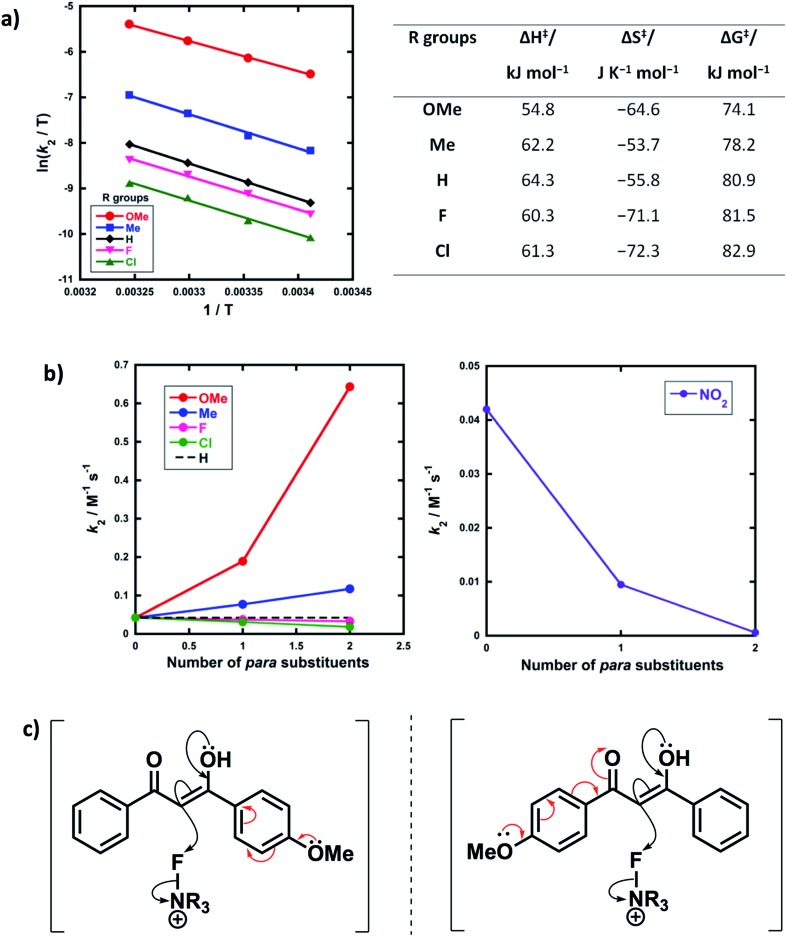
(a) Eyring plots for fluorination of 1,3-dicarbonyls **1a–e** by Selectfluor™ **3** in CH_3_CN at 20 °C, 25 °C, 30 °C and 35 °C, and associated activation parameters. (b) Effect of mono- *vs.* di-substitution on the rate of fluorination; all rate constants were obtained in CH_3_CN at 25 °C. (c) Asymmetry of the enol in the transition state.

Activation parameters (Δ*G*^‡^, Δ*H*^‡^ and Δ*S*^‡^) were obtained from kinetic data for the reactions of Selectfluor™ with **1a–e**. These experiments were performed by collecting rate constants at 4 temperatures and the resulting parameters are summarized in Fig. 6a. The Eyring plots show excellent linear correlations, with *R*^2^ > 0.99. The moderately negative values of Δ*S*^‡^ support a bimolecular, S_N_2-type mechanism for the fluorination reactions. The free energy of activation (Δ*G*^‡^) for the fluorination reactions increases from 74.1 kJ mol^–1^ to 82.9 kJ mol^–1^ as the *p*-aryl substituent of the 1,3-dicarbonyl nucleophile changes from OMe to Cl. Enthalpy of activation (Δ*H*^‡^) increases from 54.8 kJ mol^–1^ to 61.3 kJ mol^–1^ as the substituents become more electron-withdrawing. All three activation parameters are dependent on the electronic nature of the substituents, and the effect is most marked for the more electron-donating substituent OMe.

A correlation of *k*_2_*versus* the number of *para*-substituents present on each 1,3-dicarbonyl was constructed using the rate constants obtained from kinetics studies with Selectfluor™ and compounds **1a–e**, **1g** and **1i–m** (Fig. 6b). As expected, nucleophiles with two electron-donating substituents (*e.g.* R_1_ = R_2_ = OMe) show an increase in reactivity towards fluorination compared with the mono-substituted derivatives. Conversely, two electron-withdrawing groups at the *para* positions cause a greater decrease in nucleophilicity at C-2 than only one EWG, and hence the rate of fluorination is slower with the di-substituted compounds. The *para*-substituents are thus working in synergy, rather than showing “push–pull” effects.

Furthermore, nucleophiles displaying substituents that have mostly inductive electron-withdrawing or electron-donating effects show a linear trend in the graphs of *k*_2_*versus* number of *para*-substituents. On the other hand, the OMe substituents have a non-linear correlation of rate constants *versus* number of substituents, and cause a strong increase in reactivity compared to **1a** due to the strong electron-donating nature of each OMe group. A similar non-linear correlation was obtained with *para*-nitro groups (Fig. 6b). The non-additive effects between mono- and di-substituted substrates are consistent with the asymmetric nature of enol systems preventing identical conjugation effects by the substituents in the di-substituted systems (Fig. 6c).

## 3. Conclusion

We have provided a quantitative reactivity scale that spans eight orders of magnitude, for ten commonly-exploited fluorination reagents. The reactivity of each fluorinating reagent was assessed by directly monitoring the kinetics of fluorination reactions with a family of 1,3-diaryl-1,3-dicarbonyl nucleophiles that mirrors the application of the reagents in C–F bond formation. The reactivities of the homologous nucleophiles span 5 orders of magnitude and allowed reactivity determinations to be performed in a genuinely comparative manner using a convenient spectrophotometric readout. Similar Hammett parameters across the range of fluorination reagents revealed the mechanisms of fluorination to be similar.

## 4. Methods

The ESI† contains details of kinetic experiments, product analyses and spectra of all characterized compounds.

## Contributions

N. R. designed and executed all experimental work and prepared the manuscript. I. W. A. provided guidance on the interpretation of the results. G. S. contributed to the conception of the project, provided supervision to N. R. and edited the manuscript. D. R. W. H. conceived the project, supervised N. R. and edited the manuscript.

## Conflicts of interest

The authors declare no competing financial interest.

## Supplementary Material

Supplementary information

Crystal structure data
