# Supplementary material for: A quantitative reactivity scale for electrophilic fluorinating reagents
Source: Chem Sci. 2018 Sep 14;9(46):8692–702. doi: 10.1039/c8sc03596b (PMC6263395; doi:10.1039/c8sc03596b)
Supplement: Supplementary file 1 [file SC-009-C8SC03596B-s001.pdf]

Electronic Supplementary Material (ESI) for Chemical Science.
This journal is © The Royal Society of Chemistry 2018

A Quantitative Reactivity Scale for Electrophilic Fluorinating Reagents

Neshat Rozatian[a], Ian W. Ashworth[b], Graham Sandford[a], David R. W. Hodgson[a]*

a) **Chemistry Department, Durham University, South Road, Durham, UK, DH1 3LE**
b) **AstraZeneca, Pharmaceutical Technology & Development, Macclesfield, UK, SK10 2NA**

SUPPORTING INFORMATION

Table of Contents

1. General Instrumentation and Materials 5

2. Experimental 5

2.1 Preparation of 1,3-diaryl-1,3-propanediones 5

2.1.1 1,3-bis(4'-cyanophenyl)-1,3-propanedione **1f** 5

2.1.2 1,3-bis(4'-nitrophenyl)-1,3-propanedione **1g** 6

2.1.3 1,3-bis[4'-(dimethylamino)phenyl]-1,3-propanedione **1h** 7

2.2 Preparation of 2-fluoro-1,3-diaryl-1,3-propanediones 7

2.2.1 Synthesis of 2-fluoro-1,3-diphenyl-1,3-propanedione **2a** 7

2.2.2 Synthesis of 2-fluoro-1,3-bis(4’-fluorophenyl)-1,3-propanedione **2b** 8

2.2.3 Synthesis of 2-fluoro-1,3-bis(4’-methylphenyl)-1,3-propanedione **2c** 9

2.2.4 Synthesis of 2-fluoro-1,3-bis(4’-methoxyphenyl)-1,3-propanedione **2d** 10

2.2.5 Synthesis of 2-fluoro-1,3-bis(4’-chlorophenyl)-1,3-propanedione **2e** 11

2.2.6 Synthesis of 2-fluoro-1,3-bis(4-cyanophenyl)-1,3-propanedione **2f** 12

2.3 Synthesis of 2,3,4,5,6-pentachloro-*N*-fluoropyridinium trifluoromethanesulfonate 12

2.4 NMR Spectra for Novel Compounds 13

2.4.1 2-fluoro-1,3-bis(4’-fluorophenyl)-1,3-propanedione **2b** 13

2.4.2 2-fluoro-1,3-bis(4’-methylphenyl)-1,3-propanedione **2c** 19

2.4.3 2-fluoro-1,3-bis(4’-chlorophenyl)-1,3-propanedione **2e** 22

2.4.4 2-fluoro-1,3-bis(4-cyanophenyl)-1,3-propanedione **2f** 28

2.5 Distinguishing Fluoro-Keto and Fluoro-Enol Forms by NMR 29

2.6 Keto:Enol Ratios for Compounds **1a-m** and **2a-e** 30

3. Computational Methods 31

4. X-ray Crystallography 32

5. Kinetics Conducted by UV-Vis Spectrophotometry 34

5.1 Methods 34

5.2 Hammett Correlations for Selectfluor™ 35

5.3 Determination of Activation Parameters for the Reaction of Selectfluor™ with Nucleophiles **1a-e** 36

5.4 Kinetics Reactions Involving Selectfluor™ (**3**) at 4 Different Temperatures 37

5.4.1 Nucleophile **1a** 37

5.4.2 Nucleophile **1b** 39

5.4.3 Nucleophile **1c** 42

5.4.4 Nucleophile **1d** 44

5.4.5 Nucleophile **1e** ... 47
5.4.6 Nucleophile **1f** ... 49
5.4.7 Nucleophile **1g** ... 50
5.4.8 Nucleophile **1h** ... 50
5.4.9 Nucleophile **1i** ... 51
5.4.10 Nucleophile **1j** ... 52
5.4.11 Nucleophile **1k** ... 52
5.4.12 Nucleophile **1l** ... 53
5.4.13 Nucleophile **1m** ... 54
5.5 Kinetics Reactions Involving NFSI (**4**) ... 55
5.5.1 Nucleophile **1a** ... 55
5.5.2 Nucleophile **1b** ... 56
5.5.3 Nucleophile **1c** ... 56
5.5.4 Nucleophile **1d** ... 57
5.5.5 Nucleophile **1e** ... 58
5.5.6 Nucleophile **1h** ... 58
5.5.7 Nucleophile **1j** ... 59
5.5.8 Nucleophile **1k** ... 60
5.6 Kinetics Reactions Involving Synfluor™ (**5**) ... 61
5.6.1 Nucleophile **1d** ... 61
5.6.2 Nucleophile **1k** ... 62
5.7 Kinetics Reactions Involving 2,6-dichloro-*N*-fluoropyridinium triflate (**8a**) ... 63
5.7.1 Nucleophile **1a** ... 63
5.7.2 Nucleophile **1b** ... 63
5.7.3 Nucleophile **1c** ... 64
5.7.4 Nucleophile **1d** ... 65
5.7.5 Nucleophile **1e** ... 66
5.8 Kinetics Reactions Involving 2,6-dichloro-*N*-fluoropyridinium tetrafluoroborate (**8b**) ... 67
5.8.1 Nucleophile **1a** ... 67
5.8.2 Nucleophile **1b** ... 67
5.8.3 Nucleophile **1c** ... 68
5.8.4 Nucleophile **1d** ... 69
5.8.5 Nucleophile **1e** ... 70
5.8.6 Nucleophile **1j** ... 70

5.8.7 Nucleophile **1k** 71
5.9 Kinetics Reactions Involving 2,3,4,5,6-pentachloro-N-fluoropyridinium triflate (**9**) 72
5.9.1 Nucleophile **1a** 72
5.9.2 Nucleophile **1c** 73
5.9.3 Nucleophile **1d** 74
5.9.4 Nucleophile **1e** 75
6. Kinetics Studies Conducted by $^{1}H/^{19}F$ NMR 76
6.1 Fluorination of Nucleophile **1d** by *N*-fluoropyridinium triflate (**7a**) 76
6.2 Fluorination of Nucleophile **1d** by *N*-fluoropyridinium tetrafluoroborate (**7b**) 77
6.3 Fluorination of Nucleophile **1d** by 2,4,6-trimethyl-*N*-fluoropyridinium triflate (**6a**) 79
6.4 Fluorination of Nucleophile **1d** by 2,4,6-trimethyl-*N*-fluoropyridinium BF_4^- (**6b**) 80
7. Reactions Monitored by LCMS 82
7.1 Distinguishing Keto and Enol Tautomers 82
7.2 Fluorination of **1a** by Selectfluor™ 83
7.3 Fluorination of **1d** by 2,6-dichloro-NFPy tetrafluoroborate (**8b**) 84
8. Kinetics of Fluorination of **2a** 85
9. References 87

1. General Instrumentation and Materials

^{1}H NMR (400 MHz), ^{13}C NMR (101 MHz) and ^{19}F NMR (376 MHz) were measured on a Bruker-Avance 400 MHz spectrometer. LC-MS data were obtained using a triple quadrupole mass spectrometer equipped with an Acquity UPLC (Waters Ltd, UK), EH C18 column (1.7µm, 2.1mm x 50mm) and a photodiode array detector. Conditions for LC resolution were as follows: buffer A = water, 0.1% formic acid; buffer B = MeCN. Elution conditions: Flow rate = 0.6 mL/min; 0-0.2 min isocratic 95% A, 5% B; 0.2-4 min linear gradient to 5% A, 95% B; 4-4.5 min isocratic 5% A, 95% B; 4.5-5 min linear gradient to 95% A, 5% B. Chemicals were purchased from Fluorochem, TCI or Sigma Aldrich and, unless otherwise stated, used without purification. NMR solvents were purchased from Cambridge Isotopes Inc., supplied by Goss Scientific and Sigma-Aldrich. These chemicals were used without further purification and stored under appropriate conditions, as detailed in the manufacturer's instructions. Organic solvents were used without further purification. Selectfluor™ and NFSI were purchased from Fluorochem; fluorinating reagent **8a** was purchased from Sigma-Aldrich; fluorinating reagents **5-7** and **8b** were purchased from TCI and used without further purification.

2. Experimental

Compound **1a** was bought from Sigma Aldrich and was recrystallized (hexane) and dried under vacuum before use in kinetic measurements. The 1,3-diaryl-1,3-propanediones **1b-m** were synthesised according to literature procedures[1] and recrystallized from hexane/ethyl acetate before use in kinetics experiments.

2.1 Preparation of 1,3-diaryl-1,3-propanediones

2.1.1 1,3-bis(4'-cyanophenyl)-1,3-propanedione **1f**

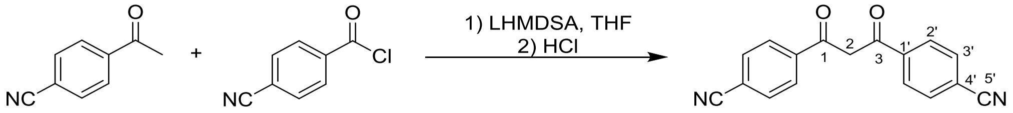

A mixture comprising of 4'-acetylbenzonitrile (0.50g, 3.44 mmol) and $LiN(SiMe_3)_2$ (1 M in THF, 6.89 mmol, 6.9 mL) in anhydrous THF (7 mL) were stirred at −78 °C for 30 min. 4'-Cyanobenzoyl chloride (0.57 g, 3.44 mmol) was added and the mixture was stirred at RT overnight. Upon quenching the reaction with 37% HCl (1 mL), the product was precipitated as a yellow solid and was recovered by filtration and washed with water. The filtrate was extracted with ethyl acetate (3 x 10 mL), and the combined organic phases were washed with sodium bicarbonate (10 mL) and water (10 mL). Drying ($MgSO_4$) and evaporation of solvent *in vacuo* yielded further product. Both batches of solid were recrystallized from EtOH to give pure 1,3-bis(4'-cyanophenyl)-1,3-propanedione (0.51 g, 54%) as a

yellow solid. **IR** (ATR) $υ_{max}$/ cm^{-1} 3070 (C-H arom), 2230 (CN), 1582 (conj. enol), 1522, 1447, 1290, 1222, 1020, 860, 784, 694, 542. **^{1}H NMR** (400 MHz, $CDCl_3$) δ = 8.08 (4H, d, $^{3}J_{HH}$ = 8.2 Hz, 2'-*H*), 7.81 (4H, d, $^{3}J_{HH}$ = 8.3 Hz, 3'-*H*), 6.86 (1H, s, 2-*H* of enol). **^{13}C NMR** (101 MHz, $CDCl_3$) δ = 184.2 (C-1, C-3), 138.8 (C-1'), 132.6 (C-3'), 127.8 (C-2'), 117.9 (C-5'), 116.1 (C-4'), 94.5 (C-2). **ESI-MS** (ES^-, R_t 2.892 min) m/z 273.091 $[M-H]^-$. **M.p.** (EtOH) = 220 °C (lit.[2] m.p. 220– 222 °C).

These assignments are in agreement with the literature.[2]

2.1.2 1,3-bis(4'-nitrophenyl)-1,3-propanedione **1g**

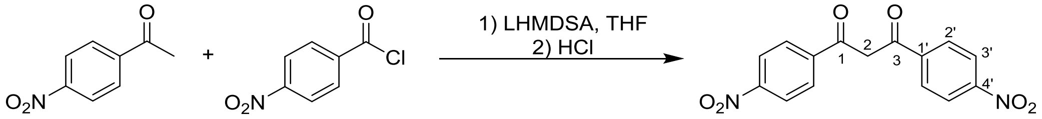

1g

A mixture comprising of 4'-nitroacetophenone (0.52g, 3.16 mmol) and $LiN(SiMe_3)_2$ (1 M in THF, 6.06 mmol, 6.1 mL) in anhydrous THF (7 mL) was stirred at −78 °C for 30 min. 4'-Nitrobenzoyl chloride (0.59 g, 3.16 mmol) was added and the mixture was stirred at RT overnight. The crude product was quenched with 37% HCl (1 mL), and the product precipitated as a brown solid which was filtered and washed with water. The filtrate was extracted with ethyl acetate (3 x 10 mL), washed with sodium bicarbonate (10 mL) and water (10 mL) and dried ($MgSO_4$). The solvent was evaporated to yield the crude as a red solid. Both batches of crude product were recrystallized from ethyl acetate to give pure 1,3-bis(4'-nitrophenyl)-1,3-propanedione (0.76 g, 77%) as a brown solid (98% enol tautomer in $CDCl_3$). **IR** (ATR) $υ_{max}$/ cm^{-1} 3126 (C-H arom), 1580 (conj. enol), 1510 (s, NO_2), 1340 (s, NO_2), 1320, 1224, 1109, 1048, 1010, 857, 786, 744, 709; **^{1}H NMR** (400 MHz,$CDCl_3$) δ = 15.99 (1H, s, RC=C-O*H*), 8.40-8.32 (4H, m, 2'-*H*), 8.20-8.13 (4H, dq, J_{HH} = 9.2, 2.2 Hz, 3'-*H*), 6.93 (1H, s, 2-*H* of enol). **^{13}C NMR** (101 MHz, $CDCl_3$) δ = 184.3 (C1, C3), 150.6 (C-1'), 140.7 (C-3'), 128.7 (C-2'), 124.4 (C-4'), 95.4 (C-2). **ESI-MS** (ES^-, R_t 3.159) m/z 313.273 $[M-H]^-$. **HRMS (ES^-/Q-TOF) m/z:** $[M-H]^-$ Calcd for $C_{15}H_9N_2O_6$ 313.0469; found 313.0453. **M.p.** (EtOAc) = 237-238 °C (lit.[3] m.p. 238 – 243 °C).

These assignments are in agreement with the literature.[4]

2.1.3 1,3-bis[4'-(dimethylamino)phenyl]-1,3-propanedione **1h**

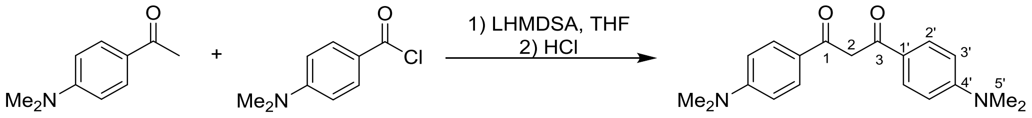

1h

A mixture comprising of 1-[4’-(dimethylamino)phenyl]ethanone (1.0g, 6.13 mmol) and $LiN(SiMe_3)_2$ (1 M in THF, 12.3 mmol, 12 mL) in anhydrous THF (14 mL) were stirred at −78 °C for 30 min. 4’-(Dimethylamino)benzoyl chloride (1.13 g, 6.13 mmol) was added and the mixture was stirred at RT overnight. The crude product was quenched with KH_2PO_4 (3 g), extracted with ethyl acetate (3 x 30 mL), washed with sodium bicarbonate (30 mL) and water (30 mL) and dried ($MgSO_4$). The solvent was evaporated to yield the crude as a yellow solid. This was recrystallized from ethanol to yield 1,3-bis[4'-(dimethylamino)phenyl]-1,3-propanedione (1.18 g, 62%) as brown crystals (60% enol in CD_3CN). **IR** (ATR) υ_{max}/ cm^{-1} 2894, 1602, 1561, 1476, 1432 1370, 1235, 1164, 1063, 948, 923, 783, 710. **^{1}H NMR** (400 MHz, CD_3CN): enol signals: δ = 7.92 (4H, d, $^3J_{HH}$ 9.2, 2’-*H*), 6.80 (1H, s, 2-*H*), 6.77 (4H, d, $^3J_{HH}$ 9.1, 3’-*H*), 3.05 (12H, s, 5’-*H*); keto signals: δ = 7.83 (4H, d, $^3J_{HH}$ 9.2, 2’-H), 6.71 (4H, d, $^3J_{HH}$ 9.1, 3’-*H*), 4.42 (2H, s, 2-*H*), 3.03 (12H, s, 5’-*H*). **ESI-MS** (ES^+, R_t 3.244) m/z 311.753 $[M+H]^+$ enol, (ES^+, R_t 2.510) m/z 312.589 $[M+2H]^+$ keto.

These assignments are in agreement with the literature.[5]

2.2 Preparation of 2-fluoro-1,3-diaryl-1,3-propanediones

Fluorinated compounds **2a-f** were obtained as a mixture of keto and enol forms, as identified from NMR of the crude and pure products. Purification by recrystallization allowed the isolation of a single or both tautomers in most cases. Where possible, the NMR spectra for each tautomer have been assigned separately.

2.2.1 Synthesis of 2-fluoro-1,3-diphenyl-1,3-propanedione **2a**

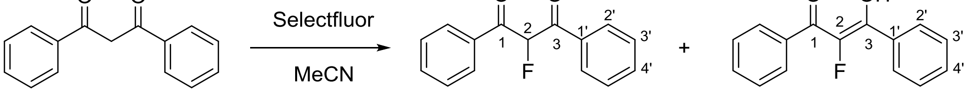

1a **2a-keto** **2a-enol**

1,3-diphenyl-1,3-propanedione (227 mg, 1 mmol) was dissolved in dry MeCN (10 mL) and Selectfluor™ (354 mg, 1 mmol) was added. The reaction mixture was stirred at room temperature for 2.5 h and monitored by TLC. The solvent was evaporated *in vacuo*, and the white residue was dissolved in CH_2Cl_2 (20 mL) and washed with water (5 × 20 mL). The organic phase was separated, dried ($MgSO_4$), solvent evaporated *in vacuo* and the crude product was obtained as an off-white

solid. Recrystallization was done from hexane to obtain white crystals of 2-fluoro-1,3-diphenyl-1,3-propanedione (170 mg, 70%) as a 98:2 mixture of keto:enol forms in $CDCl_3$. **IR** (ATR) υ_{max}/ cm^{-1} 3071 (C-H arom), 1698 (C=O), 1670 (C=O), 1593 (arom C=C), 1576, 1448, 1284, 1229, 1181, 1098, 967, 868, 778, 708. **^{1}H NMR** (400 MHz, $CDCl_3$) δ = 10.18 (1H, s, RC=C-O*H*), 8.15-8.05 (4H, dq, J_{HH} = 7.7, 1.2 Hz, 2'-*H* keto), 7.65-7.59 (2H, m, 4'-*H* keto), 7.52-7.46 (4H, m, 3'-*H* keto), 6.54 (1H, d, $^2J_{HF}$ = 49.2 Hz, 2-*H* keto). **^{13}C NMR** (101 MHz, $CDCl_3$) δ = 191.5 (d, $^2J_{CF}$ = 20.2 Hz, C1, C3), 134.9 (s, C3'), 133.9 (d, $^3J_{CF}$ = 1.9 Hz, C1'), 130.2 (d, $^4J_{CF}$ = 3.5 Hz, C2'), 129.1 (s, C4'), 97.0 (d, $^1J_{CF}$ = 199.0 Hz, C2). **^{19}F NMR** (376 MHz, $CDCl_3$) δ = −186.9 (d, J_{FH} = 49.8 Hz, keto), −166.5 (s, enol). **ESI-MS** (ES^+, R_t 3.988) m/z 243 $[M+H]^+$ enol; (ES^+, R_t 3.390) m/z 265 $[M+Na]^+$ keto. **M.p.** (hexane) = 65-66 °C (lit.[6] m.p. 66.5-66.7 °C).

These assignments are in agreement with the literature.[7]

Crystal structure: keto tautomer

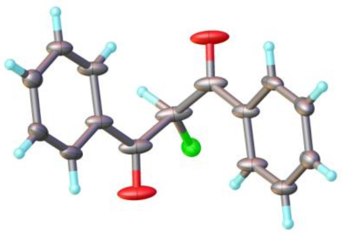

2.2.2 Synthesis of 2-fluoro-1,3-bis(4'-fluorophenyl)-1,3-propanedione **2b**

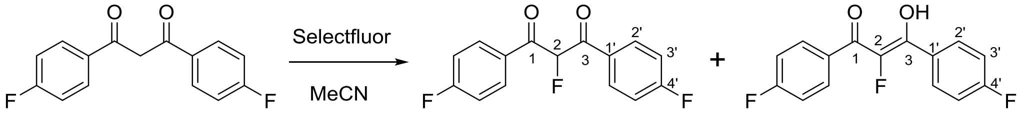

1b **2b-keto** **2b-enol**

1,3-bis(4'-fluorophenyl)-1,3-propanedione (131 mg, 0.50 mmol) was dissolved in MeCN (10 mL) and Selectfluor™ (178 mg, 0.50 mmol) was added. The reaction mixture was stirred at room temperature for 96 h. The solvent was evaporated *in vacuo*, and the yellow residue was dissolved in CH_2Cl_2 (20 mL) and washed with water (5 × 20 mL). The organic phase was separated, dried ($MgSO_4$), solvent evaporated *in vacuo* and the crude product was obtained as a yellow solid. The crude material was purified by recrystallization from a mixture of chloroform and hexane, to yield the pure product as a yellow solid (100 mg, 71%, 98:2 keto:enol). An additional recrystallization step was carried out (chloroform/hexane), and on visual inspection, two different types of crystals were seen to be present. Crystals of **2b-keto** were white whereas crystals of **2b-enol** were yellow. Individual crystals were analysed by NMR spectroscopy and X-ray crystallography to confirm the constitutions of these tautomers.

Keto tautomer: **^{1}H NMR** (400 MHz, $CDCl_3$) δ = 8.19-8.12 (4H, m, 2'-*H*), 7.20-7.12 (4H, m, 3'-*H*), 6.48 (1H, d, $^2J_{HF}$ = 49.3, 2-*H*). **^{13}C NMR** (101 MHz, $CDCl_3$) δ = 189.8 (d, $^2J_{CF}$ = 20.4 Hz, C1, C3), 166.9 (d, $^1J_{CF}$ = 258.2 Hz, C4'), 133.2 (dd, J_{CF} = 9.7, 3.77 Hz, C_{arom}), 130.1 (t, J_{CF} = 2.59 Hz, C_{arom}), 116.5 (d, J_{CF} = 22.0 Hz, C_{arom}), 97.0 (d, $^1J_{CF}$ = 199.7 Hz, C2). **^{19}F NMR** (376 MHz, $CDCl_3$) δ = −186.1 (d, $^1J_{FH}$ = 49.9 Hz, C2-*F*), −101.5 (s, 2 x C4'-*F*).

Enol tautomer: **^{1}H NMR** (400 MHz, $CDCl_3$) δ = 14.86 (1H, d, ^4J = 3.3 Hz, RC=C-O*H*), 8.11-8.03 (4H, m, 2'-*H*), 7.23-7.15 (4H, m, 3'-*H*). **^{13}C NMR** (101 MHz, $CDCl_3$) δ = 174.9 (d, $^2J_{CF}$ = 21.1 Hz, C1), 165.5 (dd, $^1J_{CF}$ = 254.9, 1.7 Hz, C2), 144.3 (d, $^1J_{CF}$ = 235.8 Hz, C4'), 132.0 (t, $^3J_{CF}$ = 9.13 Hz, C1'), 129.9 (d, $^3J_{CF}$ = 9.13 Hz, C2'), 116.1 (d, $^2J_{CF}$ = 21.7 Hz, C3'). **^{19}F NMR** (376 MHz, $CDCl_3$) δ = −168.9 (s, C2-*F*), −105.4 (s, 2x C4'-*F*).

Crystal structures: keto and enol tautomers

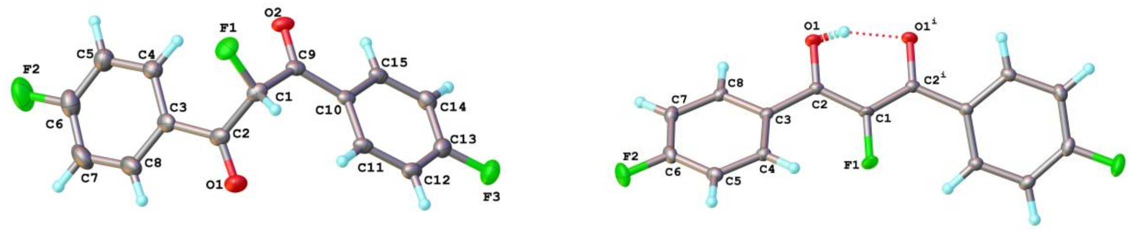

2.2.3 Synthesis of 2-fluoro-1,3-bis(4'-methylphenyl)-1,3-propanedione **2c**

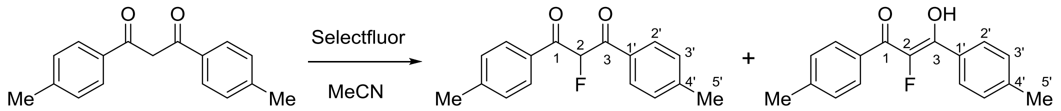

1c **2c (keto)** **2c (enol)**

1,3-bis(4'-methylphenyl)-1,3-propanedione (141 mg, 0.56 mmol) was dissolved in MeCN (15 mL) and Selectfluor™ (198 mg, 0.56 mmol) was added. The reaction mixture was stirred at room temperature for 44 h. The solvent was evaporated *in vacuo* to give a residue which was dissolved in CH_2Cl_2 (20 mL) and washed with water (5 × 20 mL). The organic phase was separated and dried ($MgSO_4$). The solvent was evaporated *in vacuo* to give the crude product as a yellow solid. The crude material was purified by recrystallization from a mixture of chloroform and hexane, to yield the pure product as a yellow solid (80 mg, 53%). Low yield was obtained as the product is very soluble in chloroform, so some product is lost during recrystallization, but can be recovered from the supernatant. The pure compound contained a 97:3 mixture of keto:enol forms in $CDCl_3$. An additional recrystallization step was performed (chloroform/hexane) to obtain crystals of **2c-keto** (white) and **2c-enol** (yellow). **IR** (ATR) υ_{max}/ cm^{-1} 1697 (C=O), 1667 (C=O), 1604 (arom C=C), 1288, 1246, 1233, 1186 (C-F), 1091, 1038, 960, 877, 825, 752, 686. **^{1}H NMR** (400 MHz, $CDCl_3$) δ = 15.02 (1H, br, s, RC=C-O*H*), 8.02-7.96 (4H, m, 2'-*H* keto), 7.30-7.23 (4H, m, 3'-*H* keto), 6.49 (1H, d, $^2J_{HF}$ = 49.3 Hz, 2-*H* keto), 2.43 (6H, s, 5'-*H*

enol), 2.40 (6H, s, 5′-*H* keto). **^{13}C NMR** (101 MHz, $CDCl_3$) δ = 190.9 (d, $^{2}J_{CF}$ = 20.1 Hz, C-1, C-3 keto), 175.7 (d, $^{2}J_{CF}$ = 20.9 Hz, C-1 enol), 145.8 (s, C-4′ keto), 143.2 (s, C-4′ enol), 131.3 (d, J_{CF} = 2.2 Hz, C_{arom} keto), 130.1 (d, J_{CF} = 3.5 Hz, C_{arom} keto), 129.6 (s, C-3′ keto), 96.9 (d, $^{1}J_{CF}$ = 198.5 Hz, C-2 keto), 21.9 (s, C-5′ keto), 21.8 (s, C-5′ enol). **^{19}F NMR** (376 MHz, $CDCl_3$) δ = −186.7 (d, $^{2}J_{FH}$ = 49.8 Hz, keto), −168.8 (s, enol). **ESI-MS**: m/z 271 (61%) $[M+H]^+$, 288 (100%) $[M+NH_4]^+$. **M.p.** (chloroform/hexane) = 88 – 89 °C.

Crystal structures: keto and enol tautomers

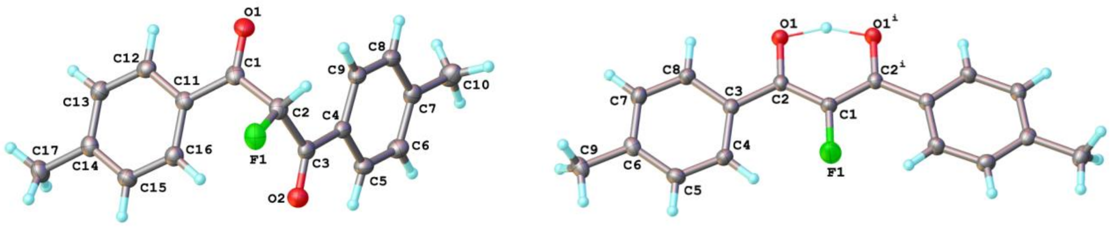

2.2.4 Synthesis of 2-fluoro-1,3-bis(4′-methoxyphenyl)-1,3-propanedione **2d**

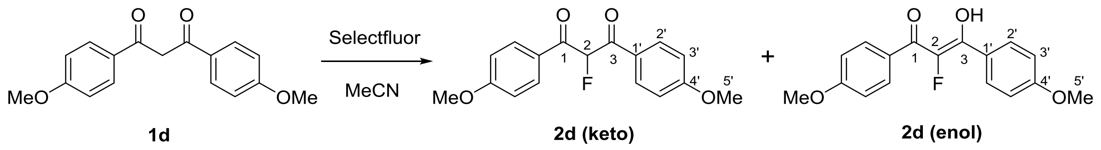

1,3-bis(4′-methoxyphenyl)-1,3-propanedione (129 mg, 0.45 mmol) was dissolved in MeCN (10 mL) and Selectfluor™ (160 mg, 0.45 mmol) was added. The reaction mixture was stirred at room temperature for 2 h. The solvent was evaporated *in vacuo* and the white residue was dissolved in CH_2Cl_2 (20 mL) and washed with water (5 x 20 mL). The organic phase was separated, dried ($MgSO_4$) and the solvent evaporated *in vacuo.* The crude product was obtained as a yellow oil which partially solidified under vacuum. Recrystallization was done from a mixture of chloroform and hexane to yield the pure product as a yellow solid (88 g, 64%), as a 98:2 mixture of keto:enol forms. Crystals of **2d-enol** were obtained via vapour diffusion crystallization. **IR** (ATR) υ_{max}/ cm^{-1} 3014 (C-H arom), 2844 (C-H methyl), 1683 (C=O), 1659 (C=O), 1598 (arom C=C), 1571, 1510, 1312, 1252, 1170 (C-F), 1081, 1012, 961, 828. **^{1}H NMR** (400 MHz, $CDCl_3$) δ = 15.29 (1H, br, s, RC=C-O*H*), 8.14-8.07 (4H, m, 2′-*H*, keto), 8.07-8.02 (4H, m, 2′-*H*, enol), 7.01-6.95 (4H, m, 3′-*H*, enol), 6.96-6.90 (4H, m, 3′-*H*, keto), 6.45 (1H, d, $^{2}J_{HF}$ = 49.4 Hz, 2-*H*, keto), 3.86 (6H, s, 5′-*H*). **^{13}C NMR** (101 MHz, $CDCl_3$) δ = 189.9 (d, $^{2}J_{CF}$ = 20.0 Hz, C-1, C-3), 164.9 (s, C-4′), 132.7 (d, $^{3}J_{CF}$ = 3.6 Hz, C-1′), 126.9 (d, $^{4}J_{CF}$ = 2.1 Hz, C-2′), 114.3 (s, C-3′), 97.3 (d, $^{1}J_{CF}$ = 198.2 Hz, C-2), 55.9 (s, C-5′). **^{19}F NMR** (376 MHz, $CDCl_3$) δ = −186.0 (d, $^{2}J_{FH}$ = 50.0 Hz, keto), −169.4 (s, enol). **ESI-MS** (ES^+, R_t 2.638) m/z 303 (100%) $[M+H]^+$. **M.p.** (chloroform/hexane) = 65 °C. Lit: from DCM/hexane 87-88 °C.

These assignments are in agreement with the literature.[8]

Crystal structure: enol tautomer

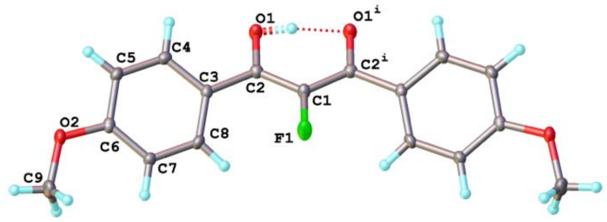

2.2.5 Synthesis of 2-fluoro-1,3-bis(4'-chlorophenyl)-1,3-propanedione **2e**

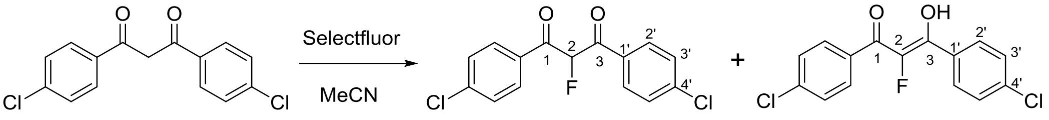

1,3-bis(4'-chlorophenyl)-1,3-propanedione (150 mg, 0.51 mmol) was dissolved in MeCN (20 mL) and Selectfluor™ (181 mg, 0.51 mmol) was added. The reaction mixture was stirred at room temperature for 96 h. The solvent was evaporated *in vacuo* and CH_2Cl_2 (20 mL) was added to the yellow residue, which was then washed with water (5 × 20 mL). The organic phase was separated, dried ($MgSO_4$) and the solvent evaporated *in vacuo* to obtain the crude product as a yellow solid. Initial purification was carried out by recrystallization (chloroform/hexane) to obtain the pure product as yellow crystals (140 mg, 88%, keto:enol 82:18 in $CDCl_3$). Recrystallization was performed via vapour diffusion to obtain **2e-enol** as yellow crystals. **IR** (ATR) υ_{max}/ cm^{-1} 2560 (C-H arom), 1679 (C=O), 1588 (C=C arom), 1425, 1400, 1295, 1178, 1090, 99, 838, 746. **M.p.** (chloroform/hexane) = 122 – 123 °C. **Elem. Anal.** Calcd for $C_{15}H_9Cl_2FO_2$: C, 57.9; H, 2.92; N, 0. Found: C, 57.62; H, 2.50; N, -0.09.

Keto tautomer: **^{1}H NMR** (400 MHz, $CDCl_3$) δ = 8.06-8.02 (4H, m, 2'-*H*), 7.48-7.44 (4H, m, 3'-*H*), 6.47 (1H, d, $^{2}J_{HF}$ = 49.2, 2-*H*). **^{13}C NMR** (101 MHz, $CDCl_3$) δ = 190.2 (d, $^{2}J_{CF}$ = 20.5 Hz, C1, C3), 141.8 (s, C_{arom}), 132.0 (d, $^{4}J_{CF}$ = 2.3 Hz, C2'), 131.6 (d, $^{3}J_{CF}$ = 3.8 Hz, C1'), 129.6 (s, C_{arom}), 96.9 (d, $^{1}J_{CF}$ = 200.1 Hz, C2). **^{19}F NMR** (376 MHz, $CDCl_3$) δ = −186.6 (d, $^{2}J_{FH}$ = 49.8 Hz).

Enol tautomer: **^{1}H NMR** (400 MHz, $CDCl_3$) δ = 14.74 (1H, br, s, RC=C-O*H*), 8.00-7.95 (4H, m, 2'-*H*), 7.50-7.45 (4H, m, 3'-*H*). **^{13}C NMR** (101 MHz, $CDCl_3$) δ = 175.0 (d, $^{2}J_{CF}$ = 21.2 Hz, C1), 144.5 (d, $^{1}J_{CF}$ = 236.9 Hz, C2), 139.3 (d, J_{CF} = 1.8 Hz, C_{arom}), 131.7 (d, J_{CF} = 5.0 Hz, C_{arom}), 130.8 (d, J_{CF} = 9.0 Hz, C_{arom}), 129.2 (s, C4'). **^{19}F NMR** (376 MHz, $CDCl_3$) δ = −168.0 (s).

Crystal structure: enol tautomer

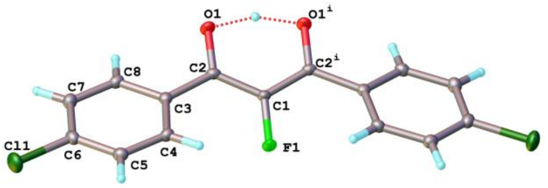

2.2.6 Synthesis of 2-fluoro-1,3-bis(4-cyanophenyl)-1,3-propanedione **2f**

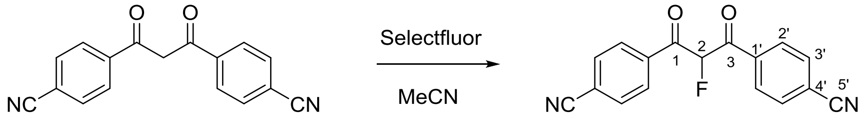

1f **2f**

1,3-bis(4’-cyanophenyl)-1,3-propanedione (71 mg, 0.26 mmol) was dissolved in MeCN (15 mL) and Selectfluor™ (92 mg, 0.26 mmol) was added. The reaction mixture was stirred at room temperature for 1 week. The solvent was evaporated *in vacuo*, and the yellow residue was dissolved in CH_2Cl_2 (20 mL) and washed with water (5 × 20 mL). The organic phase was separated, dried ($MgSO_4$) and the solvent was evaporated *in vacuo.* The crude product was obtained as a yellow solid, which was purified by recrystallisation (chloroform/hexane) to give the pure product as an 84:16 mixture of enol:keto forms (47 mg, 62 %). **^{1}H NMR** (400 MHz, $CDCl_3$) δ = 15.02 (1H, br, s, RC=C-O*H*), 8.07-8.01 (4H, m, 2’-*H*), 7.82-7.76 (4H, m, 3’-*H*), 6.55 (1H, d, $^2J_{FH}$ = 48.9, 2-*H* keto). **^{19}F NMR** (376 MHz, $CDCl_3$) δ = −187.2 (d, $^2J_{FH}$ = 48.8 Hz, keto), −166.9 (s, enol). **ESI-MS** (ES^-, R_t 2.457 min) m/z 291.232 $[M-H]^-$.

2.3 Synthesis of 2,3,4,5,6-pentachloro-*N*-fluoropyridinium trifluoromethanesulfonate (9)

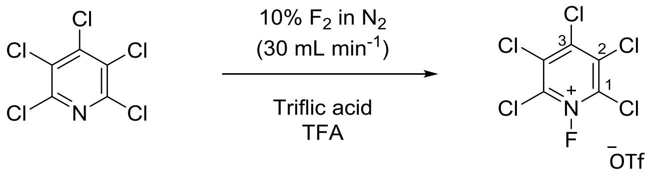

Pentachloropyridine (2g, 7.9 mmol) was dissolved in trifluoroacetic acid (70 mL), and triflic acid (1.0 mL, 11.3 mmol) was added to the solution. The mixture was purged with nitrogen for 15 min and maintained at 10 °C using a temperature-controlled bath. 10% F_2 in N_2 was passed through the mixture at 30 mL min^{-1} for 4.2 hours. The mixture was purged with nitrogen for 15 min, then the trifluoroacetic acid was evaporated *in vacuo*, and the oily residue treated with ethyl acetate. The resulting white solid was filtered, washed with ethyl acetate, and dried *in vacuo*. Recrystallisation from cold MeCN gave the product as a white crystalline solid (1.2 g, 36%). **^{19}F NMR** (376 MHz, CD_3CN) δ = +47.0 (s, NF), −79.4 (s, TfO^-).

2.4 NMR Spectra for Novel Compounds

2.4.1 2-fluoro-1,3-bis(4’-fluorophenyl)-1,3-propanedione **2b**

Keto tautomer:

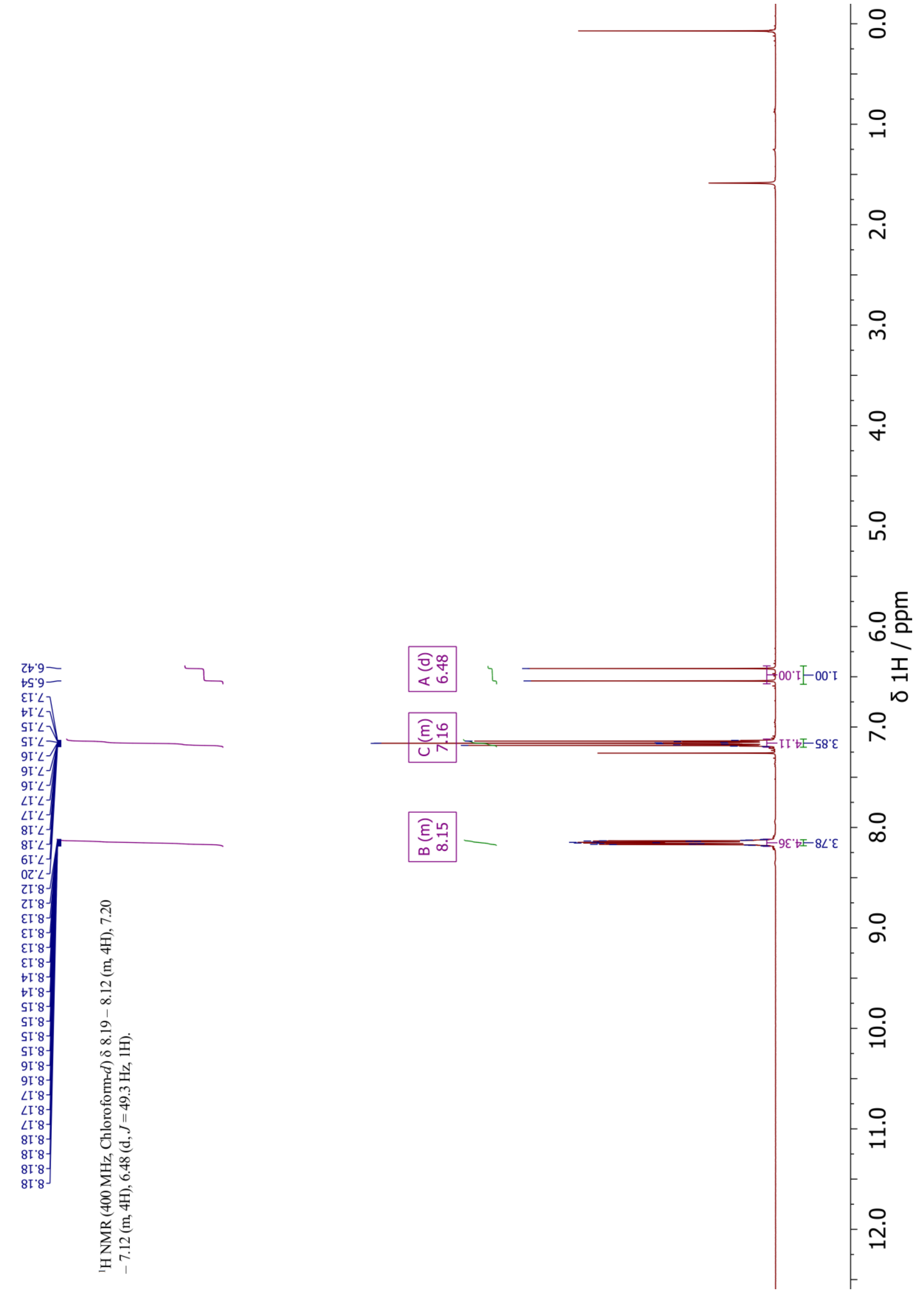

^{13}C NMR (101 MHz, Chloroform-*d*) δ 189.84 (d, *J* = 20.4 Hz), 166.93 (d, *J* = 258.2 Hz), 133.16 (dd, *J* = 9.8, 3.8 Hz), 130.14 (t, *J* = 2.6 Hz), 116.49 (d, *J* = 22.0 Hz), 96.99 (d, *J* = 199.7 Hz).

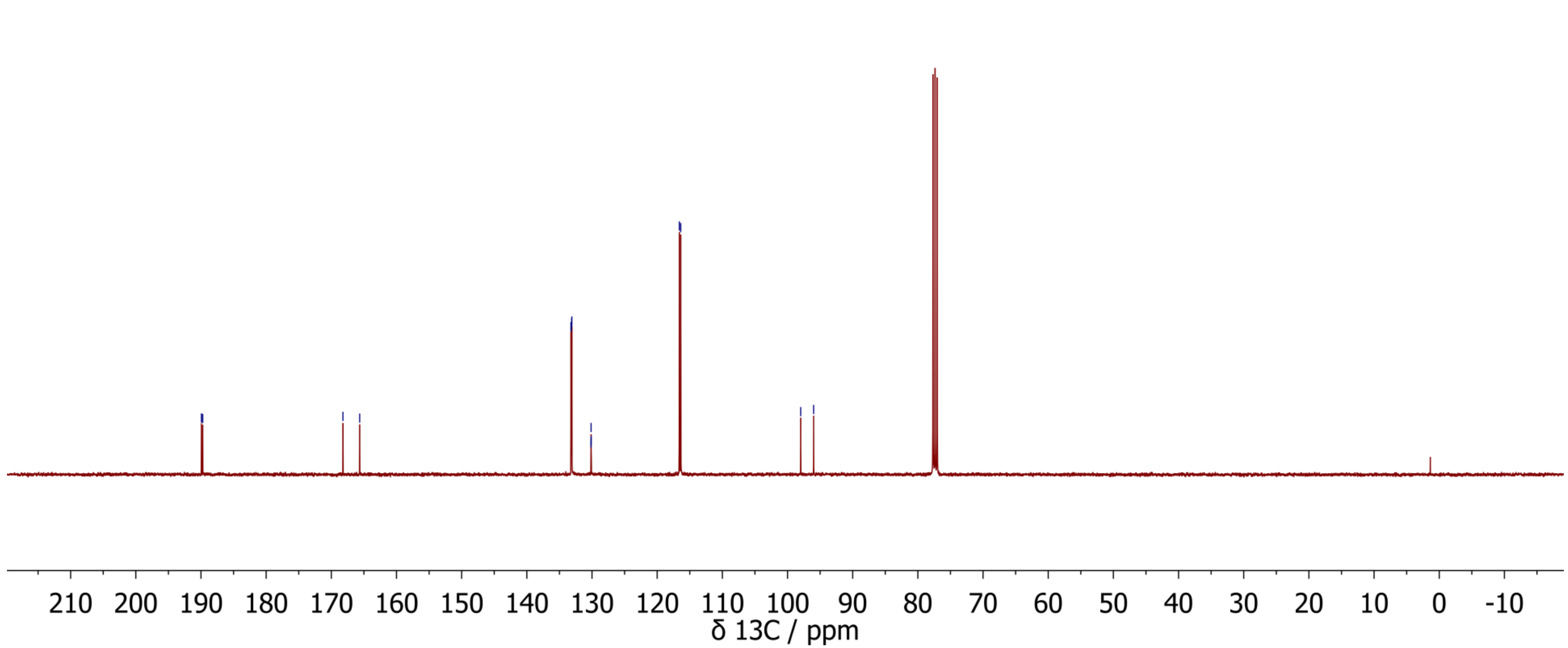

^{19}F NMR (376 MHz, Chloroform-*d*) δ -100.98 – -102.21 (m), -186.11 (d, *J* = 49.9 Hz).

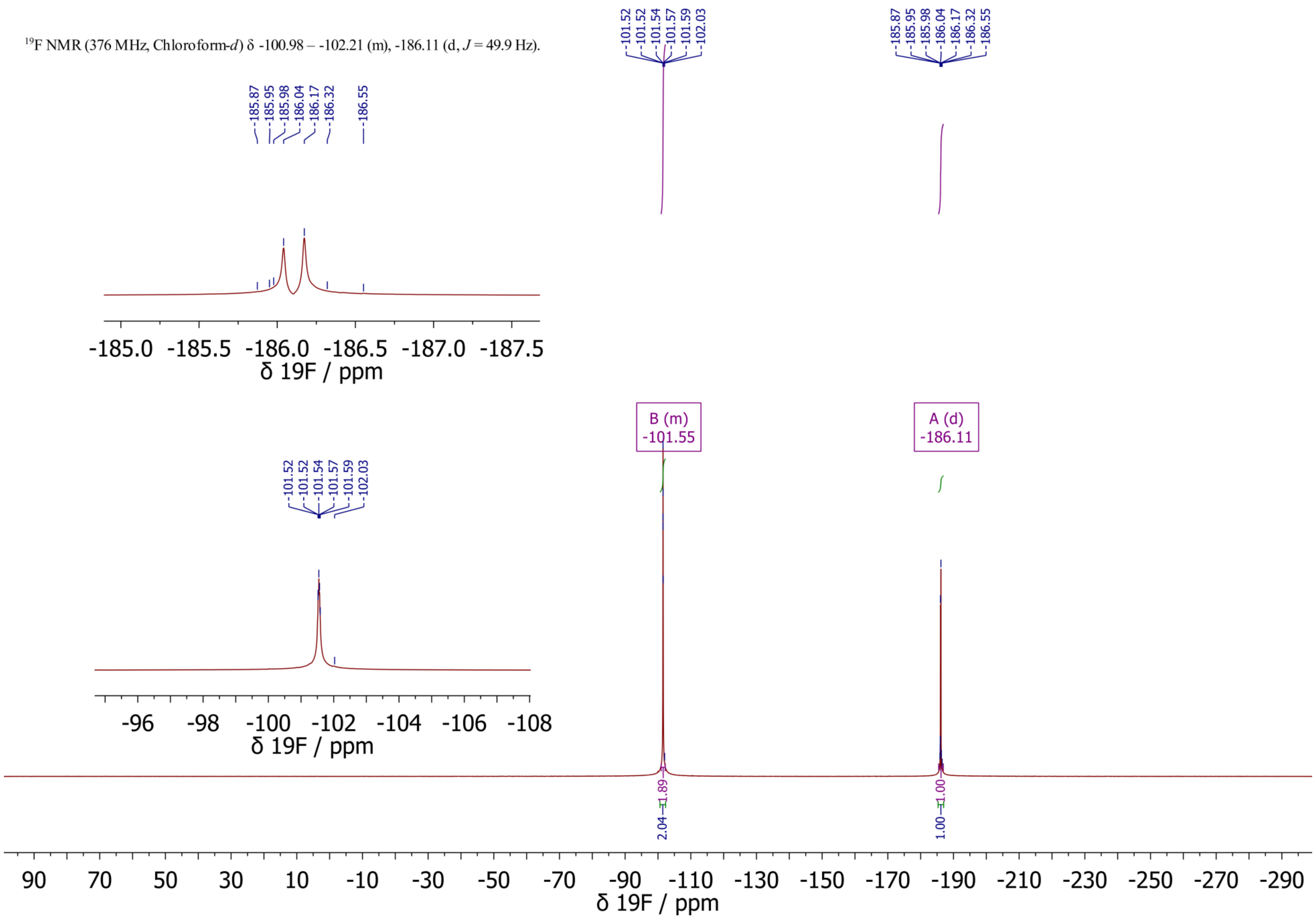

Enol tautomer:

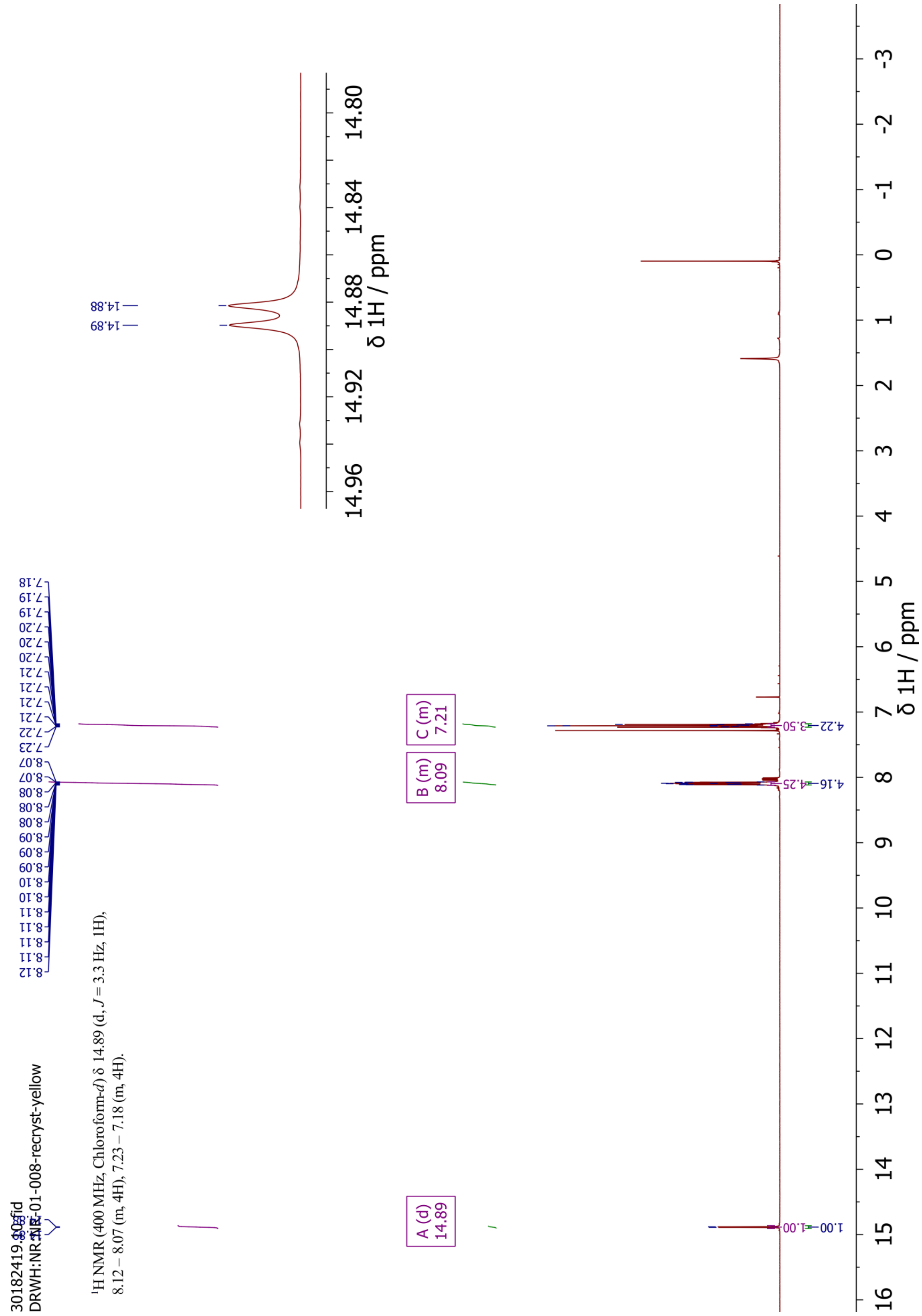

^{13}C NMR (101 MHz, Chloroform-*d*) δ 174.93 (d, J = 21.1 Hz), 165.53 (dd, J = 254.9, 1.7 Hz), 144.30 (d, J = 235.7 Hz), 132.03 (t, J = 9.1 Hz), 129.93 (d, J = 9.1 Hz), 129.73 – 129.54 (m), 116.12 (d, J = 21.7 Hz).

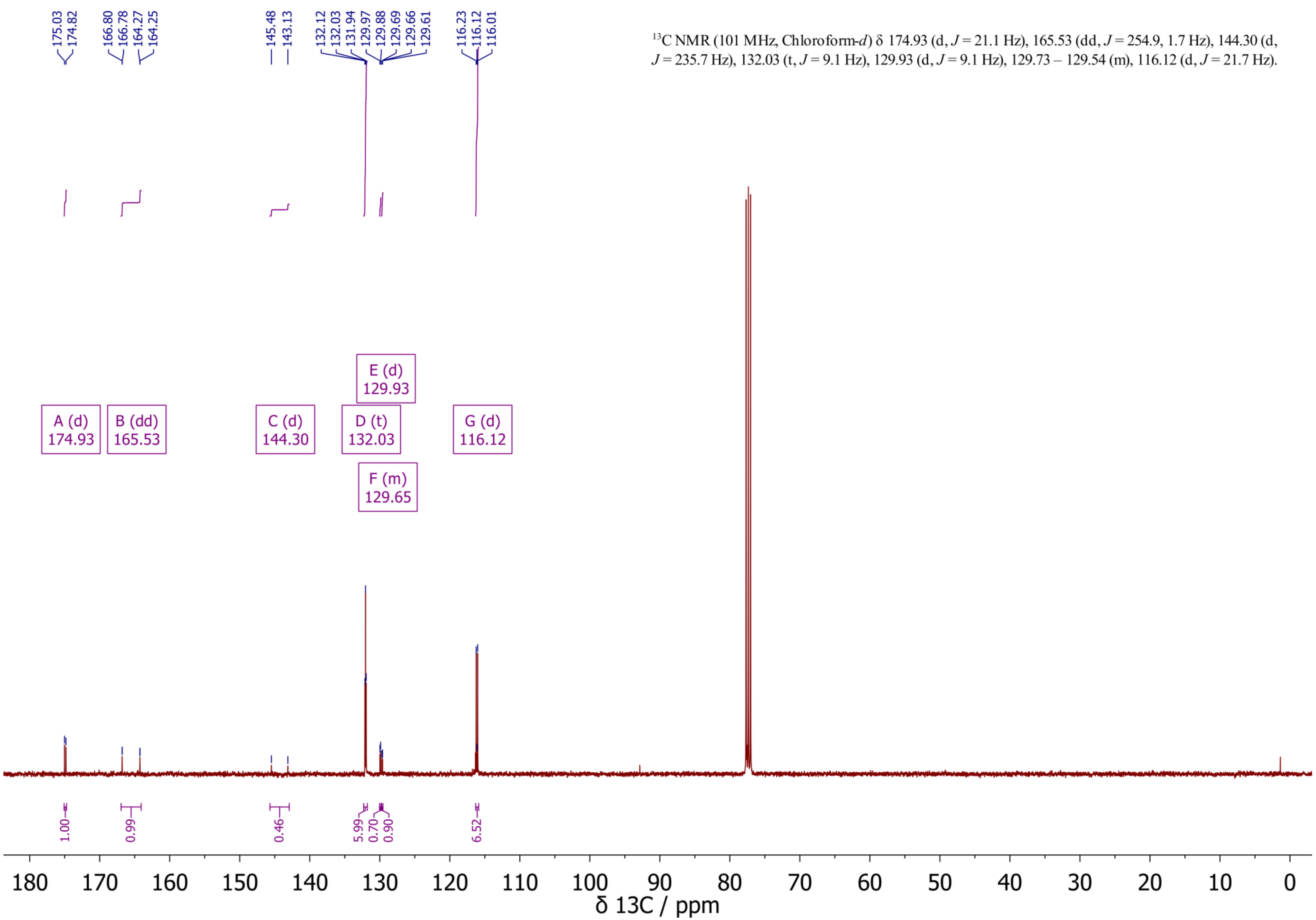

^{19}F NMR (376 MHz, Chloroform-*d*) δ -104.81 – -105.90 (m), -168.91.

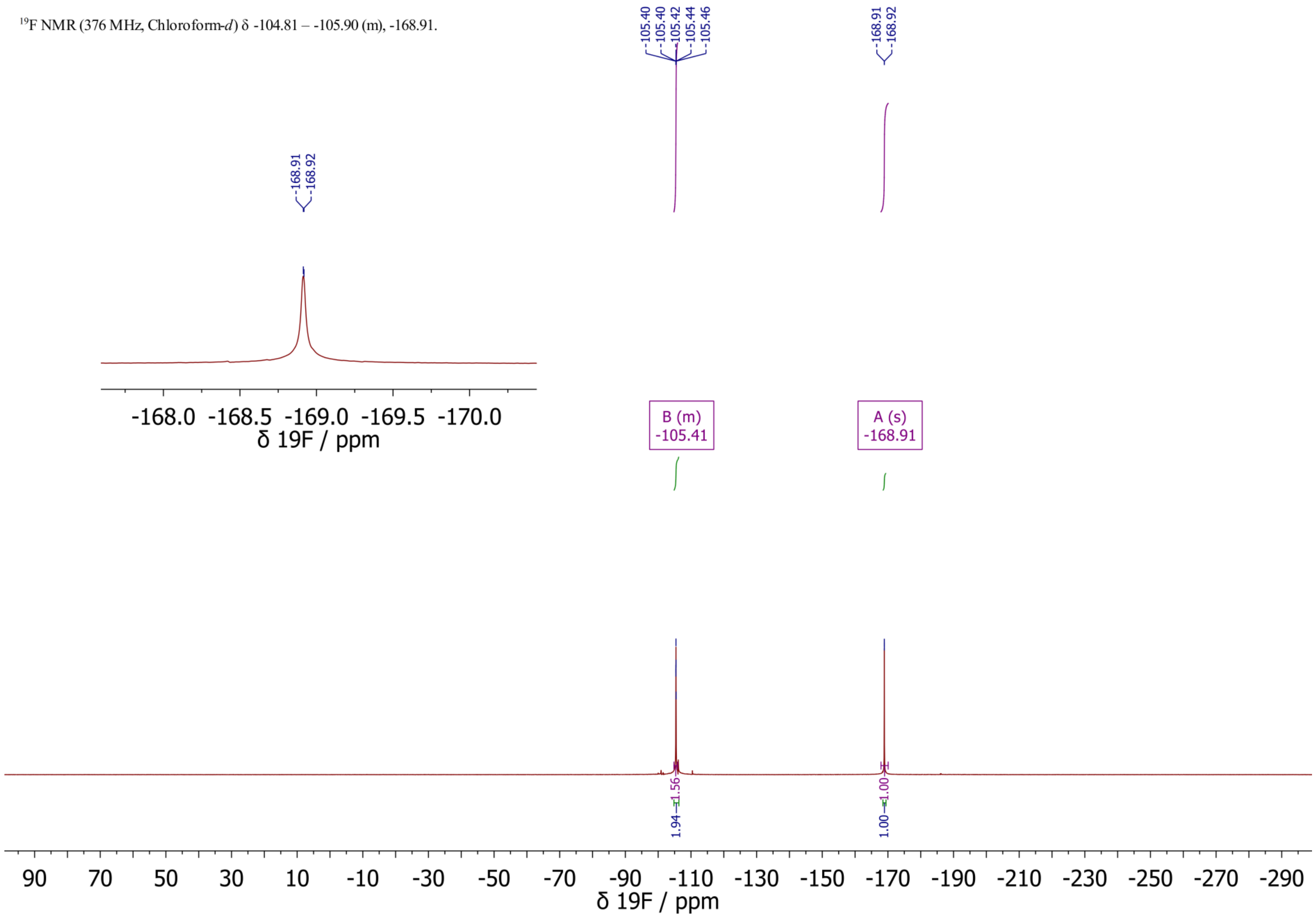

2.4.2 2-fluoro-1,3-bis(4'-methylphenyl)-1,3-propanedione **2c**

Keto-Enol mixture:

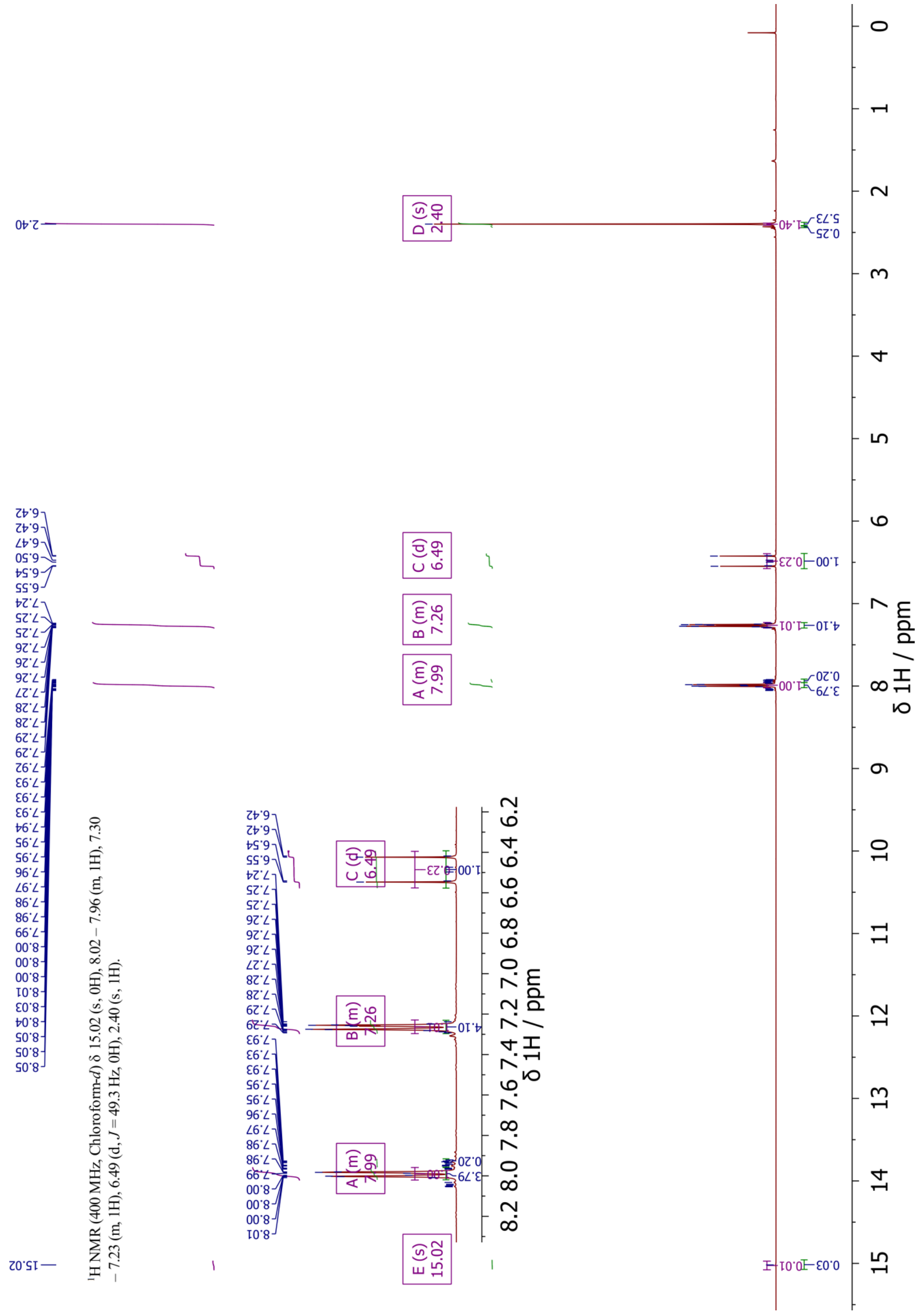

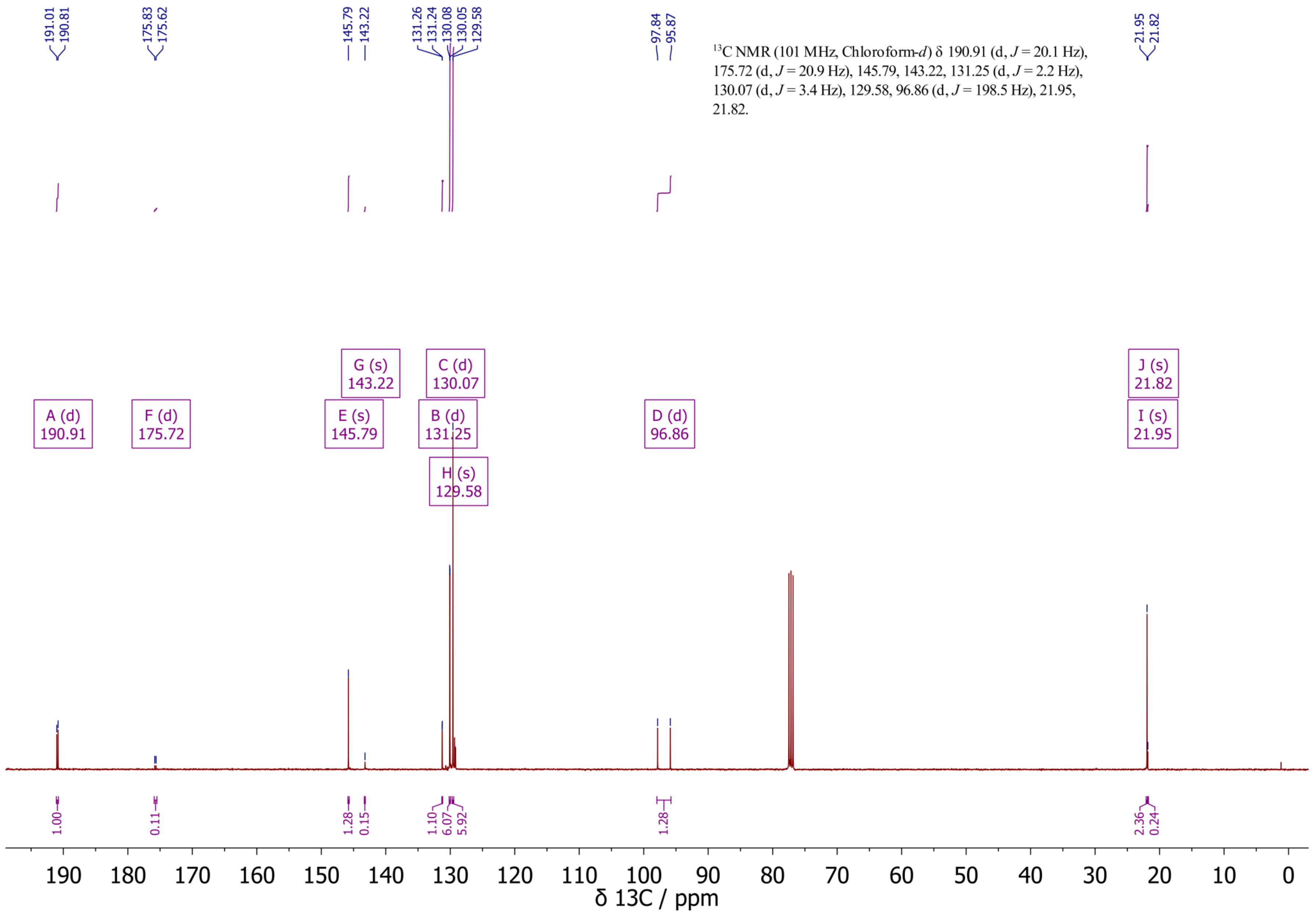

^{13}C NMR (101 MHz, Chloroform-*d*) δ 190.91 (d, J = 20.1 Hz), 175.72 (d, J = 20.9 Hz), 145.79, 143.22, 131.25 (d, J = 2.2 Hz), 130.07 (d, J = 3.4 Hz), 129.58, 96.86 (d, J = 198.5 Hz), 21.95, 21.82.

^{19}F NMR (376 MHz, Chloroform-*d*) δ -168.80, -186.76 (dp, J = 49.3, 1.1 Hz).

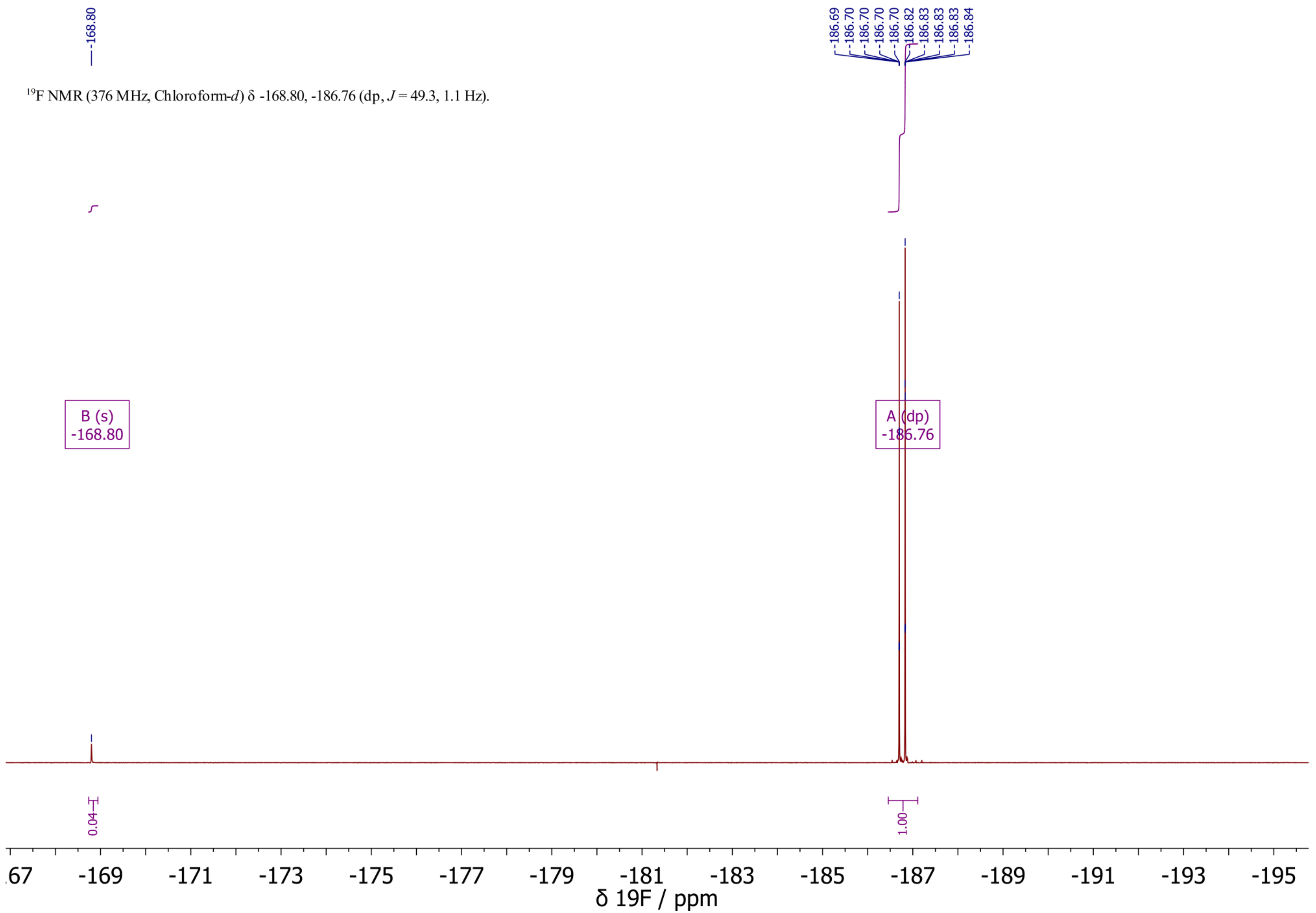

2.4.3 2-fluoro-1,3-bis(4’-chlorophenyl)-1,3-propanedione **2e**

Enol tautomer:

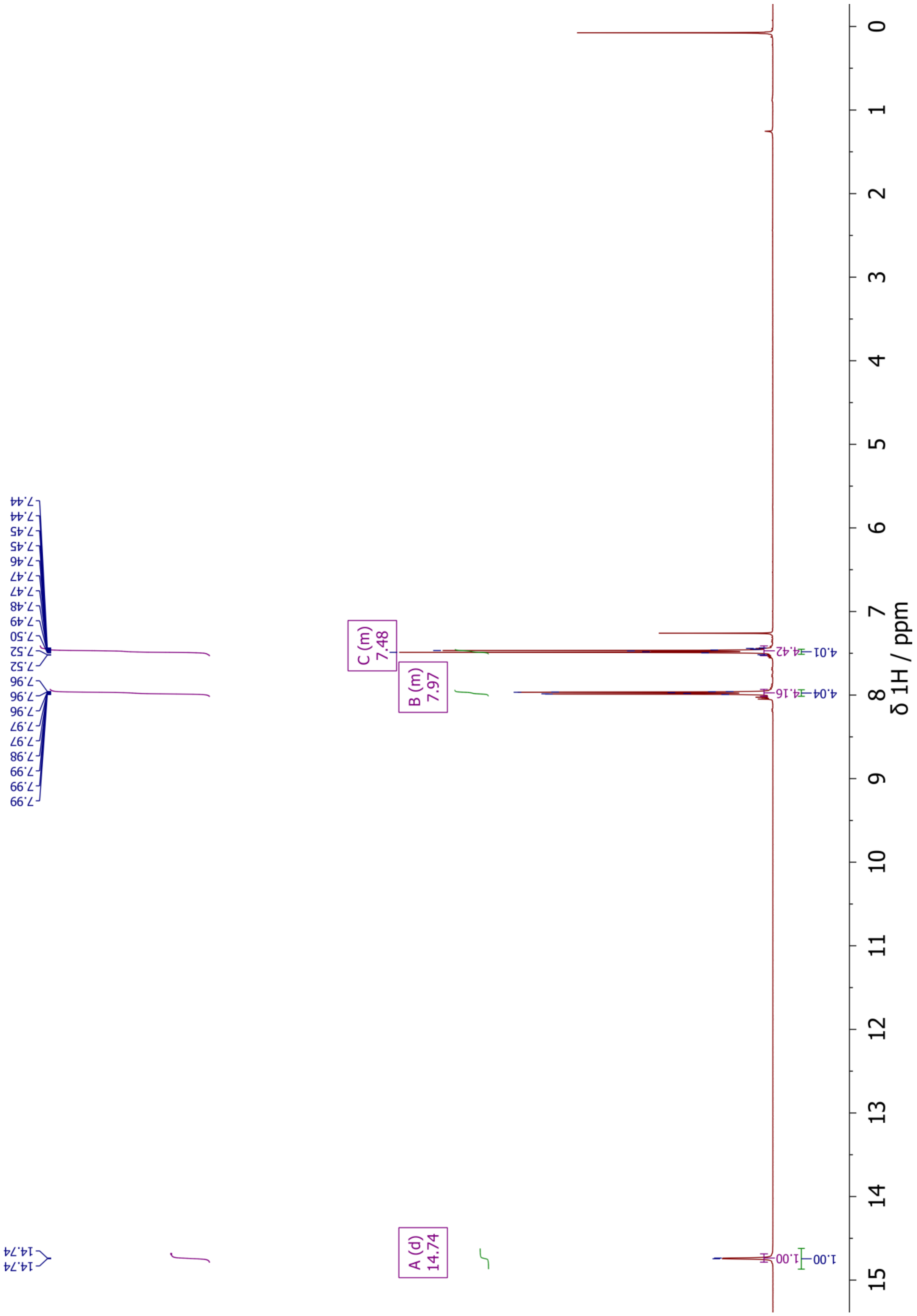

^{13}C NMR (101 MHz, Chloroform-*d*) δ 174.99 (d, *J* = 21.2 Hz), 144.47 (d, *J* = 236.9 Hz), 139.29 (d, *J* = 1.8 Hz), 131.72 (d, *J* = 4.9 Hz), 130.82 (d, *J* = 9.0 Hz), 129.24.

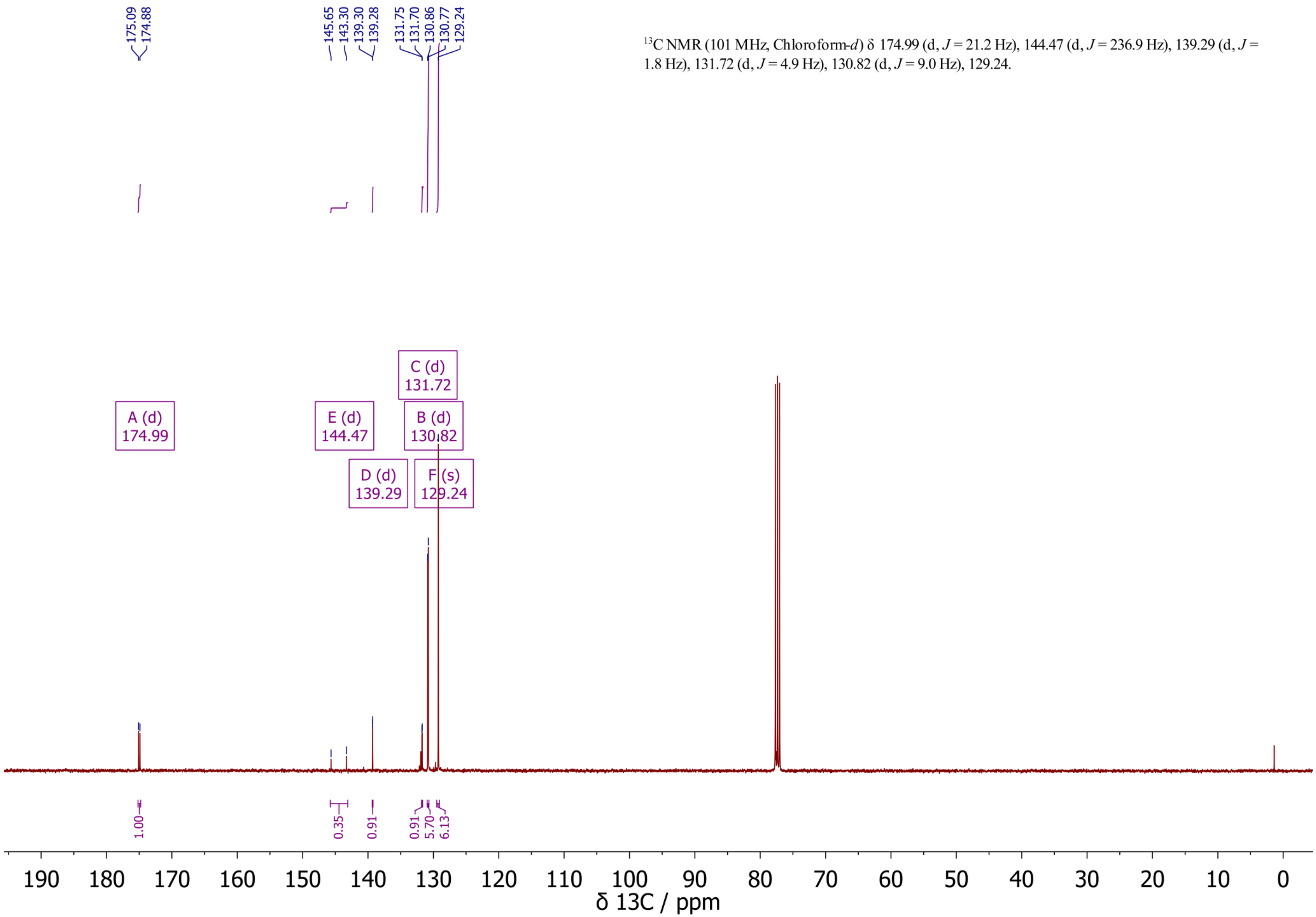

^{19}F NMR (376 MHz, Chloroform-*d*) δ -168.00.

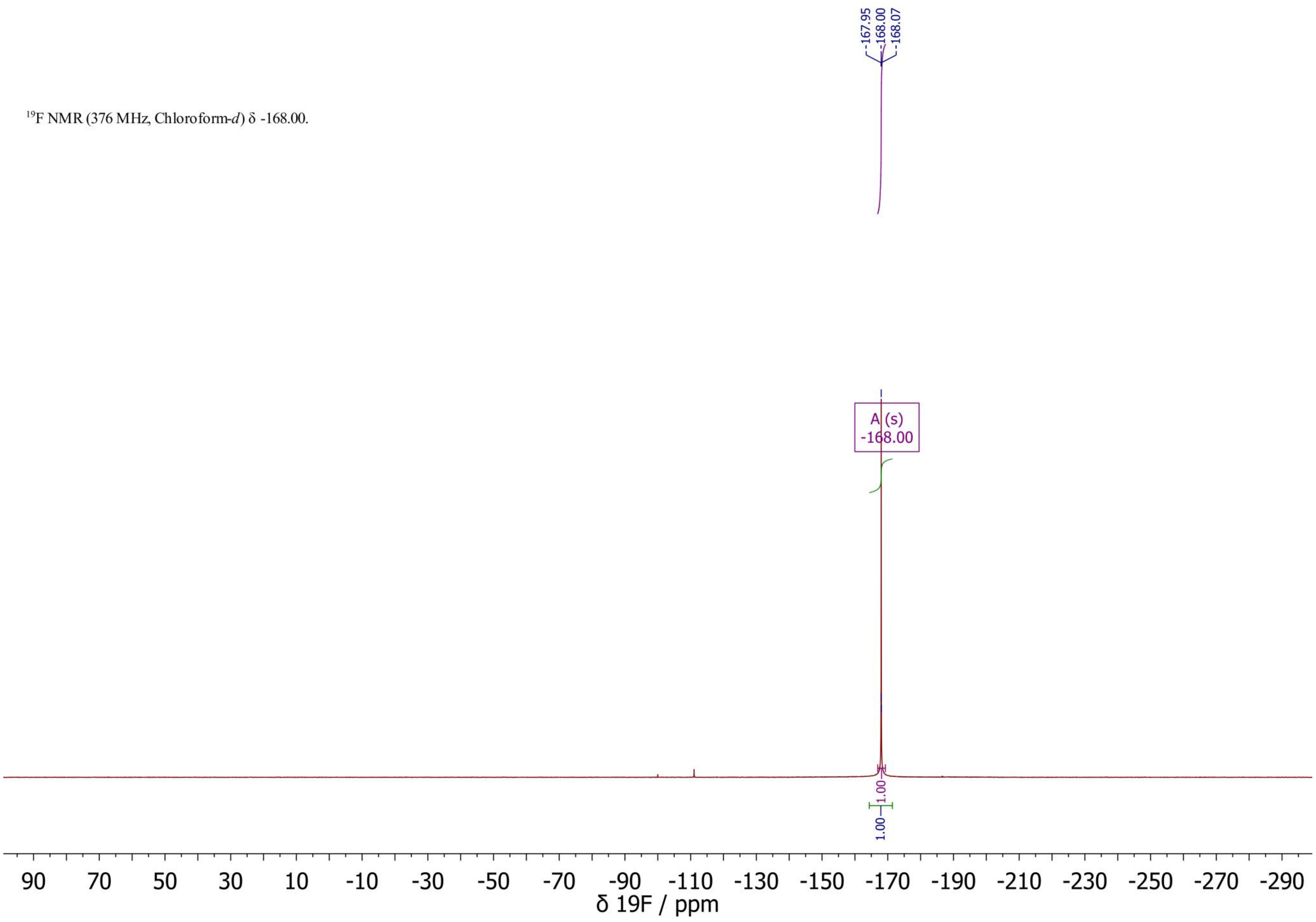

Keto-Enol mixture:

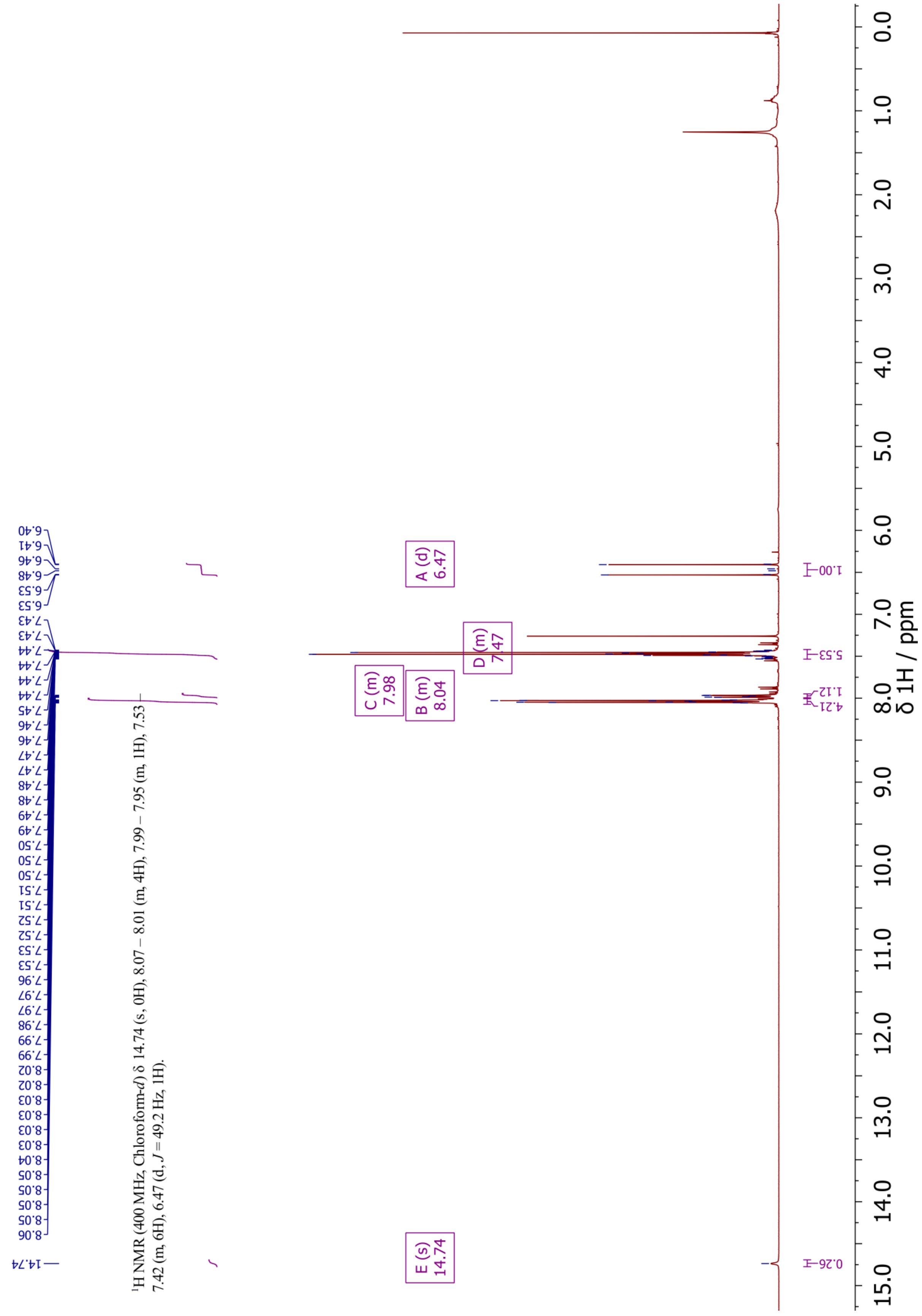

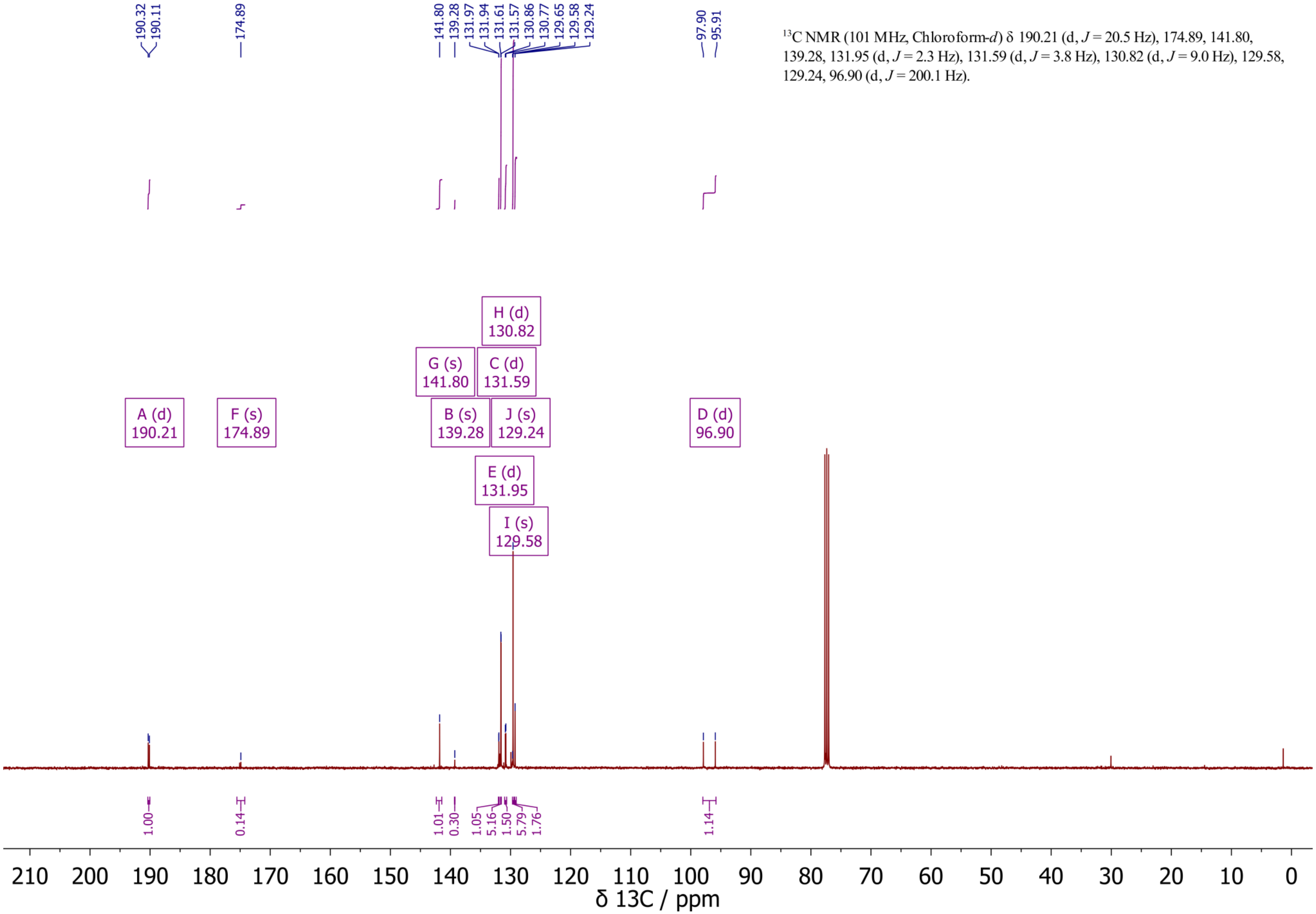

^{13}C NMR (101 MHz, Chloroform-*d*) δ 190.21 (d, J = 20.5 Hz), 174.89, 141.80, 139.28, 131.95 (d, J = 2.3 Hz), 131.59 (d, J = 3.8 Hz), 130.82 (d, J = 9.0 Hz), 129.58, 129.24, 96.90 (d, J = 200.1 Hz).

^{19}F NMR (376 MHz, Chloroform-*d*) δ -168.01, -186.58 (d, J = 49.8 Hz).

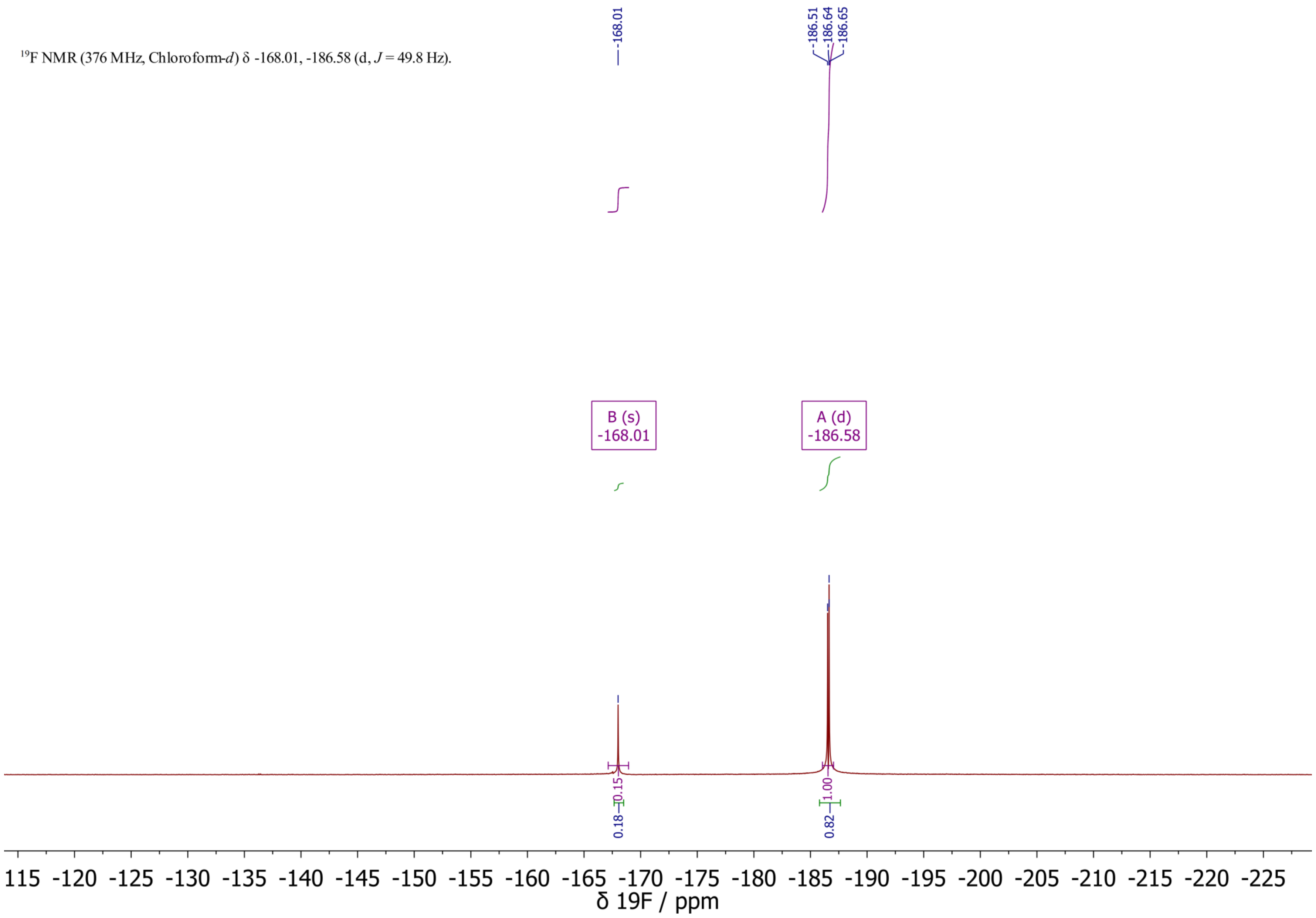

2.4.4 2-fluoro-1,3-bis(4-cyanophenyl)-1,3-propanedione **2f**

Keto-Enol mixture:

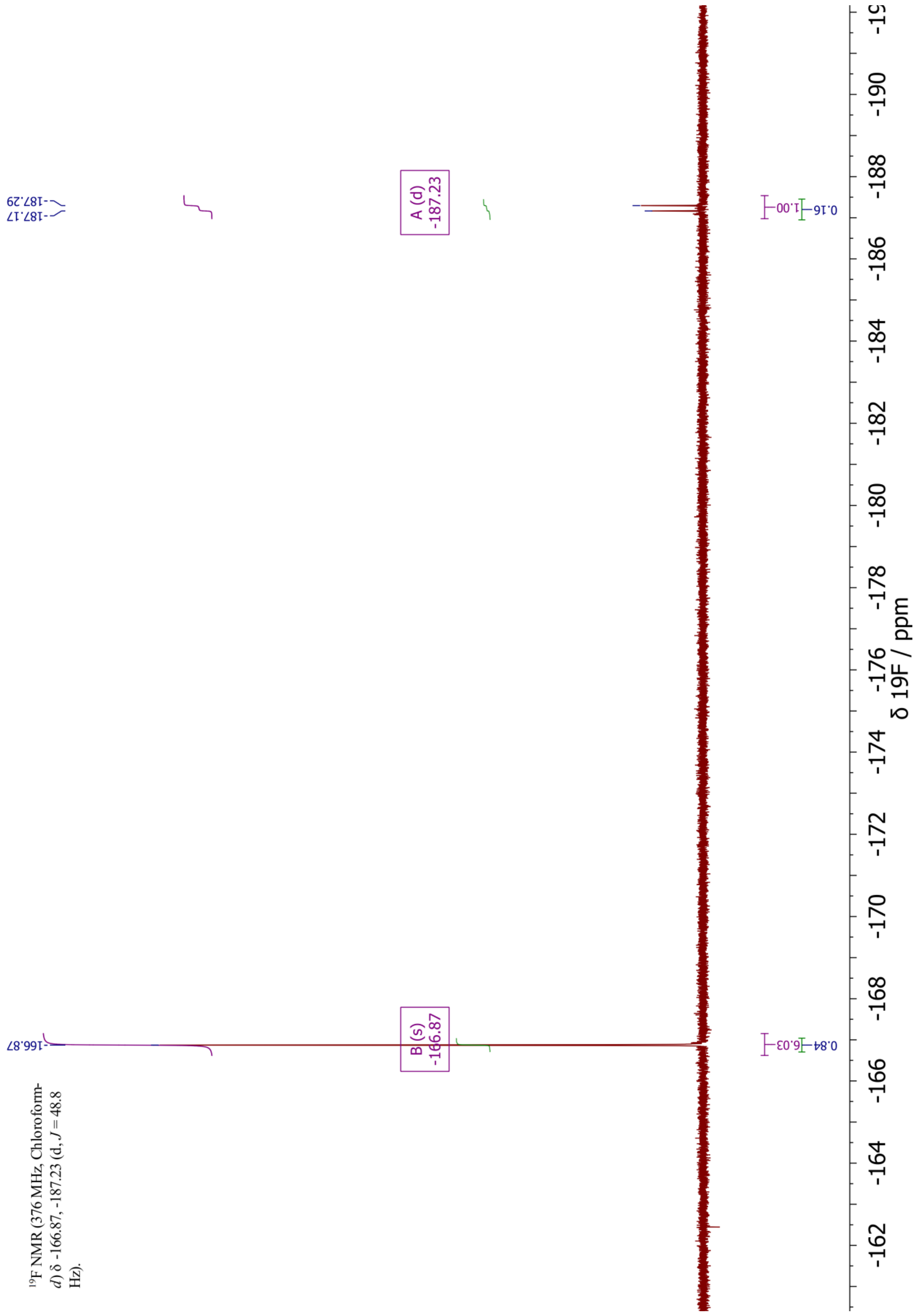

2.5 Distinguishing Fluoro-Keto and Fluoro-Enol Forms by NMR

By ^{19}F NMR (in $CDCl_3$):

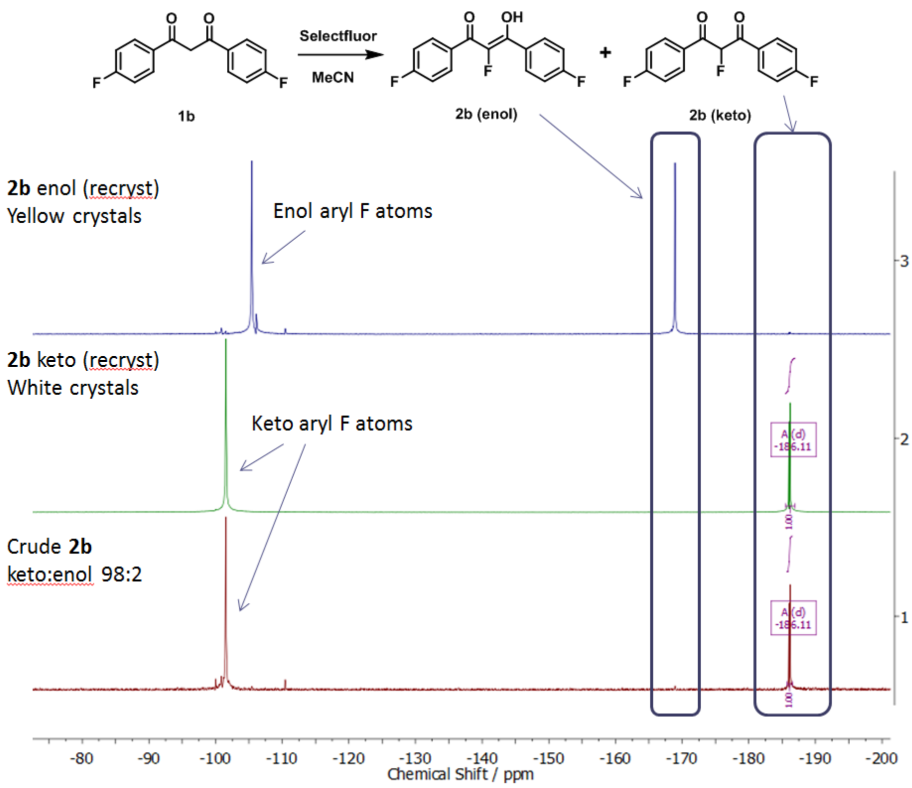

By ^{1}H NMR (in $CDCl_3$):

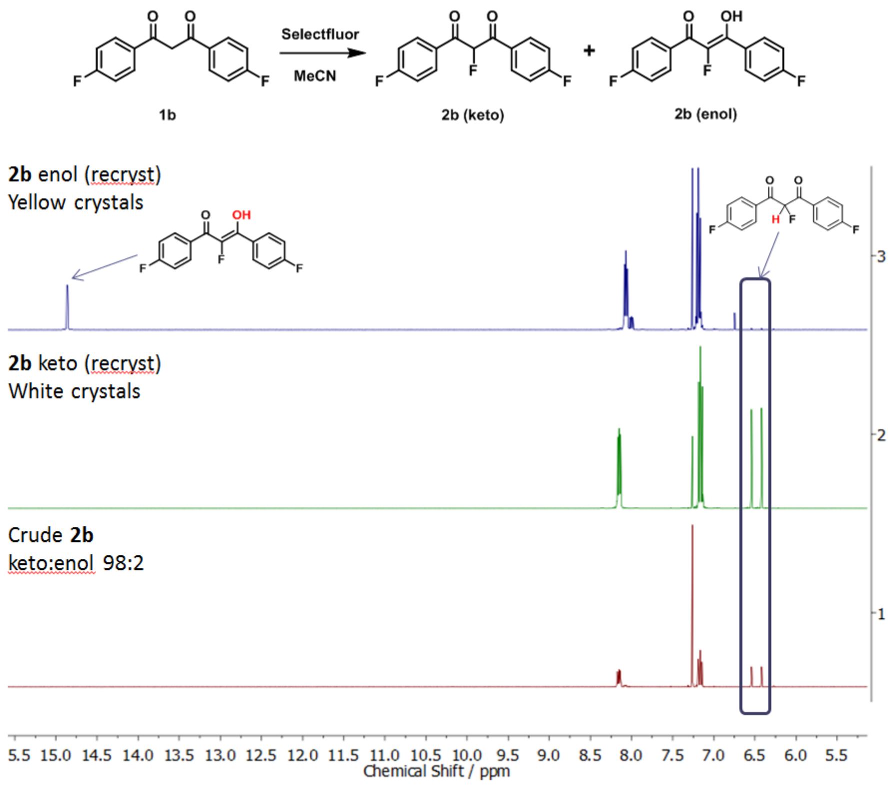

2.6 Keto:Enol Ratios for Compounds 1a-m and 2a-e

Keto:enol ratios for compounds **1a-m** (in CD_3CN) and **2a-e** (in $CDCl_3$) were determined by ^1H and ^{19}F NMR, which gave corroborating ratios, and are shown in **Table 1**. All compounds were purified by recrystallization. Further recrystallization or vapour diffusion crystallization gave the individual crystals reported in the previous sections. Compounds **2a-d** were present mainly in their ketonic forms, with **2e** containing slightly more of the enol form. Compound **2f** was obtained as mainly the enol tautomer. The crystallization conditions and solvent greatly affect the tautomeric ratios.

Table 1: Keto:enol ratios for recrystallized compounds 1a-m in CD_3CN and 2a-e in $CDCl_3$, determined by relative peak integrals in ^1H NMR.

Compound	R Groups	Keto:Enol Ratio
**1a**	$R_1 = R_2 = H$	15:85
**1b**	$R_1 = R_2 = F$	15:85
**1c**	$R_1 = R_2 = Me$	14:86
**1d**	$R_1 = R_2 = OMe$	20:80
**1e**	$R_1 = R_2 = Cl$	7:93
**1f**	$R_1 = R_2 = CN$	5:95
**1g**	$R_1 = R_2 = NO_2$	10:90
**1h**	$R_1 = R_2 = NMe_2$	40:60
**1i**	$R_1 = H, R_2 = F$	16:84
**1j**	$R_1 = H, R_2 = Me$	16:84
**1k**	$R_1 = H, R_2 = OMe$	13:87
**1l**	$R_1 = H, R_2 = Cl$	13:87
**1m**	$R_1 = H, R_2 = NO_2$	9:91
**2a**	$R_1 = R_2 = H$	98:2
**2b**	$R_1 = R_2 = F$	98:2
**2c**	$R_1 = R_2 = Me$	97:3
**2d**	$R_1 = R_2 = OMe$	98:2
**2e**	$R_1 = R_2 = Cl$	82:18
**2f**	$R_1 = R_2 = CN$	16:84

3. Computational Methods

Geometry optimisations were carried out on the keto and enol monomers and dimers of **2b** in the gas phase with the B3LYP[9-10] functional and the 6-311++G**[11-12] basis set using the software package GAUSSIAN09.[13] These optimised geometries were confirmed as true minima by frequency calculations. Single point energy calculations with the Gaussian09 default polarisation continuum solvent model (IEF-PCM)[14] at B3LYP/6-311++G** were performed on the optimised gas-phase geometries with a dielectric constant of ε = 3 as the average dielectric constant for neutral organic crystals.[15] Dielectric constants of ε = 0 and 11 were also applied to assess the effect of solvent polarities on the relative energies (**Table 2**). The procedures here are identical to calculations reported for tautomers elsewhere.[16]

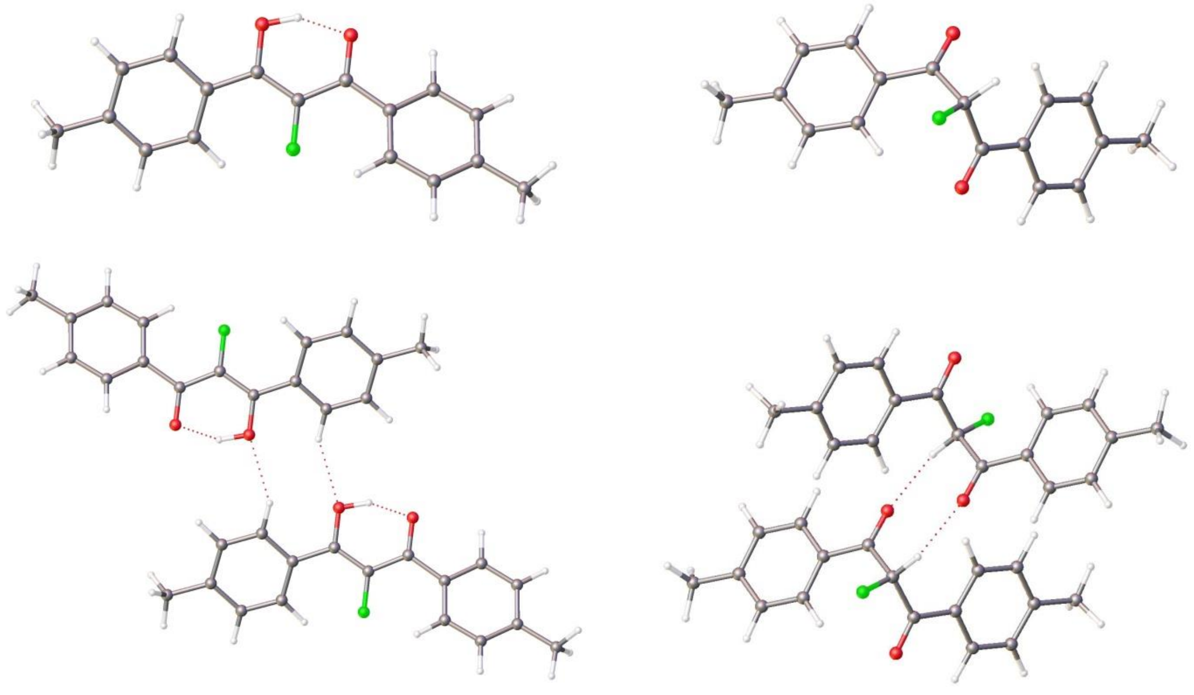

Figure 1: Fully optimised geometries for monomers and dimers as enol and keto forms of 2b. Intermolecular H…O distances in angstroms are 2.623 for enol dimer and 2.408 for keto dimer.

Table 2: Relative energies in kJ mol^{-1} of tautomers of 2b at B3LYP/6-311++G.**

**monomer**	**ε = 0**	**ε = 3**	**ε = 11**
**enol**	0	0	0.9
**keto**	7.5	2.0	0

**dimer**	**ε = 0**	**ε = 3**	**ε = 11**
**enol**	0	2.0	6.4
**keto**	6.6	0	0

4. X-ray Crystallography

The X-ray single crystal data were collected using λMoKα radiation (λ =0.71073Å) at 120.0(2)K on a Bruker SMART CCD 6000 (graphite monochromator, fine-focus tube, Monocap optics) (compounds **2c**) and a Bruker D8Venture (Photon100 CMOS detector, IµS-microsource, focusing mirrors) (all other compounds) diffractometers equipped with a Cryostream (Oxford Cryosystems) open-flow nitrogen cryostats. All structures were solved by the direct method and refined by full-matrix least squares on F^2 for all data using Olex2[17] and SHELXTL[18] software. All non-disordered non-hydrogen atoms were refined anisotropically, hydrogen atoms were refined isotropically, however, the hydrogen atoms in structures **2c-enol** and **2a** were placed in the calculated positions and refined in riding mode. Molecule **2a** showed whole molecule disorder and all atoms were refined with fixed SOF=0.5. The structure **2c-enol** has already been described in literature[19-20] (the CCDC ref. codes FAXWAD and FAXWAD1 respectively), but the structure determination was carried out again for consistency.

Crystal data and parameters of refinement are listed in **Table 3**. Crystallographic data for the structure have been deposited with the Cambridge Crystallographic Data Centre as a supplementary publication CCDC-1857922-1857928.

Table 3: Crystal data and structure refinement

Identification code	**2aK**	**2bK**	**2bE**	**2cK**	**2cE**	**2dE**	**2eE**
Empirical formula	$C_{15}H_{11}FO_2$	$C_{15}H_9F_3O_2$	$C_{15}H_9F_3O_2$	$C_{17}H_{15}FO_2$	$C_{17}H_{15}FO_2$	$C_{17}H_{15}FO_4$	$C_{15}H_9Cl_2FO_2$
Formula weight	242.24	278.22	278.22	270.29	270.29	302.29	311.12
Crystal system	monoclinic	monoclinic	monoclinic	monoclinic	monoclinic	orthorhombic	orthorhombic
Space group	I2/a	$P2_1$	C2/c	$P2_1/c$	C2/c	$Cmc2_1$	Pnma
a/Å	13.2572(6)	4.3647(4)	28.403(2)	8.5592(5)	11.1604(8)	31.944(2)	6.0944(8)
b/Å	5.1221(2)	11.5018(10)	6.1029(4)	12.9739(8)	11.7655(8)	7.0229(5)	30.676(4)
c/Å	16.7741(8)	12.3118(11)	6.9953(5)	12.3376(8)	10.8190(8)	6.1827(5)	6.8810(9)
β/°	97.558(2)	94.065(3)	102.753(3)	102.4067(17)	114.3180(17)	90	90
Volume/$Å^3$	1129.15(9)	616.52(10)	1182.67(14)	1338.05(14)	1294.57(16)	1387.02(18)	1286.4(3)
Z	4	2	4	4	4	4	4
$\rho_{calc}g/cm^3$	1.425	1.499	1.563	1.342	1.387	1.448	1.606
μ/mm^{-1}	0.105	0.129	0.134	0.096	0.099	0.111	0.513
F(000)	504	284.0	568.0	568.0	568.0	632.0	632.0
Reflections collected	10000	13411	12032	22309	7788	11975	20698
Independent refl., R_{int}	1359, 0.0271	3582 0.0339	1724, 0.0345	3569, 0.0452	1646, 0.0298	1880, 0.0443	1750, 0.0444
Data/restraints/parameters	1359/132/158	3582/1/217	1724/0/112	3569/0/241	1646/0/94	1880/1/131	1750/0/113
Goodness-of-fit on F^2	1.113	1.044	1.054	1.086	1.105	1.042	1.213
Final R_1 indexes [I≥2σ(I)]	0.0342	0.0415	0.0415	0.0580	0.0721	0.0370	0.0436
Final wR_2 indexes [all data]	0.0884	0.0981	0.1230	0.1749	0.2243	0.0994	0.0971
Largest diff. peak/hole / e $Å^{-3}$	0.20/-0.22	0.22/-0.21	0.48/-0.26	0.51/-0.20	0.88/-0.54	0.37/-0.38	0.33/-0.30
Flack parameter	n/a	n/a	n/a	n/a	n/a	0.1(3)	n/a

5. Kinetics Conducted by UV-Vis Spectrophotometry

5.1 Methods

Kinetics studies were carried out using a Varian Cary-100 Bio UV/Vis Spectrophotometer equipped with a Cary Temperature Controller unit, or a Varian Cary-50 Bio UV/Vis Spectrophotometer connected to a Varian Cary PCB-150 Water Peltier system. Samples were contained in quartz absorption cuvettes with a path length of 1 cm. All spectra were zeroed against air. Reactions were followed by monitoring the disappearance of the enol at a fixed wavelength corresponding to the maximum absorbance (λ_{max}) of the relevant enol (**Table 4**). All reactions were carried out under pseudo-first-order conditions in the presence of excess Selectfluor™. Error values quoted in Section 5.4 are standard error values obtained from data fitting in KaleidaGraph software. Stock solutions of purified nucleophiles **1a-m** (5-10 mM) and fluorinating reagents **3**-**9** (5-180 mM) in MeCN (HPLC grade) were prepared in volumetric flasks. For kinetics studies involving water-sensitive NF reagents (**5**, **8a** and **8b**), MeCN was distilled from CaH_2 immediately before use. Aliquots of each stock solution were removed and diluted accordingly to the desired concentration. These solutions were transferred to two separate cuvettes which were placed in the spectrophotometer for 10 mins to equilibrate to the required temperature. 1.5 mL of the nucleophile and 1.5 mL of F^+ were then mixed and the cuvette immediately returned to the spectrophotometer. Using the scanning kinetics or single wavelength kinetics programs, the kinetics studies were carried out.

Table 4: λ_{max} (enol) values for compounds 1a-m, in MeCN.

Compound	λ_{max} (enol)/ nm
**1a**	341
**1b**	341
**1c**	350
**1d**	362
**1e**	347
**1f**	351
**1g**	363
**1h**	425
**1i**	341
**1j**	347
**1k**	352
**1l**	345
**1m**	355

5.2 Hammett Correlations for Selectfluor™

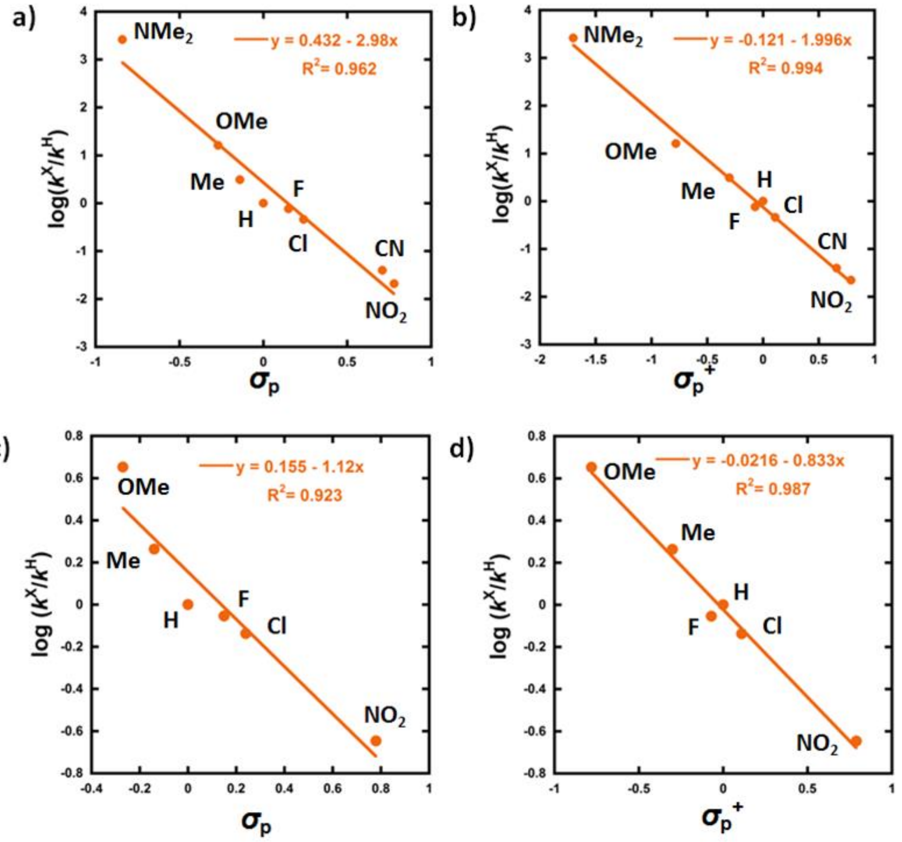

Figure 2: Hammett plots for reactions of Selectfluor™ with 1a-h correlated against: a) σ_p values, and b) σ_p^+ values. Hammett plots for reactions of Selectfluor™ with 1i-m correlated against: c) σ_p values, and b) σ_p^+ values. Rate constants all at 20 °C were used to obtain Hammett plots for di-substituted nucleophiles, and for mono-substituted nucleophiles, all rate constants were at 25 °C.

5.3 Determination of Activation Parameters for the Reaction of Selectfluor™ with Nucleophiles 1a-e

Second order rate constants for the reactions of compounds **1a-e** with Selectfluor™ were determined as described above. The linear form of the Eyring equation was used to calculate activation parameters, where the slope of the linear plot of ln(k_2/T) vs 1/T is equal to $-\Delta H^{\ddagger}$/R. The entropy of activation, $\Delta S^{\ddagger}$, was calculated from the intercept of the linear plot, i.e. ln(k_B/h) + $\Delta S^{\ddagger}/R$. The values for $\Delta G^{\ddagger}$ were calculated from the Boltzmann equation. The constants k, R, T, k_B and h represent the rate constant k_2, gas constant, absolute temperature, Boltzmann constant and Planck's constant, respectively.

$$ln\frac{k}{T} = \frac{-\Delta H^{\ddagger}}{RT} + \ln\left(\frac{k_B}{h}\right) + \frac{\Delta S^{\ddagger}}{R} \quad (\mathbf{1})$$

$$\Delta G^{\ddagger} = \Delta H^{\ddagger} - T\Delta S^{\ddagger} \quad (\mathbf{2})$$

5.4 Kinetics Reactions Involving Selectfluor™ (3) at 4 Different Temperatures

5.4.1 Nucleophile **1a**

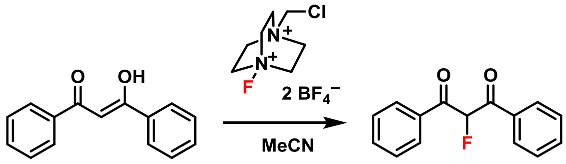

1a → **2a**

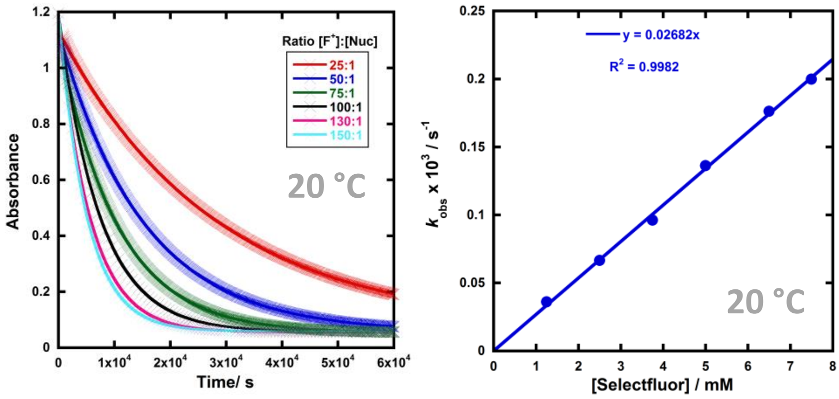

Table 5: k_{obs} **values at different concentrations of Selectfluor™ at 20 °C. Errors are standard error values.**

Experiment	Ratio of F^+ : Nuc	$[F^+]$: [Nuc]/ mM	$k_{obs} \times 10^5$/ s^{-1}
**1**	25:1	1.25 : 0.05	3.611 ± 0.004
**2**	50:1	2.5 : 0.05	6.657 ± 0.003
**3**	75:1	3.75 : 0.05	9.624 ± 0.003
**4**	100:1	5.0 : 0.05	13.626 ± 0.009
**5**	130:1	6.5 : 0.05	17.632 ± 0.013
**6**	150:1	7.5 : 0.05	19.984 ± 0.016

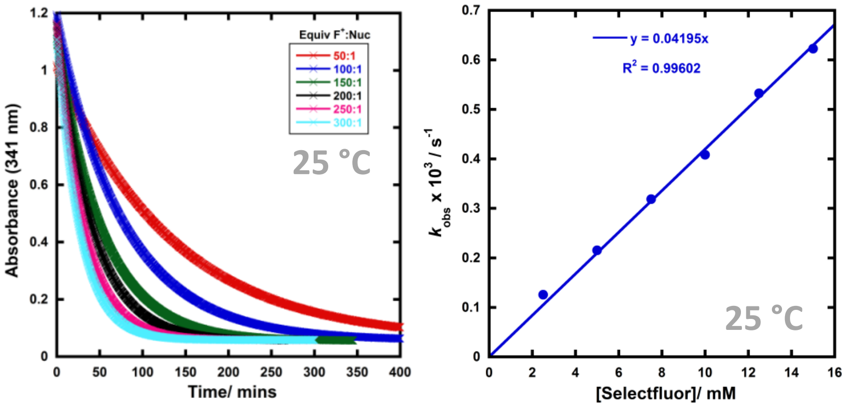

Table 6: k_{obs} values at different concentrations of Selectfluor™ at 25 °C. Errors are standard error values.

Experiment	Ratio of F^+ : Nuc	$[F^+]$: [Nuc]/ mM	$k_{obs} \times 10^4$/ s^{-1}
**1**	50:1	2.5 : 0.05	1.2574 ± 0.0003
**2**	100:1	5.0 : 0.05	2.1523 ± 0.0003
**3**	150:1	7.5 : 0.05	3.1863 ± 0.0003
**4**	200:1	10.0 : 0.05	4.0851 ± 0.0008
**5**	250:1	12.5 : 0.05	5.3238 ± 0.0008
**6**	300:1	15.0 : 0.05	6.2273 ± 0.0009

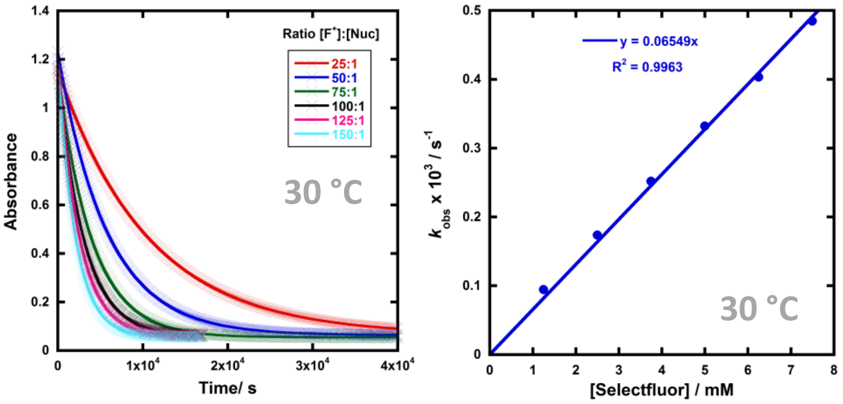

Table 7: k_{obs} values at different concentrations of Selectfluor™ at 30 °C.

Experiment	Ratio of F^+ : Nuc	$[F^+]$: [Nuc]/ mM	$k_{obs} \times 10^4$/ s^{-1}
**1**	25:1	1.25 : 0.05	0.9438 ± 0.0003
**2**	50:1	2.5 : 0.05	1.7361 ± 0.0006
**3**	75:1	3.75 : 0.05	2.5188 ± 0.0009
**4**	100:1	5.0 : 0.05	3.322 ± 0.001
**5**	125:1	6.25 : 0.05	4.032 ± 0.001
**6**	150:1	7.5 : 0.05	4.847 ± 0.001

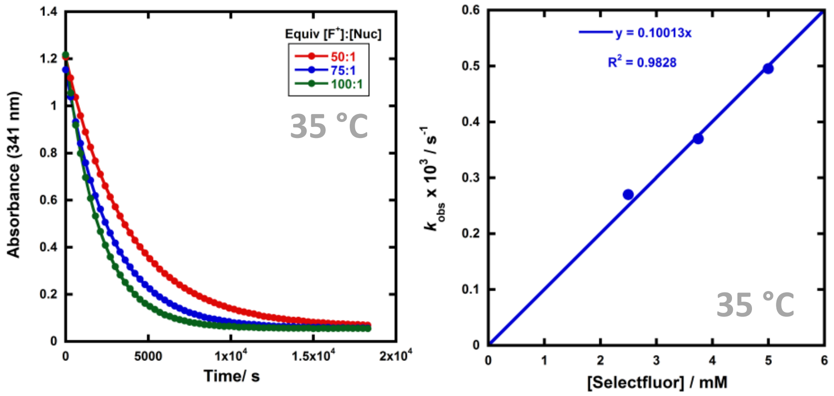

Table 8: k_{obs} values at different concentrations of Selectfluor™ at 35 °C.

Experiment	Ratio of F^+ : Nuc	$[F^+]$: [Nuc]/ mM	$k_{obs} \times 10^3 / s^{-1}$
**1**	50:1	2.5 : 0.05	0.2698 ± 0.0002
**2**	75:1	3.75 : 0.05	0.3697 ± 0.0003
**3**	100:1	5.0 : 0.05	0.4953 ± 0.0004

5.4.2 Nucleophile **1b**

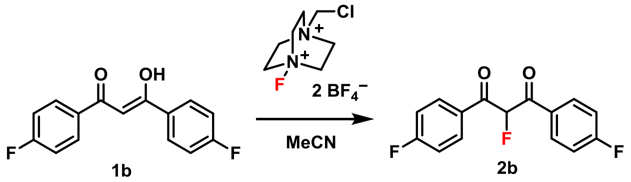

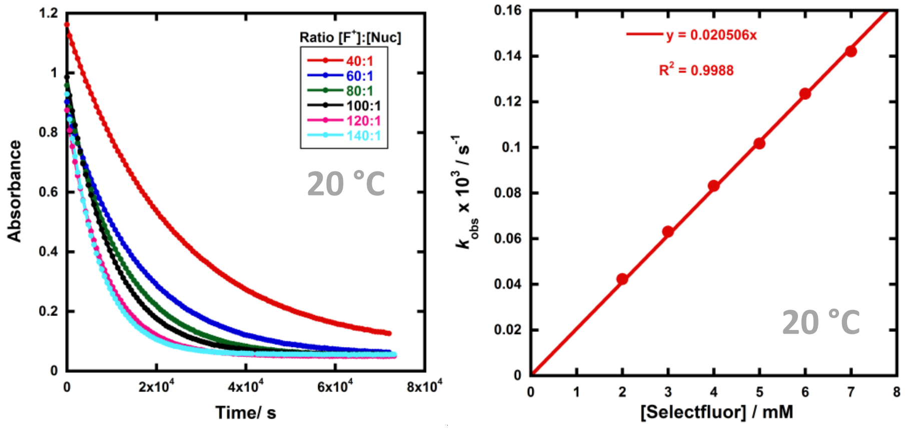

Table 9: k_{obs} values at different concentrations of Selectfluor™ at 20 °C.

Experiment	Ratio of F^+ : Nuc	$[F^+]$: [Nuc]/ mM	$k_{obs} \times 10^4$ / s^{-1}
**1**	40:1	2.0 : 0.05	0.4228 ± 0.0006
**2**	60:1	3.0 : 0.05	0.631 ± 0.001
**3**	80:1	4.0 : 0.05	0.832 ± 0.002
**4**	100:1	5.0 : 0.05	1.0166 ± 0.0007
**5**	120:1	6.0 : 0.05	1.236 ± 0.001
**6**	140:1	7.0 : 0.05	1.420 ± 0.002

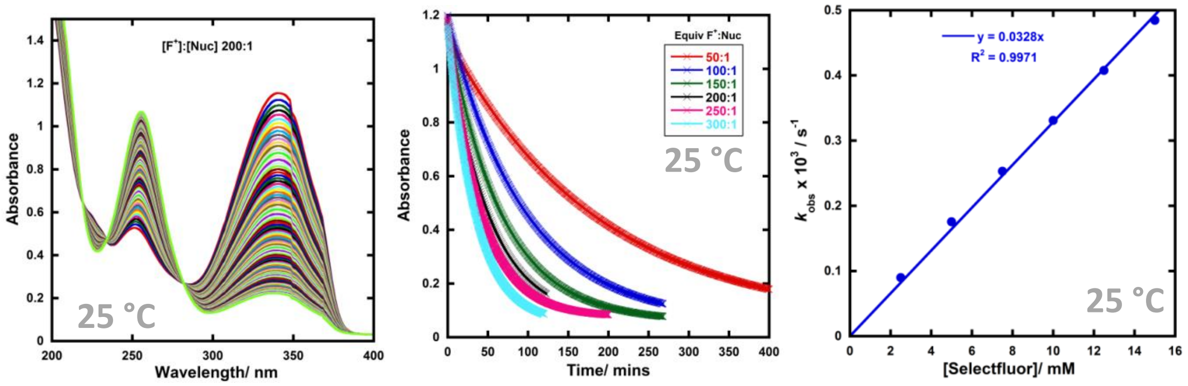

Table 10: k_{obs} values at different concentrations of Selectfluor™ at 25 °C.

Experiment	Ratio of F^+ : Nuc	$[F^+]$: [Nuc]/ mM	$k_{obs} \times 10^4$ / s^{-1}
**1**	50:1	2.50 : 0.05	0.8993 ± 0.0004
**2**	100:1	5.00 : 0.05	1.7559 ± 0.0008
**3**	150:1	7.50 : 0.05	2.5305 ± 0.0008
**4**	200:1	10.0 : 0.05	3.310 ± 0.001
**5**	250:1	12.5 : 0.05	4.076 ± 0.002
**6**	300:1	15.0 : 0.05	4.846 ± 0.002

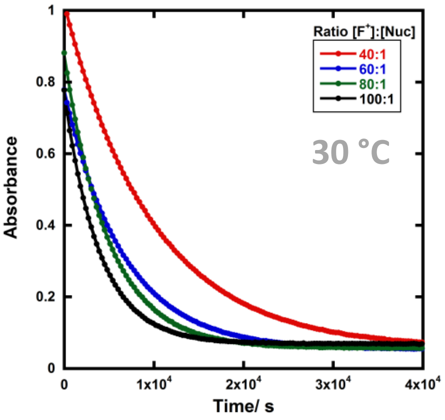

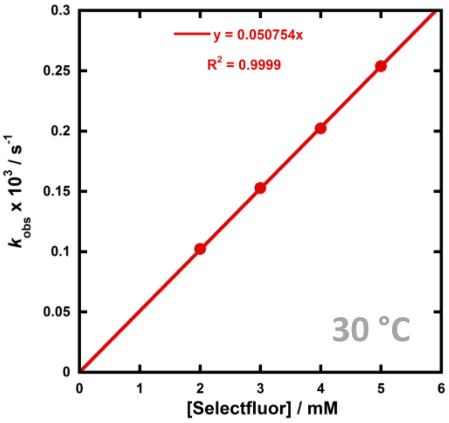

Table 11: k_{obs} **values at different concentrations of Selectfluor™ at 30 °C.**

Experiment	Ratio of F^+ : Nuc	$[F^+]$: [Nuc]/ mM	$k_{obs} \times 10^3$ / s^{-1}
**1**	40:1	2.0 : 0.05	0.1022 ± 0.0005
**2**	60:1	3.0 : 0.05	0.1527 ± 0.0008
**3**	80:1	4.0 : 0.05	0.202 ± 0.001
**4**	100:1	5.0 : 0.05	0.254 ± 0.003

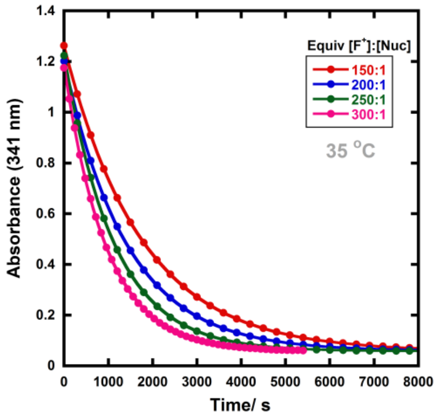

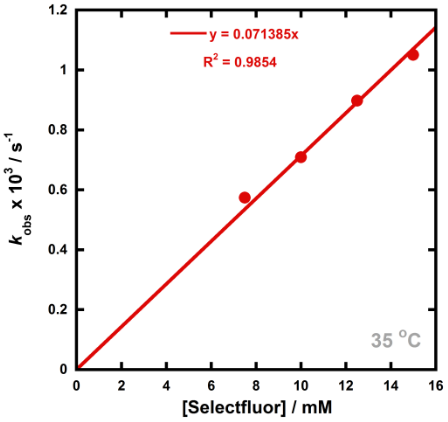

Table 12: k_{obs} **values at different concentrations of Selectfluor™ at 35 °C.**

Experiment	Ratio of F^+ : Nuc	$[F^+]$: [Nuc]/ mM	$k_{obs} \times 10^3$ / s^{-1}
**1**	150:1	7.5 : 0.05	0.5737 ± 0.0003
**2**	200:1	10.0 : 0.05	0.7088 ± 0.0007
**3**	250:1	12.5 : 0.05	0.897 ± 0.002
**4**	300:1	15.0 : 0.05	1.051 ± 0.003

5.4.3 Nucleophile **1c**

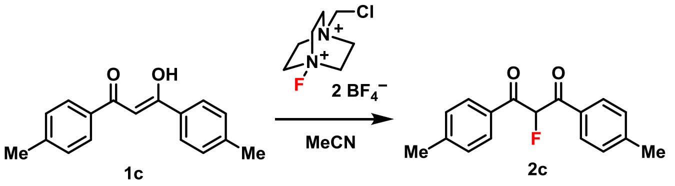

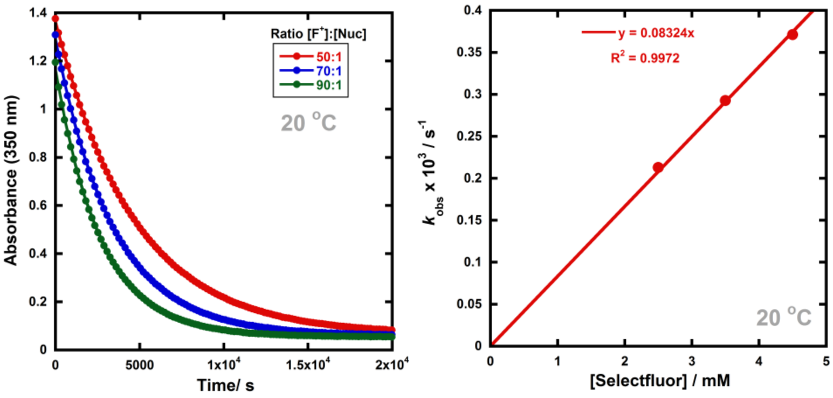

Table 13: k_{obs} values at different concentrations of Selectfluor™ at 20 °C.

Experiment	Ratio of F^+ : Nuc	$[F^+]$: [Nuc]/ mM	$k_{obs} \times 10^3$ / s^{-1}
**1**	50:1	2.5 : 0.05	0.2127 ± 0.0002
**2**	70:1	3.5 : 0.05	0.2925 ± 0.0003
**3**	90:1	4.5 : 0.05	0.3711 ± 0.0006

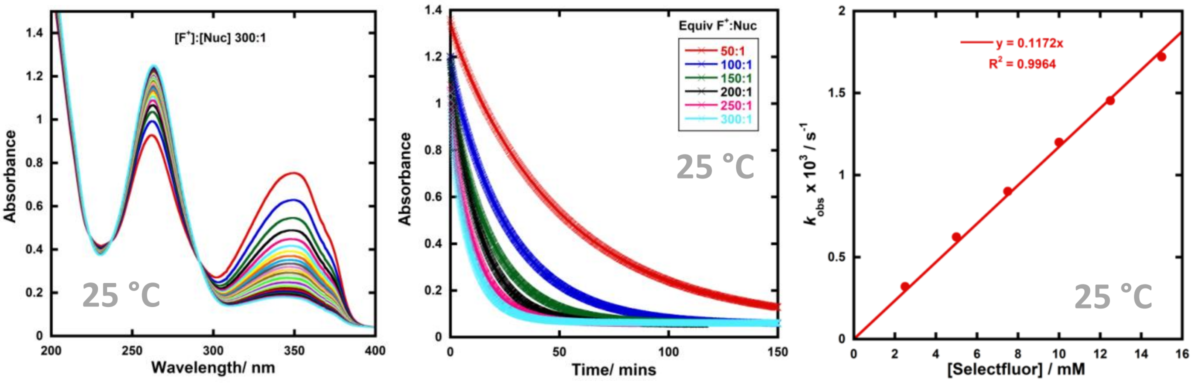

Table 14: k_{obs} values at different concentrations of Selectfluor™ at 25 °C.

Experiment	Ratio of F^+ : Nuc	$[F^+]$: [Nuc]/ mM	$k_{obs} \times 10^3$ / s^{-1}
**1**	50:1	2.50 : 0.05	0.3210 ± 0.0001
**2**	100:1	5.00 : 0.05	0.6235 ± 0.0001
**3**	150:1	7.50 : 0.05	0.9005 ± 0.0003
**4**	200:1	10.0 : 0.05	1.2000 ± 0.0006
**5**	250:1	12.5 : 0.05	1.4538 ± 0.0007
**6**	300:1	15.0 : 0.05	1.721 ± 0.001

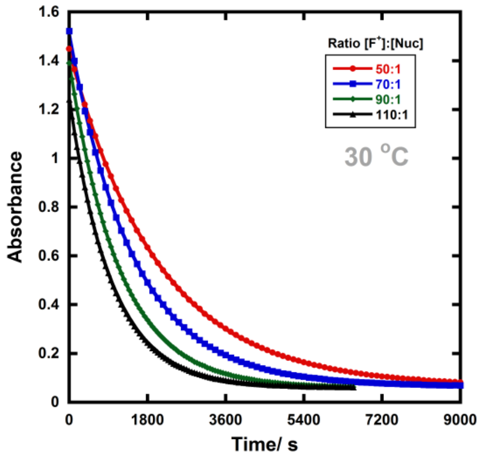

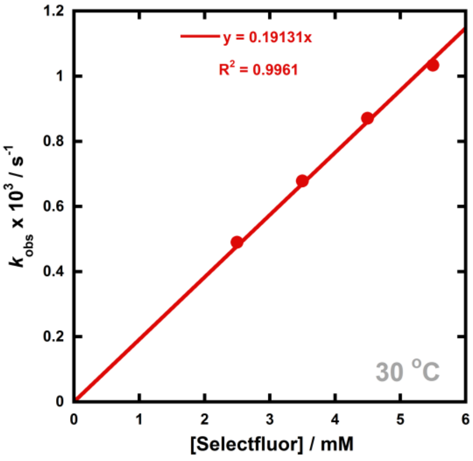

Table 15: k_{obs} values at different concentrations of Selectfluor™ at 30 °C.

Experiment	Ratio of F^+ : Nuc	$[F^+]$: [Nuc]/ mM	$k_{obs} \times 10^3$ / s^{-1}
**1**	50:1	2.50 : 0.05	0.4900 ± 0.0004
**2**	100:1	5.00 : 0.05	0.6783 ± 0.0008
**3**	150:1	7.50 : 0.05	0.8704 ± 0.0005
**4**	200:1	10.0 : 0.05	1.0335 ± 0.0009

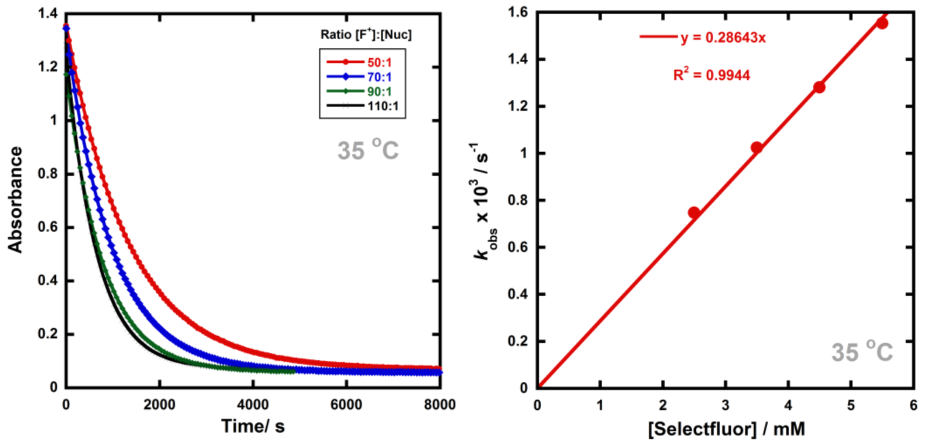

Table 16: k_{obs} values at different concentrations of Selectfluor™ at 35 °C.

Experiment	Ratio of F^+ : Nuc	$[F^+]$: [Nuc]/ mM	$k_{obs} \times 10^3$ / s^{-1}
**1**	50:1	2.50 : 0.05	0.7477 ± 0.0004
**2**	100:1	5.00 : 0.05	1.024 ± 0.001
**3**	150:1	7.50 : 0.05	1.281 ± 0.002
**4**	200:1	10.0 : 0.05	1.554 ± 0.004

5.4.4 Nucleophile **1d**

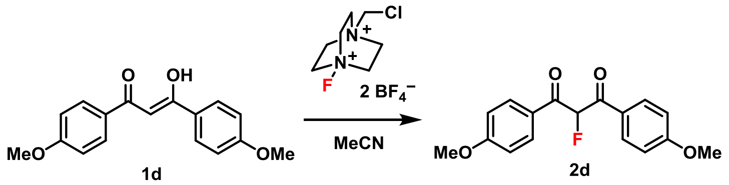

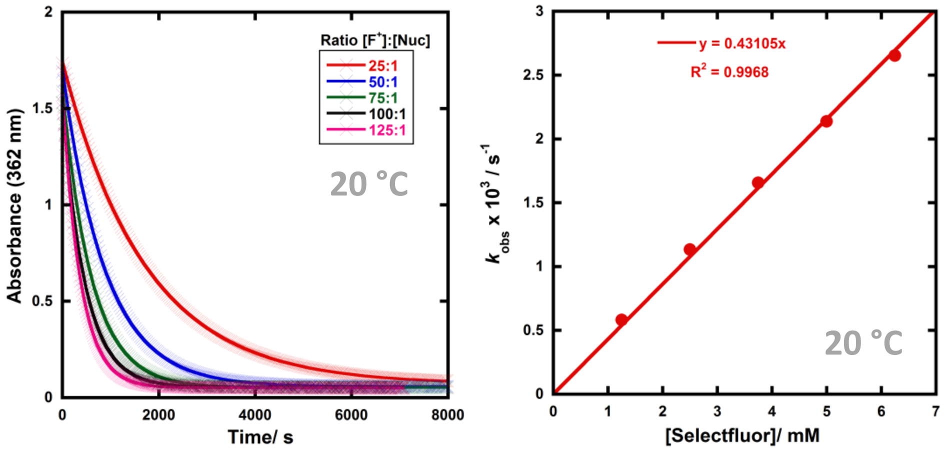

Table 17: k_{obs} values at different concentrations of Selectfluor™ at 20 °C.

Experiment	Ratio of F^+ : Nuc	$[F^+]$: [Nuc]/ mM	$k_{obs} \times 10^3$ / s^{-1}
**1**	25:1	1.25 : 0.05	0.5822 ± 0.0002
**2**	50:1	2.50 : 0.05	1.1333 ± 0.0005
**3**	75:1	3.75 : 0.05	1.6554 ± 0.0007
**4**	100:1	5.00 : 0.05	2.1383 ± 0.0008
**5**	125:1	6.25 : 0.05	2.653 ± 0.002

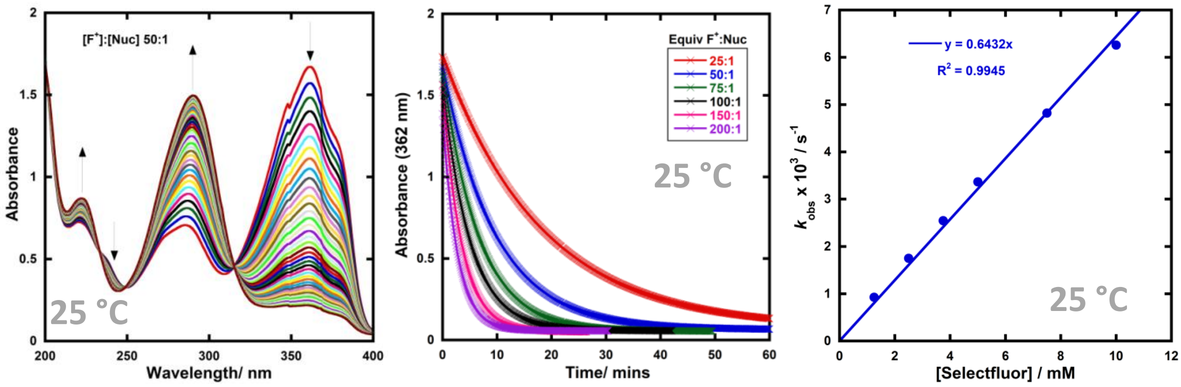

Table 18: k_{obs} values at different concentrations of Selectfluor™ at 25 °C.

Experiment	Ratio of F^+ : Nuc	$[F^+]$: [Nuc]/ mM	$k_{obs} \times 10^3$ / s^{-1}
**1**	25:1	1.25 : 0.05	0.92664 ± 0.0002
**2**	50:1	2.50 : 0.05	1.7505 ± 0.0004
**3**	75:1	3.75 : 0.05	2.546 ± 0.001
**4**	100:1	5.00 : 0.05	3.3674 ± 0.0009
**5**	150:1	7.50 : 0.05	4.821 ± 0.004
**6**	200:1	10.0 : 0.05	6.2563 ± 0.003

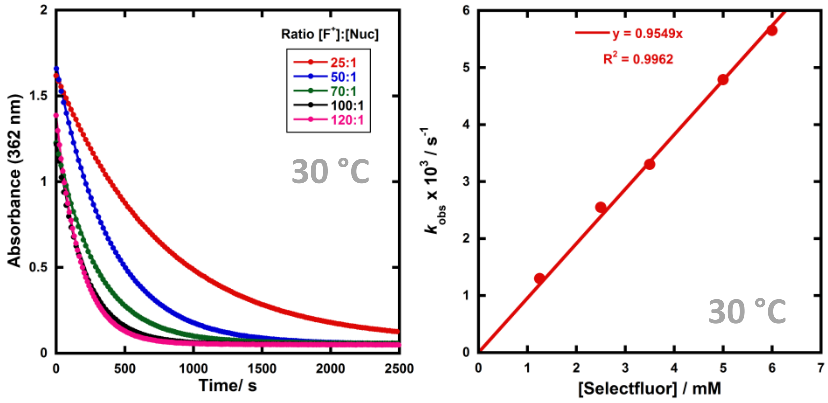

Table 19: k_{obs} values at different concentrations of Selectfluor™ at 30 °C.

Experiment	Ratio of F^+ : Nuc	$[F^+]$: [Nuc]/ mM	$k_{obs} \times 10^3$ / s^{-1}
**1**	25:1	1.25 : 0.05	1.2999 ± 0.0007
**2**	50:1	2.5 : 0.05	2.550 ± 0.002
**3**	70:1	3.5 : 0.05	3.301 ± 0.004
**4**	100:1	5.0 : 0.05	4.787 ± 0.006
**5**	120:1	6.0 : 0.05	5.653 ± 0.005

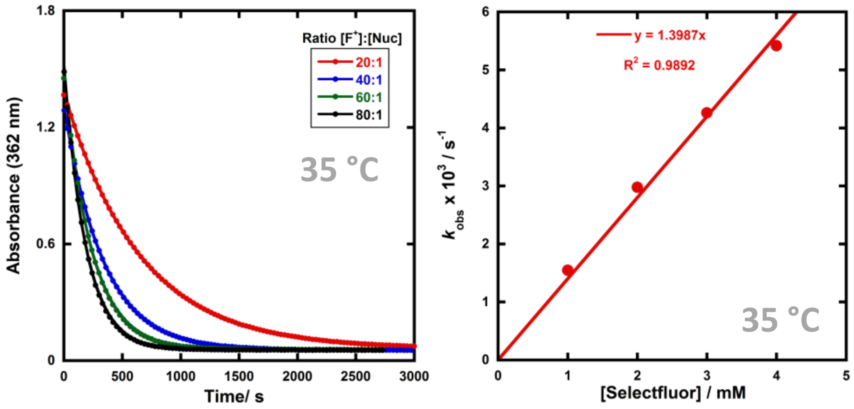

Table 20: k_{obs} values at different concentrations of Selectfluor™ at 35 °C.

Experiment	Ratio of F^+ : Nuc	$[F^+]$: [Nuc]/ mM	$k_{obs} \times 10^3$ / s^{-1}
**1**	20:1	1.0 : 0.05	1.547 ± 0.001
**2**	40:1	2.0 : 0.05	2.977 ± 0.004
**3**	60:1	3.0 : 0.05	4.26 ± 0.01
**4**	80:1	4.0 : 0.05	5.42 ± 0.02

5.4.5 Nucleophile **1e**

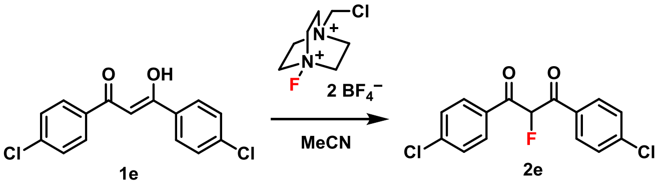

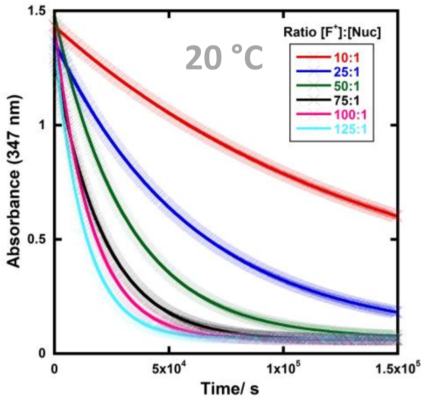

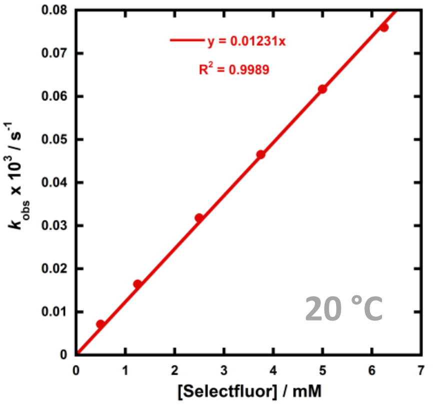

Table 21: k_{obs} values at different concentrations of Selectfluor™ at 20 °C.

Experiment	Ratio of F^+ : Nuc	$[F^+]$: [Nuc]/ mM	$k_{obs} \times 10^5$ / s^{-1}
**1**	10:1	0.5 : 0.05	0.710 ± 0.001
**2**	25:1	1.25 : 0.05	1.642 ± 0.001
**3**	50:1	2.5 : 0.05	3.177 ± 0.001
**4**	75:1	3.75 : 0.05	4.647 ± 0.001
**5**	100:1	5.0 : 0.05	6.169 ± 0.002
**6**	125:1	6.25 : 0.05	7.600 ± 0.003

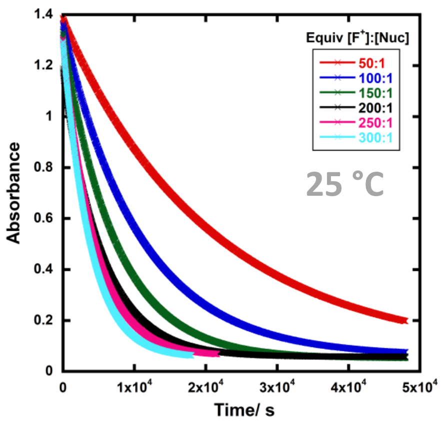

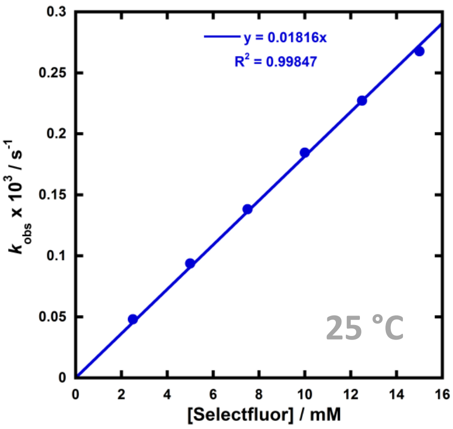

Table 22: k_{obs} values at different concentrations of Selectfluor™ at 25 °C.

Experiment	Ratio of F^+ : Nuc	$[F^+]$: [Nuc]/ mM	$k_{obs} \times 10^4$ / s^{-1}
**1**	50:1	2.50 : 0.05	0.48142 ± 0.00005
**2**	100:1	5.00 : 0.05	0.93811 ± 0.00007
**3**	150:1	7.50 : 0.05	1.3824 ± 0.0001
**4**	200:1	10.0 : 0.05	1.8462 ± 0.0002
**5**	250:1	12.5 : 0.05	2.2726 ± 0.0002
**6**	300:1	15.0 : 0.05	2.6767 ± 0.0007

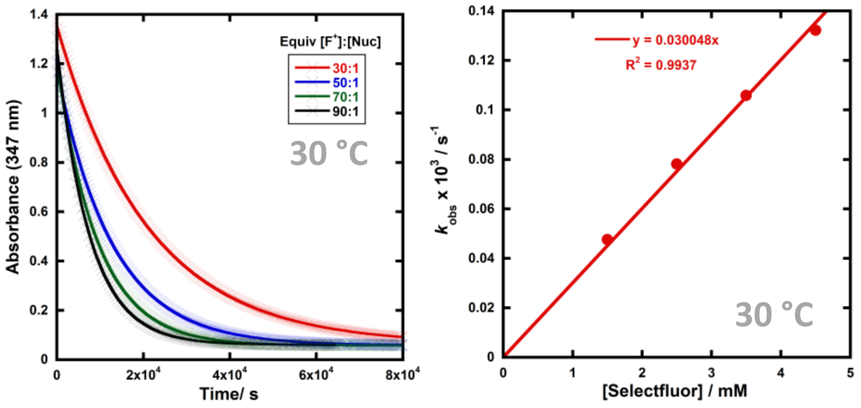

Table 23: k_{obs} values at different concentrations of Selectfluor™ at 30 °C.

Experiment	Ratio of F^+ : Nuc	$[F^+]$: [Nuc]/ mM	$k_{obs} \times 10^4$ / s^{-1}
**1**	30:1	1.5 : 0.05	0.4755 ± 0.0007
**2**	50:1	2.5 : 0.05	0.781 ± 0.001
**3**	70:1	3.5 : 0.05	1.058 ± 0.002
**4**	90:1	4.5 : 0.05	1.322 ± 0.003

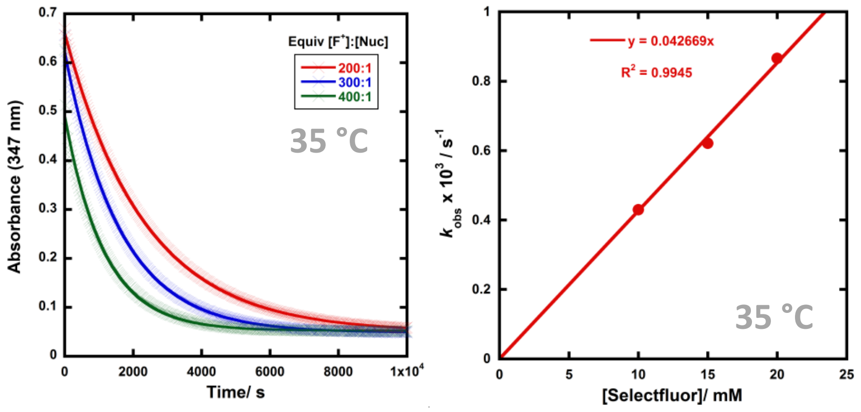

Table 24: k_{obs} values at different concentrations of Selectfluor™ at 35 °C.

Experiment	Ratio of F^+ : Nuc	[F^+] : [Nuc]/ mM	$k_{obs} \times 10^4$ / s^{-1}
**1**	200:1	10 : 0.05	4.297 ± 0.004
**2**	300:1	15 : 0.05	6.21 ± 0.01
**3**	400:1	20 : 0.05	8.66 ± 0.09

5.4.6 Nucleophile **1f**

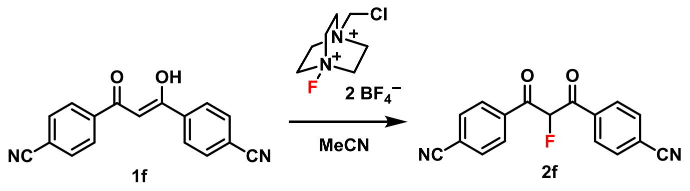

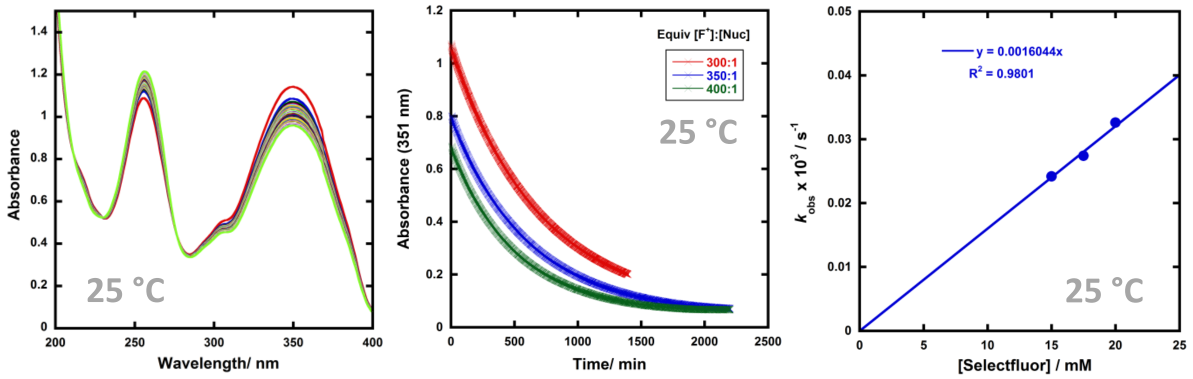

Table 25: k_{obs} values at different concentrations of Selectfluor™ at 25 °C.

Experiment	Ratio of F^+ : Nuc	[F^+] : [Nuc]/ mM	$k_{obs} \times 10^5$ / s^{-1}
**1**	300:1	15.0 : 0.05	2.417 ± 0.001
**2**	350:1	17.5 : 0.05	2.741 ± 0.001
**3**	400:1	20.0 : 0.05	3.260 ± 0.002

5.4.7 Nucleophile **1g**

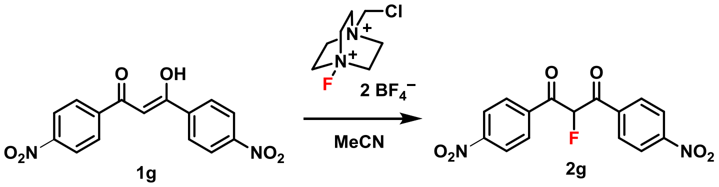

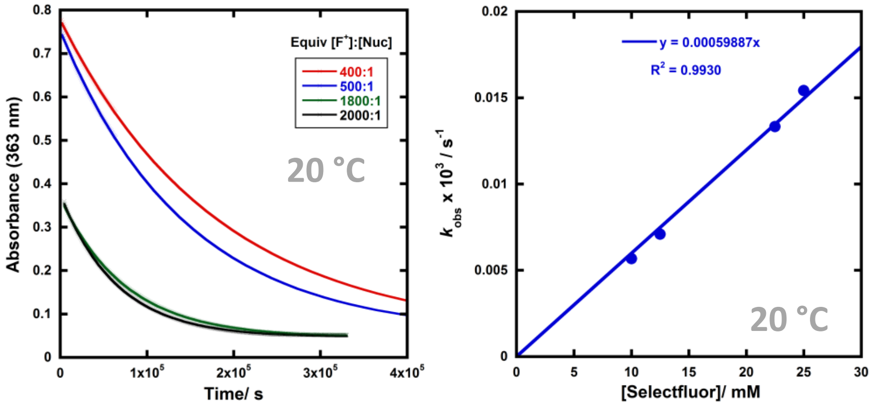

Table 26: k_{obs} values at different concentrations of Selectfluor™ at 20 °C.

Experiment	Ratio of F^+ : Nuc	$[F^+]$: [Nuc]/ mM	$k_{obs} \times 10^4$ / s^{-1}
**1**	400:1	10.0 : 0.025	0.0568 ± 0.0004
**2**	500:1	12.5 : 0.025	0.0710 ± 0.0006
**3**	1800:1	22.5 : 0.0125	0.1333 ± 0.0002
**4**	2000:1	25.0 : 0.0125	0.1542 ± 0.0003

5.4.8 Nucleophile **1h**

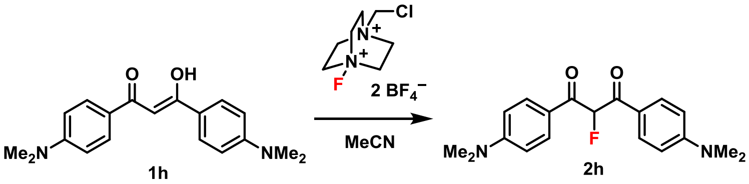

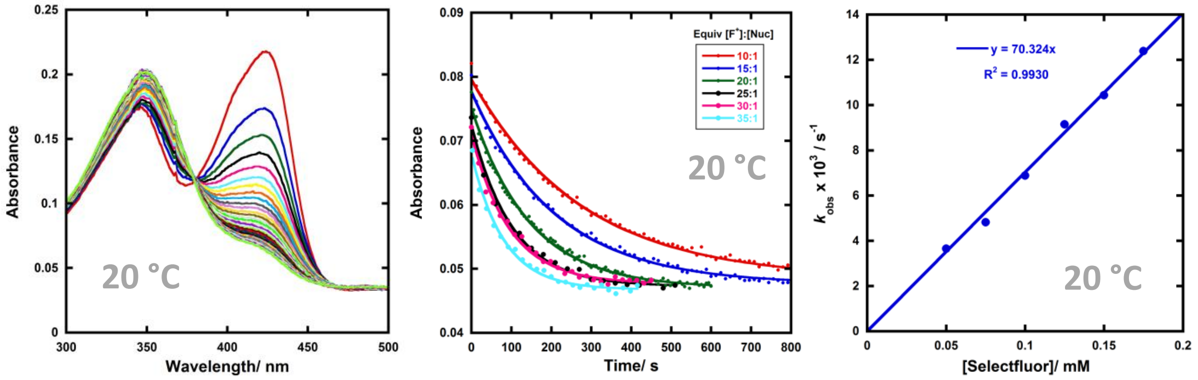

Table 27: k_{obs} values at different concentrations of Selectfluor™ at 20 °C.

Experiment	Ratio of F$^+$: Nuc	[F$^+$] : [Nuc]/ mM	$k_{obs} \times 10^3$ / s^{-1}
**1**	10:1	0.05 : 0.005	3.65 ± 0.05
**2**	15:1	0.075 : 0.005	4.82 ± 0.07
**3**	20:1	0.10 : 0.005	6.9 ± 0.1
**4**	25:1	0.125 : 0.005	9.8 ± 0.6
**5**	30:1	0.15 : 0.005	10.4 ± 0.5
**6**	35:1	0.175 : 0.005	12.4 ± 0.6

5.4.9 Nucleophile **1i**

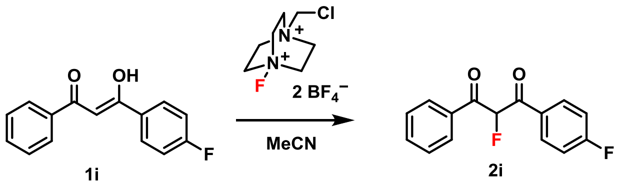

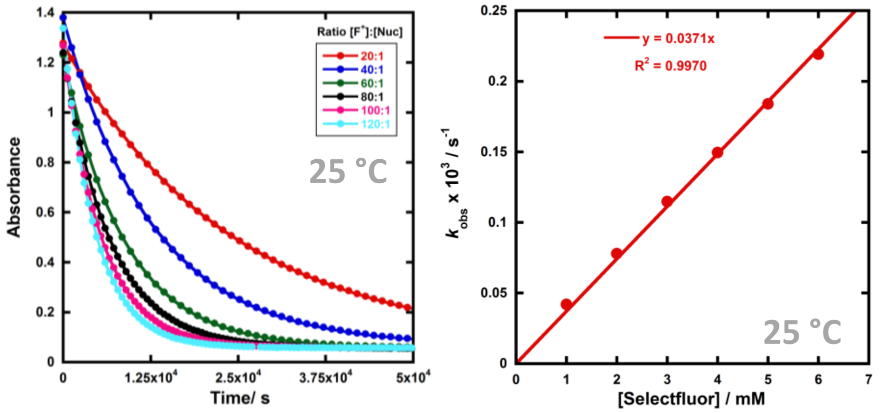

Table 28: k_{obs} values at different concentrations of Selectfluor™ at 25 °C.

Experiment	Ratio of F$^+$: Nuc	[F$^+$] : [Nuc]/ mM	$k_{obs} \times 10^4$ / s^{-1}
**1**	20:1	1.0 : 0.05	0.4197 ± 0.0004
**2**	40:1	2.0 : 0.05	0.780 ± 0.001
**3**	60:1	3.0 : 0.05	1.148 ± 0.001
**4**	80:1	4.0 : 0.05	1.495 ± 0.001
**5**	100:1	5.0 : 0.05	1.840 ± 0.001
**6**	120:1	6.0 : 0.05	2.193 ± 0.002

5.4.10 Nucleophile **1j**

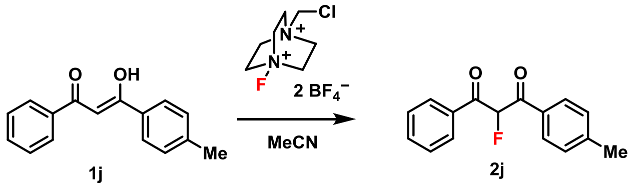

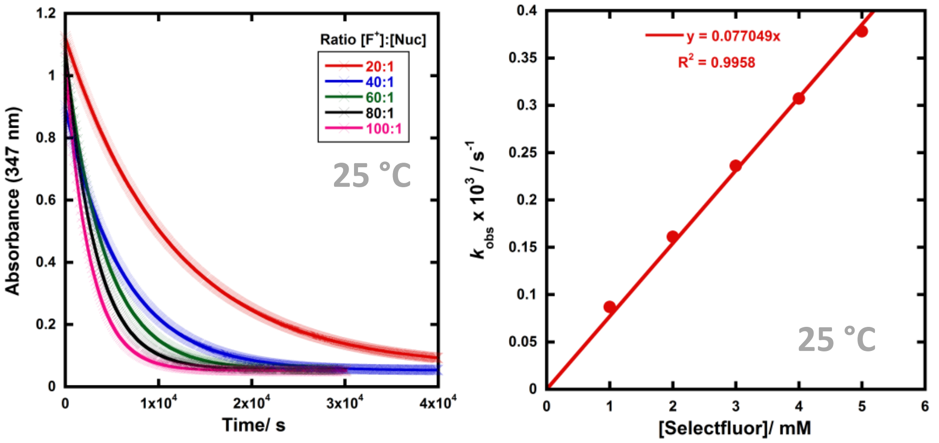

Table 29: k_{obs} values at different concentrations of Selectfluor™ at 25 °C.

Experiment	Ratio of F^+ : Nuc	$[F^+]$: [Nuc]/ mM	$k_{obs} \times 10^4$ / s^{-1}
**1**	20:1	1.0 : 0.05	0.8692 ± 0.0004
**2**	40:1	2.0 : 0.05	1.6115 ± 0.0008
**3**	60:1	3.0 : 0.05	2.361 ± 0.002
**4**	80:1	4.0 : 0.05	3.073 ± 0.004
**5**	100:1	5.0 : 0.05	3.783 ± 0.009

5.4.11 Nucleophile **1k**

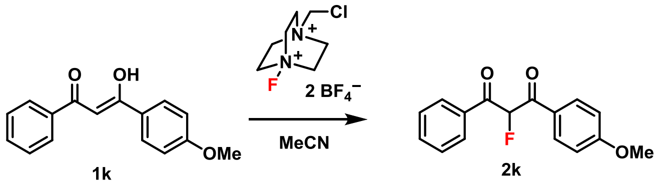

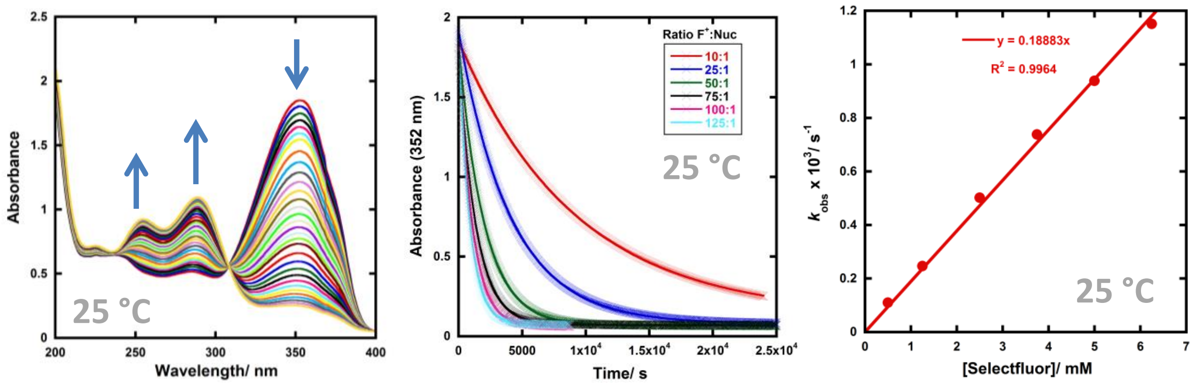

Table 30: k_{obs} **values at different concentrations of Selectfluor™ at 25 °C.**

Experiment	Ratio of F^+ : Nuc	$[F^+]$: [Nuc]/ mM	$k_{obs} \times 10^3$ / s^{-1}
**1**	10:1	0.50 : 0.05	0.1096 ± 0.0001
**2**	25:1	1.25 : 0.05	0.2463 ± 0.0001
**3**	50:1	2.50 : 0.05	0.5017 ± 0.0002
**4**	75:1	3.75 : 0.05	0.7380 ± 0.0003
**5**	100:1	5.00 : 0.05	0.9386 ± 0.0003
**6**	125:1	6.25 : 0.05	1.1515 ± 0.0004

5.4.12 Nucleophile **1l**

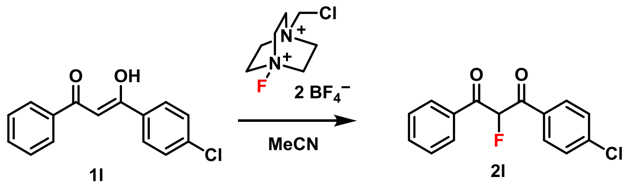

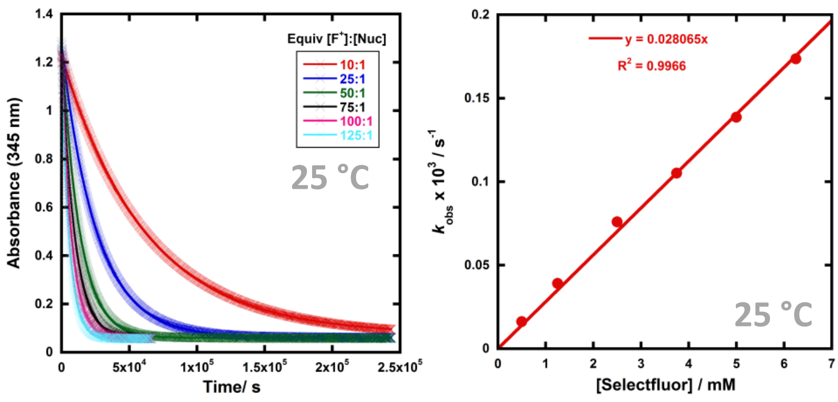

Table 31: k_{obs} values at different concentrations of Selectfluor™ at 25 °C.

Experiment	Ratio of F^+ : Nuc	$[F^+]$: [Nuc]/ mM	$k_{obs} \times 10^5$ / s^{-1}
**1**	10:1	0.50 : 0.05	1.6224 ± 0.0007
**2**	25:1	1.25 : 0.05	3.9121 ± 0.0009
**3**	50:1	2.50 : 0.05	7.594 ± 0.002
**4**	75:1	3.75 : 0.05	10.515 ± 0.003
**5**	100:1	5.00 : 0.05	13.858 ± 0.005
**6**	125:1	6.25 : 0.05	17.356 ± 0.006

5.4.13 Nucleophile **1m**

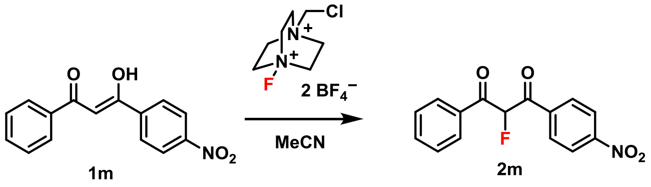

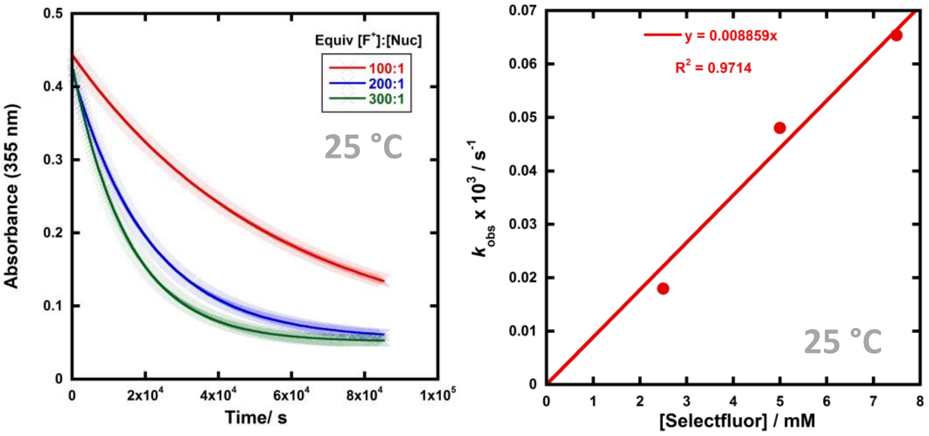

Table 32: k_{obs} values at different concentrations of Selectfluor™ at 25 °C.

Experiment	Ratio of F^+ : Nuc	$[F^+]$: [Nuc]/ mM	$k_{obs} \times 10^4$ / s^{-1}
**1**	100:1	2.5 : 0.025	0.1793 ± 0.0002
**2**	200:1	5.0 : 0.025	0.4803 ± 0.0006
**3**	300:1	7.5 : 0.025	0.654 ± 0.001

5.5 Kinetics Reactions Involving NFSI (4)

5.5.1 Nucleophile **1a**

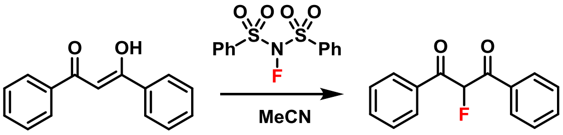

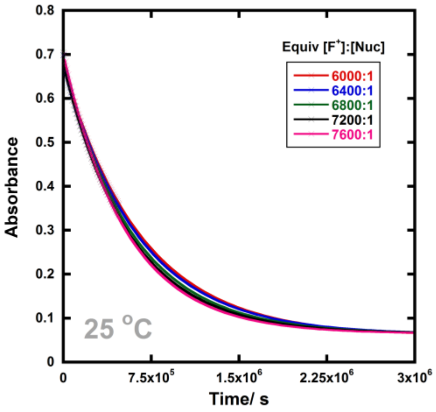

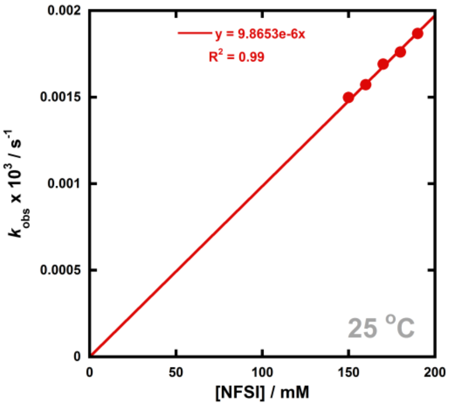

Table 33: k_{obs} values at different concentrations of NFSI at 25 °C.

Experiment	Ratio of F^+ : Nuc	$[F^+]$: [Nuc]/ mM	$k_{obs} \times 10^5$ / s^{-1}
**1**	6000:1	150 : 0.025	0.1498 ± 0.0007
**2**	6400:1	160 : 0.025	0.157 ± 0.002
**3**	6800:1	170 : 0.025	0.169 ± 0.003
**4**	7200:1	180 : 0.025	0.176 ± 0.003
**5**	7600:1	190 : 0.025	0.187 ± 0.002

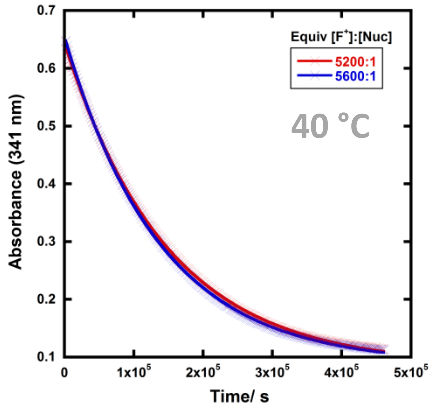

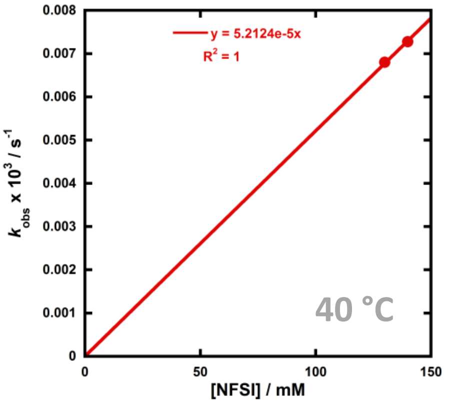

Table 34: k_{obs} values at different concentrations of NFSI at 40 °C.

Experiment	Ratio of F^+ : Nuc	$[F^+]$: [Nuc]/ mM	$k_{obs} \times 10^4$ / s^{-1}
**1**	5200:1	130 : 0.025	0.0680 ± 0.0002
**2**	5600:1	140 : 0.025	0.0728 ± 0.0002

5.5.2 Nucleophile **1b**

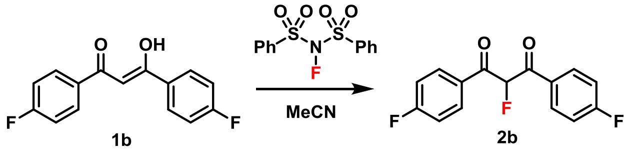

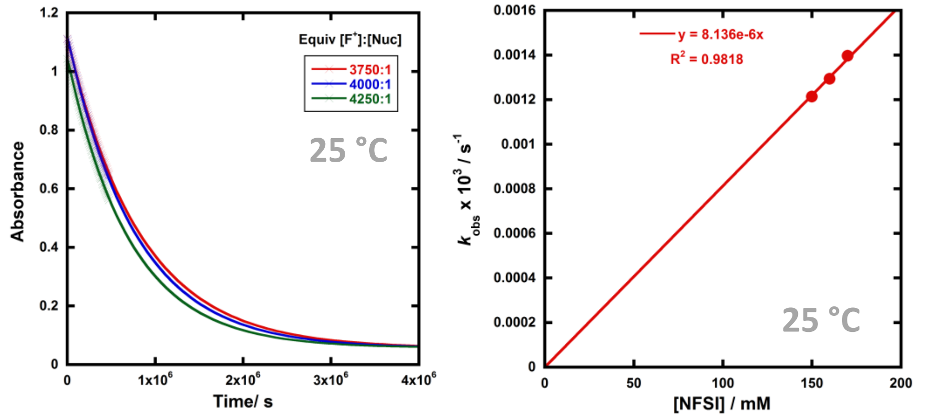

Table 35: k_{obs} values at different concentrations of NFSI at 25 °C.

Experiment	Ratio of F^+ : Nuc	$[F^+]$: [Nuc]/ mM	$k_{obs} \times 10^5$ / s^{-1}
**1**	3750:1	150 : 0.04	0.1214 ± 0.0004
**2**	4000:1	160 : 0.04	0.1293 ± 0.0005
**3**	4250:1	170 : 0.05	0.1397 ± 0.0006

5.5.3 Nucleophile **1c**

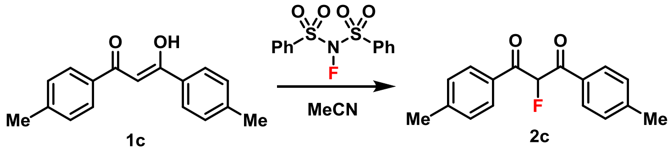

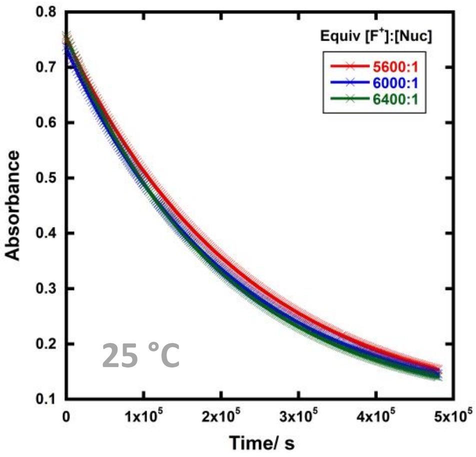

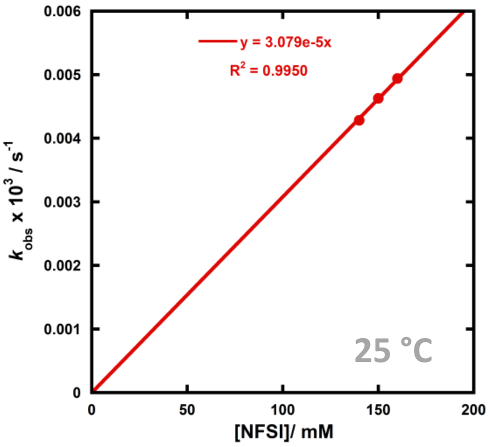

Table 36: k_{obs} values at different concentrations of NFSI at 25 °C.

Experiment	Ratio of F^+ : Nuc	$[F^+]$: [Nuc]/ mM	$k_{obs} \times 10^5$ / s^{-1}
**1**	5600:1	140 : 0.025	0.4283 ± 0.0004
**2**	6000:1	150 : 0.025	0.4629 ± 0.0004
**3**	6400:1	160 : 0.025	0.4941 ± 0.0004

5.5.4 Nucleophile **1d**

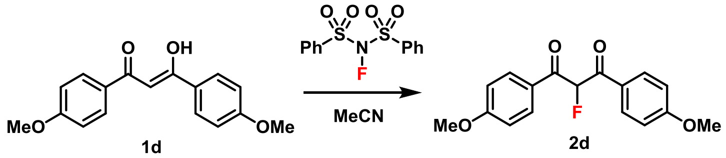

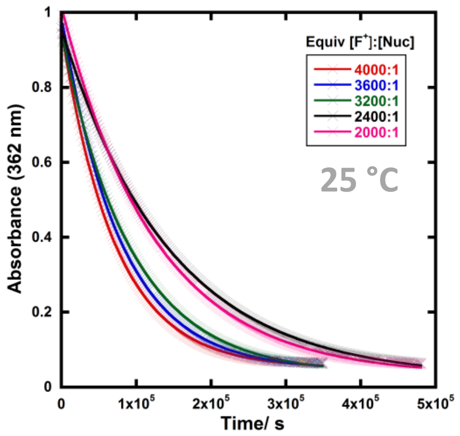

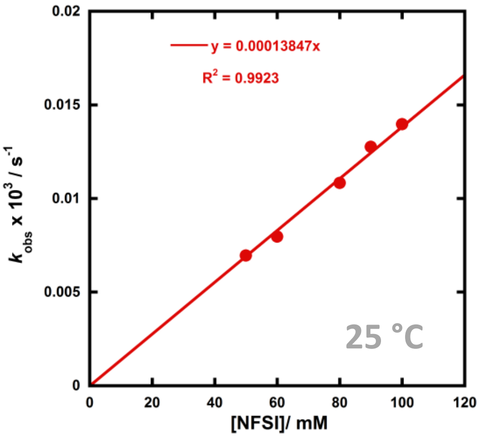

Table 37: k_{obs} values at different concentrations of NFSI at 25 °C.

Experiment	Ratio of F^+ : Nuc	$[F^+]$: [Nuc]/ mM	k_{obs} x 10^5 / s^{-1}
**1**	2000:1	50 : 0.025	0.695 ± 0.001
**2**	2400:1	60 : 0.025	0.797 ± 0.002
**3**	3200:1	80 : 0.025	1.083 ± 0.002
**4**	3600:1	90 : 0.025	1.276 ± 0.003
**5**	4000:1	100 : 0.025	1.397 ± 0.004

5.5.5 Nucleophile **1e**

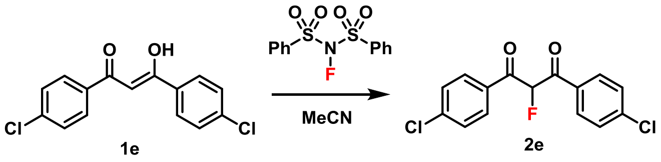

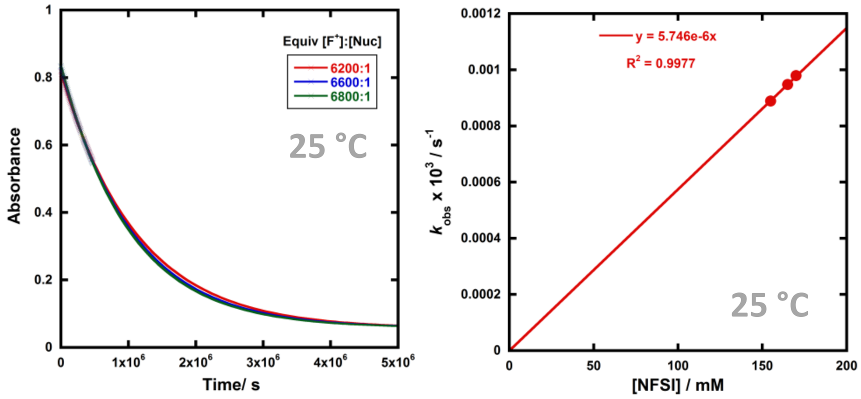

Table 38: k_{obs} values at different concentrations of NFSI at 25 °C.

Experiment	Ratio of F^+ : Nuc	$[F^+]$: [Nuc]/ mM	k_{obs} x 10^5 / s^{-1}
**1**	6200:1	155 : 0.025	0.0889 ± 0.0001
**2**	6600:1	165 : 0.025	0.0947 ± 0.0001
**3**	6800:1	170 : 0.025	0.0979 ± 0.0001

5.5.6 Nucleophile **1h**

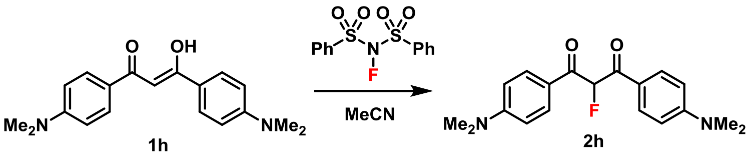

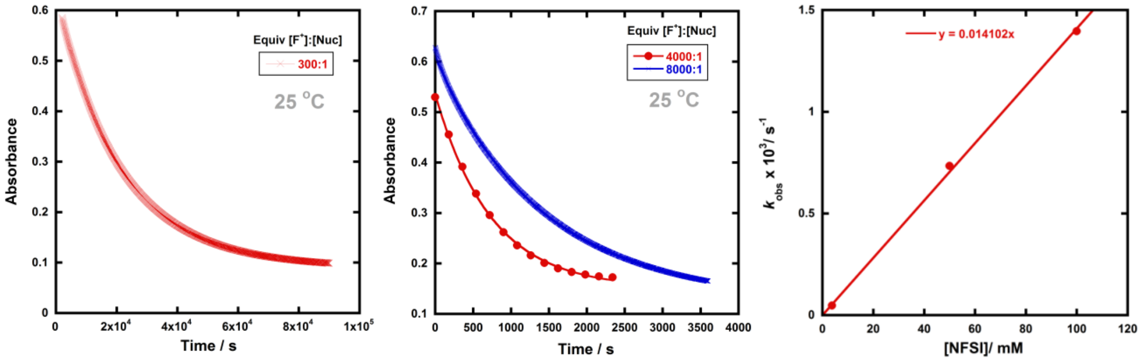

Table 39: k_{obs} values at different concentrations of NFSI at 25 °C.

Experiment	Ratio of F$^+$: Nuc	[F$^+$] : [Nuc]/ mM	k_{obs} x 10^5 / s^{-1}
**1**	300:1	3.75 : 0.025	0.04757 ± 0.00001
**2**	4000:1	50 : 0.0125	0.7339 ± 0.0009
**3**	8000:1	100 : 0.0125	1.36 ± 0.04

5.5.7 Nucleophile **1j**

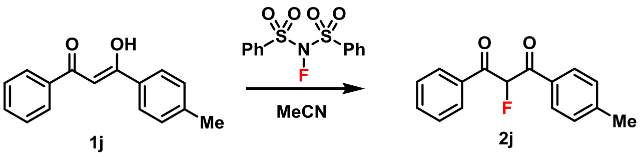

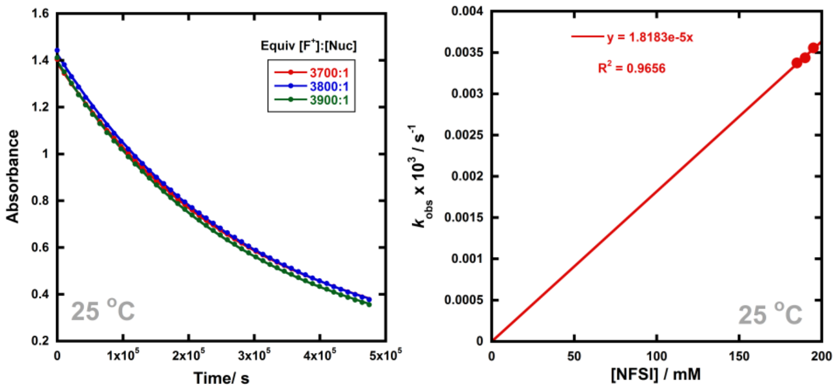

Table 40: k_{obs} values at different concentrations of NFSI at 25 °C.

Experiment	Ratio of F$^+$: Nuc	[F$^+$] : [Nuc]/ mM	k_{obs} x 10^4 / s^{-1}
**1**	3700:1	185 : 0.05	0.0337 ± 0.0003
**2**	3800:1	190 : 0.05	0.0344 ± 0.0003
**3**	3900:1	195 : 0.05	0.0356 ± 0.0003

5.5.8 Nucleophile **1k**

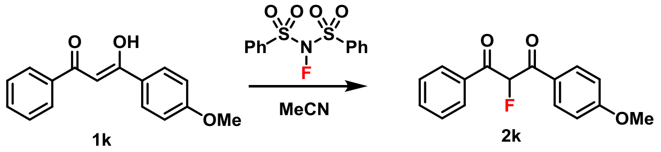

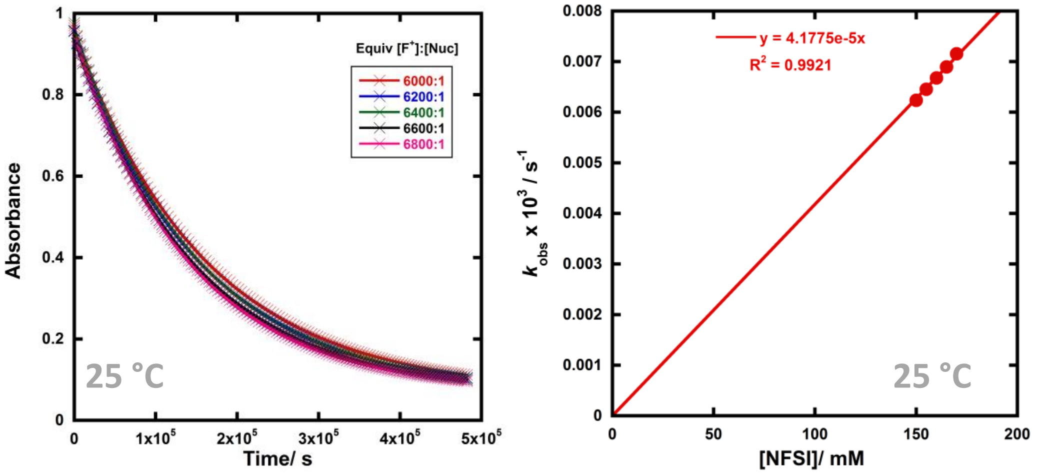

Table 41: k_{obs} values at different concentrations of NFSI at 25 °C.

Experiment	Ratio of F^+ : Nuc	$[F^+]$: [Nuc]/ mM	$k_{obs} \times 10^4$ / s^{-1}
**1**	6000:1	150 : 0.025	0.0624 ± 0.0001
**2**	6200:1	155 : 0.025	0.0645 ± 0.0002
**3**	6400:1	160 : 0.025	0.0668 ± 0.0001
**4**	6600:1	165 : 0.025	0.0689 ± 0.0002
**5**	6800:1	170 : 0.025	0.0715 ± 0.0002

5.6 Kinetics Reactions Involving Synfluor™ (5)

5.6.1 Nucleophile **1d**

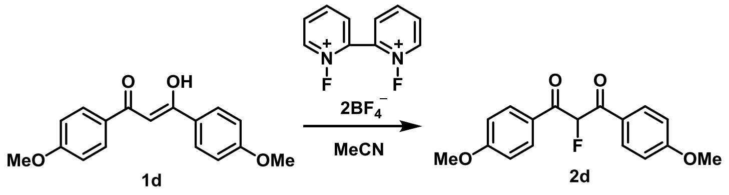

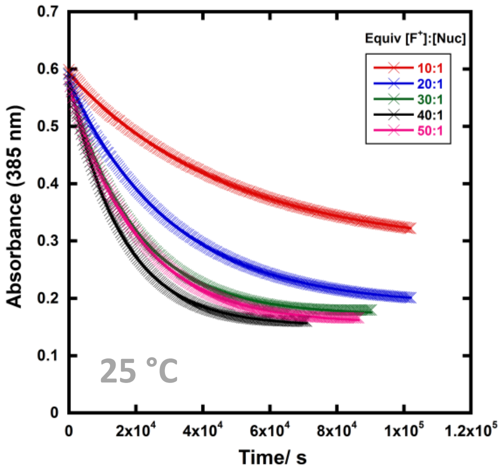

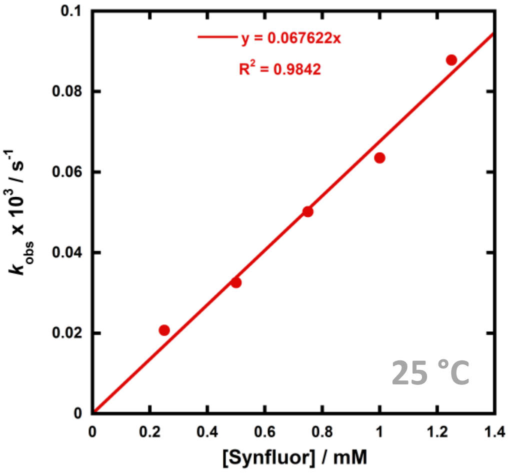

Table 42: k_{obs} values at different concentrations of Synfluor at 25 °C.

Experiment	Ratio of F^+ : Nuc	$[F^+]$: [Nuc]/ mM	$k_{obs} \times 10^4$ / s^{-1}
**1**	10:1	0.5 : 0.05	0.2070 ± 0.0009
**2**	20:1	1.0 : 0.05	0.3256 ± 0.0009
**3**	30:1	1.5 : 0.05	0.502 ± 0.002
**4**	40:1	2.0 : 0.05	0.636 ± 0.002
**5**	50:1	2.5 : 0.05	0.878 ± 0.002

5.6.2 Nucleophile **1k**

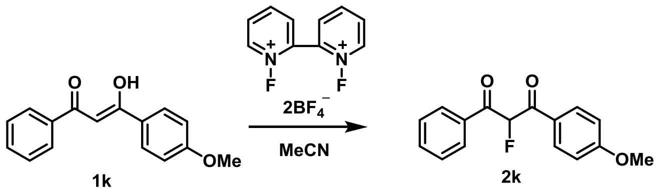

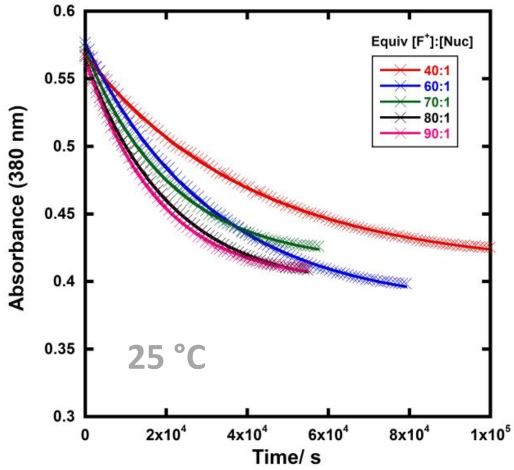

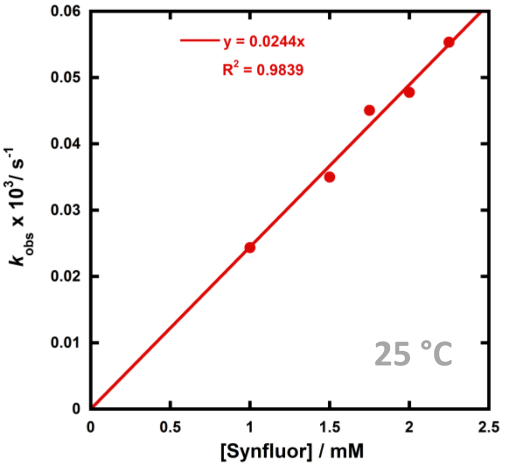

Table 43: k_{obs} values at different concentrations of Synfluor at 25 °C.

Experiment	Ratio of F^+ : Nuc	$[F^+]$: [Nuc]/ mM	$k_{obs} \times 10^3$ / s^{-1}
**1**	40:1	2.0 : 0.05	0.0244 ± 0.0002
**2**	60:1	3.0 : 0.05	0.0350 ± 0.0002
**3**	70:1	3.5 : 0.05	0.0451 ± 0.0006
**4**	80:1	4.0 : 0.05	0.0478 ± 0.0005
**5**	90:1	4.5 : 0.05	0.0553 ± 0.0007

5.7 Kinetics Reactions Involving 2,6-dichloro-*N*-fluoropyridinium triflate (8a)

5.7.1 Nucleophile **1a**

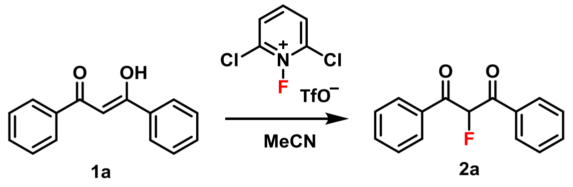

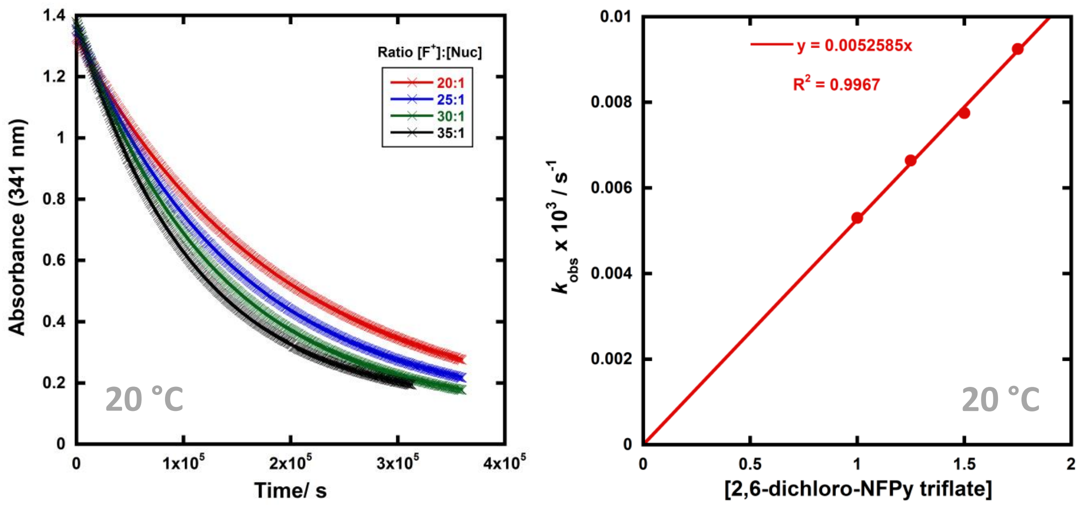

Table 44: k_{obs} values at different concentrations of 8a at 20 °C.

Experiment	Ratio of F^+ : Nuc	$[F^+]$: [Nuc]/ mM	$k_{obs} \times 10^5$ / s^{-1}
**1**	20:1	1.0 : 0.05	0.5301 ± 0.0005
**2**	25:1	1.25 : 0.05	0.6640 ± 0.0005
**3**	30:1	1.5 : 0.05	0.7748 ± 0.0006
**4**	35:1	1.75 : 0.05	0.9116 ± 0.0007

5.7.2 Nucleophile **1b**

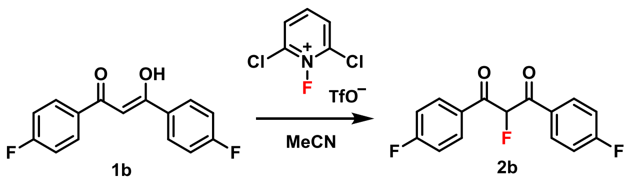

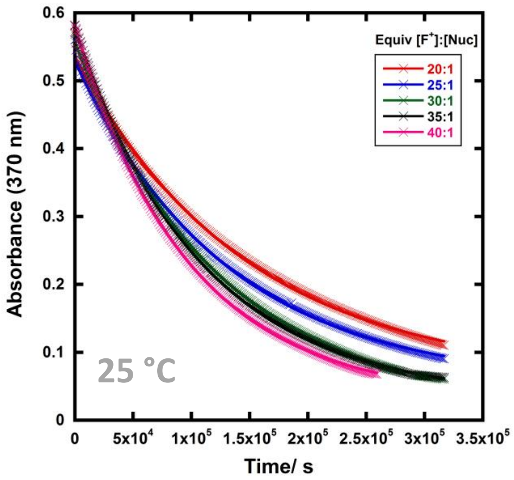

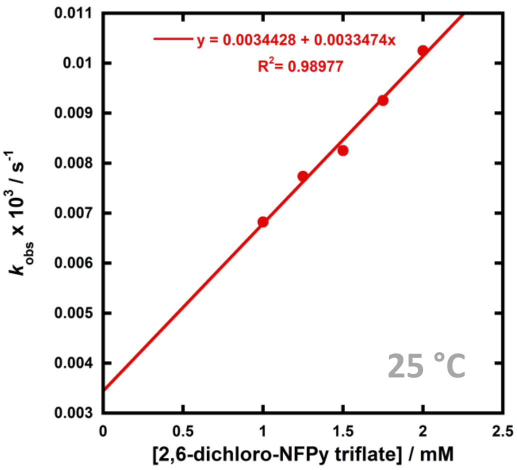

Table 45: k_{obs} values at different concentrations of 8a at 25 °C.

Experiment	Ratio of F^+ : Nuc	$[F^+]$: [Nuc]/ mM	$k_{obs} \times 10^5$ / s^{-1}
**1**	20:1	1.0 : 0.05	0.6826 ± 0.0006
**2**	25:1	1.25 : 0.05	0.7736 ± 0.0005
**3**	30:1	1.5 : 0.05	0.8253 ± 0.0002
**4**	35:1	1.75 : 0.05	0.9255 ± 0.0002
**5**	40:1	2.0 : 0.05	1.0250 ± 0.0003

5.7.3 Nucleophile **1c**

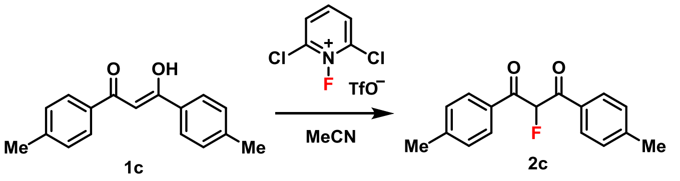

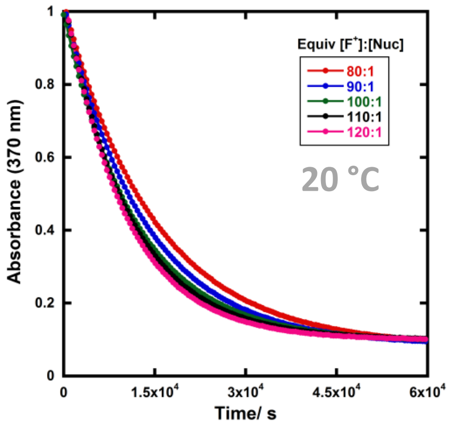

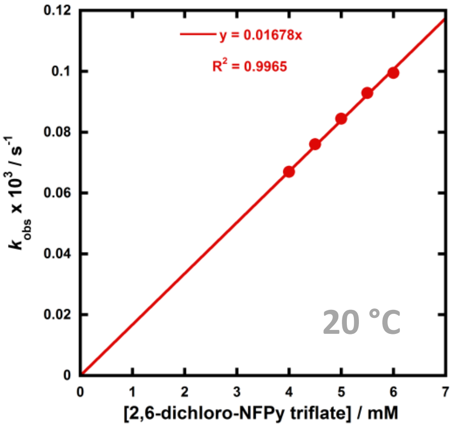

Table 46: k_{obs} values at different concentrations of 8a at 20 °C.

Experiment	Ratio of F^+ : Nuc	$[F^+]$: [Nuc]/ mM	$k_{obs} \times 10^4$ / s^{-1}
**1**	80:1	4.0 : 0.05	0.6696 ± 0.0005
**2**	90:1	4.5 : 0.05	0.7601 ± 0.0005
**3**	100:1	5.0 : 0.05	0.8444 ± 0.0006
**4**	110:1	5.5 : 0.05	0.9287 ± 0.0007
**5**	120:1	6.0 : 0.05	0.9948 ± 0.0009

5.7.4 Nucleophile **1d**

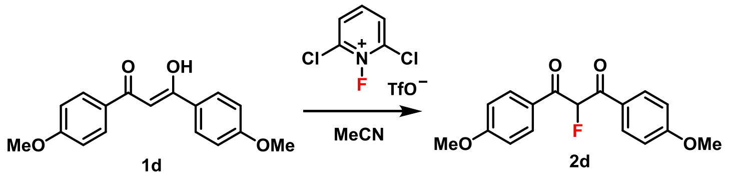

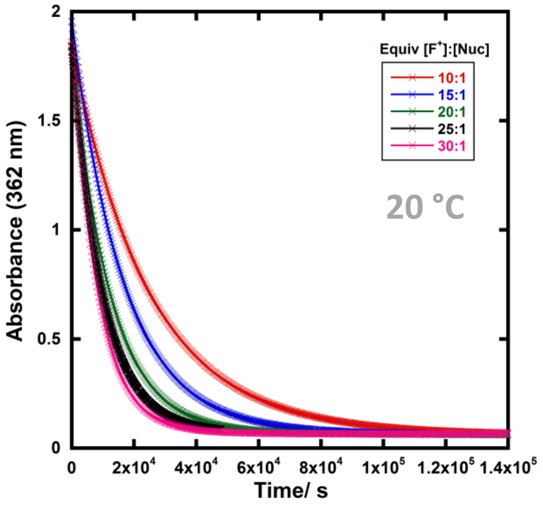

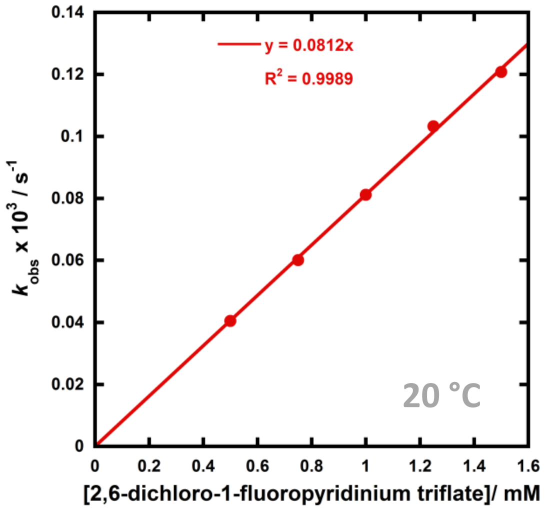

Table 47: k_{obs} values at different concentrations of 8a at 20 °C.

Experiment	Ratio of F^+ : Nuc	$[F^+]$: [Nuc]/ mM	$k_{obs} \times 10^4$ / s^{-1}
**1**	10:1	0.50 : 0.05	0.4046 ± 0.0002
**2**	15:1	0.75 : 0.05	0.6013 ± 0.0004
**3**	20:1	1.00 : 0.05	0.8115 ± 0.0005
**4**	25:1	1.25 : 0.05	1.0326 ± 0.0004
**5**	30:1	1.50 : 0.05	1.2079 ± 0.0007

5.7.5 Nucleophile **1e**

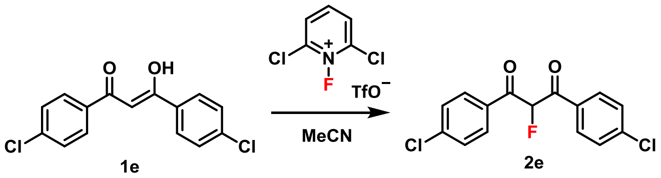

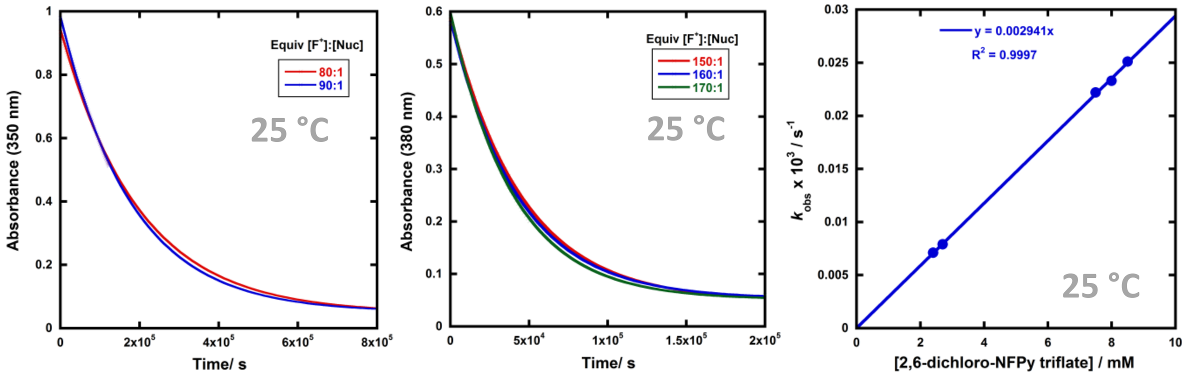

Table 48: k_{obs} values at different concentrations of 8a at 25 °C.

Experiment	Ratio of F$^+$: Nuc	[F$^+$] : [Nuc]/ mM	$k_{obs} \times 10^4$ / s^{-1}
**1**	80:1	2.4 : 0.03	0.0711 ± 0.0006
**2**	90:1	2.7 : 0.03	0.0791 ± 0.0005
**3**	150:1	7.5 : 0.05	0.222 ± 0.002
**4**	160:1	8.0 : 0.05	0.233 ± 0.001
**5**	170:1	8.5 : 0.05	0.251 ± 0.002

5.8 Kinetics Reactions Involving 2,6-dichloro-*N*-fluoropyridinium tetrafluoroborate (8b)

5.8.1 Nucleophile **1a**

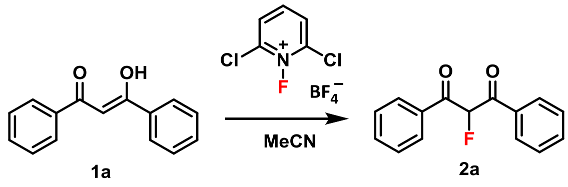

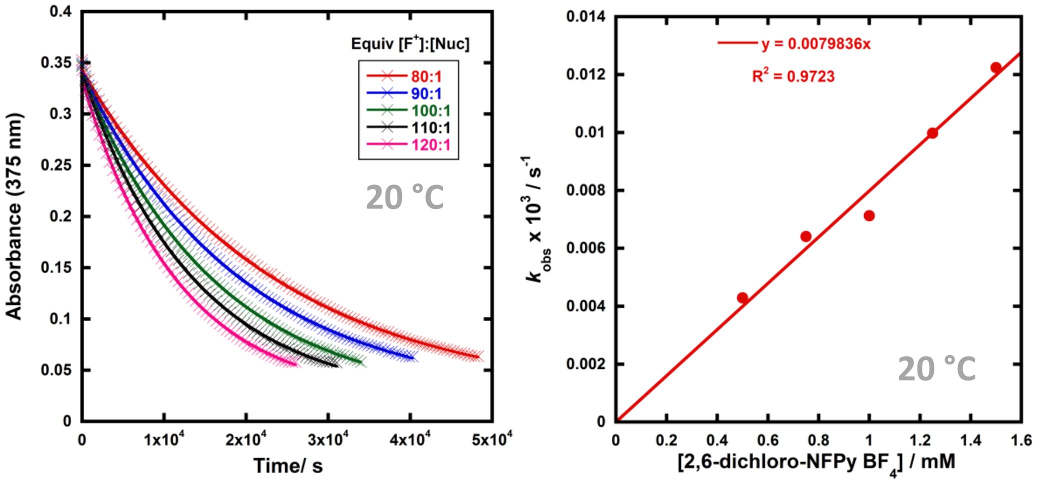

Table 49: k_{obs} values at different concentrations of 8b at 20 °C.

Experiment	Ratio of F$^+$: Nuc	[F$^+$] : [Nuc]/ mM	$k_{obs} \times 10^5$ / s^{-1}
**1**	80:1	4.0 : 0.05	0.4291 ± 0.0005
**2**	90:1	4.5 : 0.05	0.6408 ± 0.0005
**3**	100:1	5.0 : 0.05	0.7122 ± 0.0006
**4**	110:1	5.5 : 0.05	0.9979 ± 0.0007
**5**	120:1	6.0 : 0.05	1.2241 ± 0.0007

5.8.2 Nucleophile **1b**

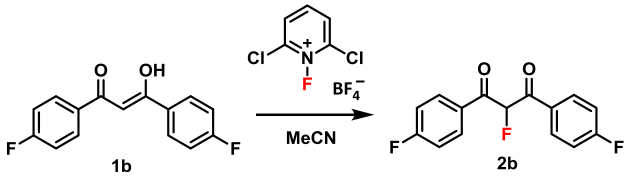

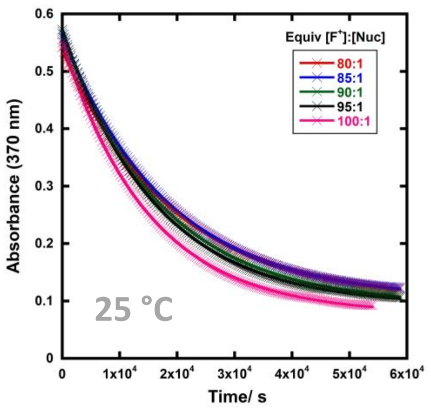

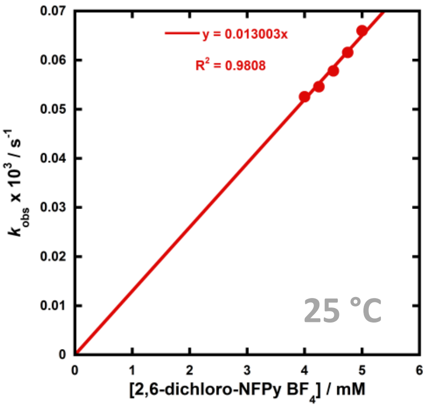

Table 50: k_{obs} values at different concentrations of 8b at 25 °C.

Experiment	Ratio of F^+ : Nuc	$[F^+]$: [Nuc]/ mM	$k_{obs} \times 10^4$ / s^{-1}
**1**	80:1	4.00 : 0.05	0.5254 ± 0.0007
**2**	85:1	4.25 : 0.05	0.5459 ± 0.0008
**3**	90:1	4.50 : 0.05	0.578 ± 0.001
**4**	95:1	4.75 : 0.05	0.616 ± 0.001
**5**	100:1	5.00 : 0.05	0.660 ± 0.002

5.8.3 Nucleophile **1c**

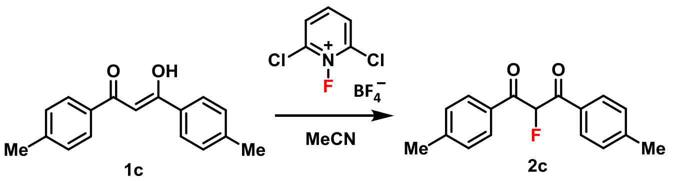

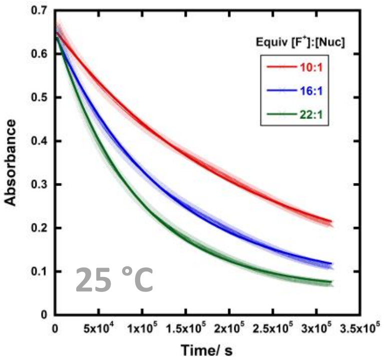

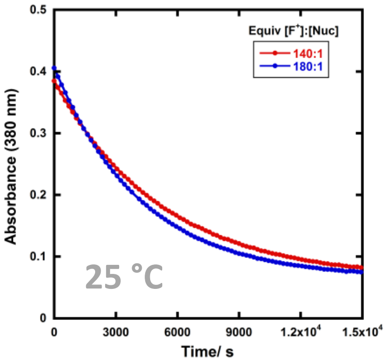

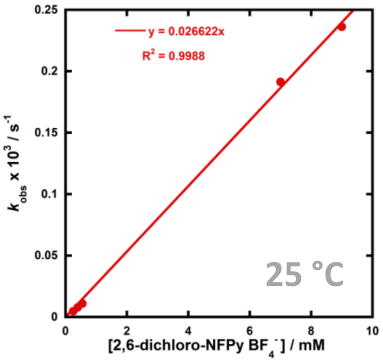

Table 51: k_{obs} values at different concentrations of 8b at 25 °C.

Experiment	Ratio of F^+ : Nuc	$[F^+]$: [Nuc]/ mM	$k_{obs} \times 10^4$ / s^{-1}
**1**	10:1	0.25 : 0.025	0.0471 ± 0.0006
**2**	16:1	0.40 : 0.025	0.0785 ± 0.0006
**3**	22:1	0.55 : 0.025	0.1093 ± 0.0005
**4**	140:1	7.00 : 0.05	1.913 ± 0.003
**5**	180:1	9.00 : 0.05	2.362 ± 0.003

5.8.4 Nucleophile **1d**

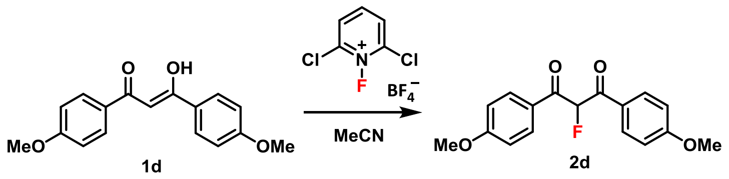

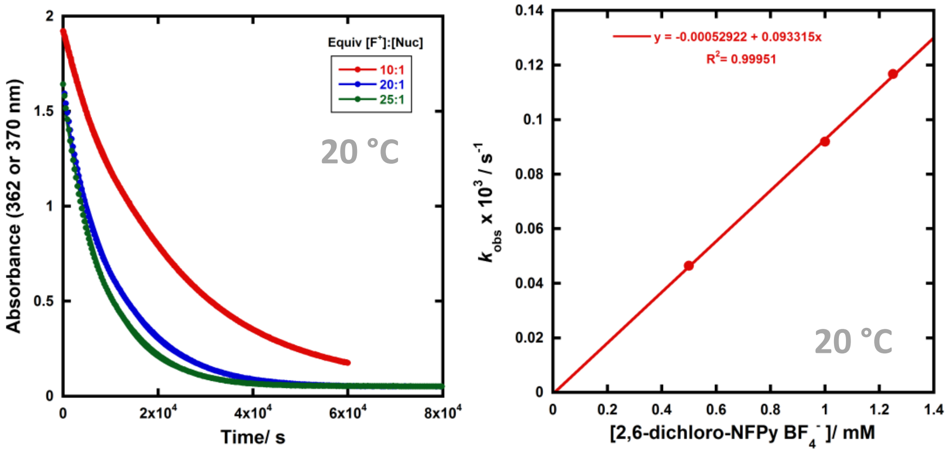

Table 52: k_{obs} values at different concentrations of 8b at 20 °C.

Experiment	Ratio of F^+ : Nuc	$[F^+]$: [Nuc]/ mM	$k_{obs} \times 10^4$ / s^{-1}
**1**	10:1	0.5 : 0.05	0.464 ± 0.001
**2**	20:1	1.0 : 0.05	0.919 ± 0.004
**3**	25:1	1.25 : 0.05	1.167 ± 0.006

5.8.5 Nucleophile **1e**

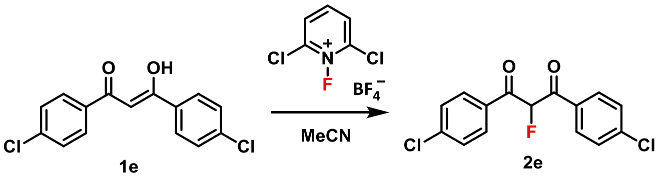

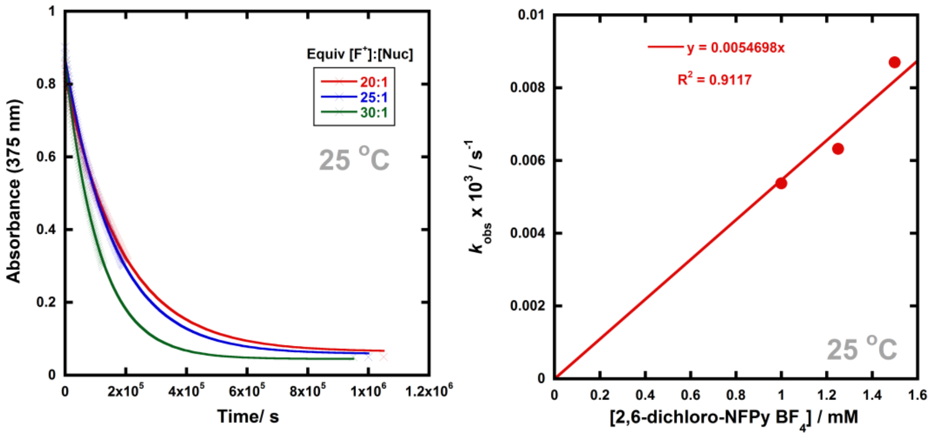

Table 53: k_{obs} values at different concentrations of 8b at 25 °C.

Experiment	Ratio of F^+ : Nuc	$[F^+]$: [Nuc]/ mM	$k_{obs} \times 10^5$ / s^{-1}
**1**	20:1	1.0 : 0.05	0.5370 ± 0.0009
**2**	25:1	1.25 : 0.05	0.6323 ± 0.0008
**3**	30:1	1.5 : 0.05	8.700 ± 0.001

5.8.6 Nucleophile **1j**

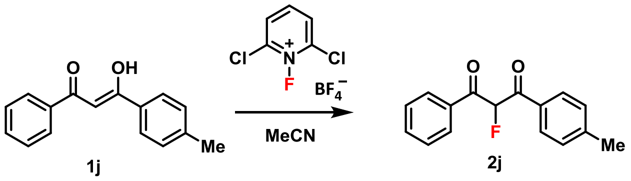

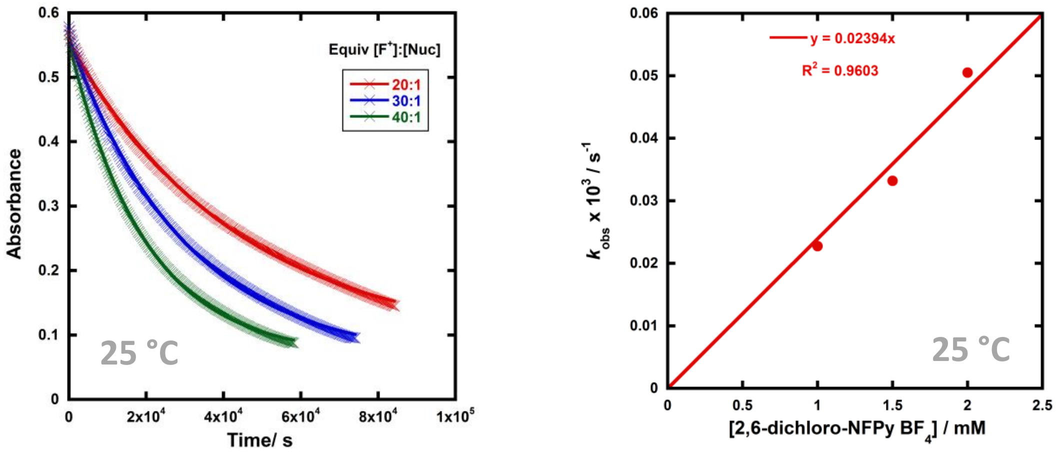

Table 54: k_{obs} values at different concentrations of 8b at 25 °C.

Experiment	Ratio of F^+ : Nuc	$[F^+]$: [Nuc]/ mM	$k_{obs} \times 10^4$ / s^{-1}
**1**	20:1	1.0 : 0.05	0.228 ± 0.001
**2**	30:1	1.5 : 0.05	0.332 ± 0.003
**3**	40:1	2.0 : 0.05	0.505 ± 0.003

5.8.7 Nucleophile **1k**

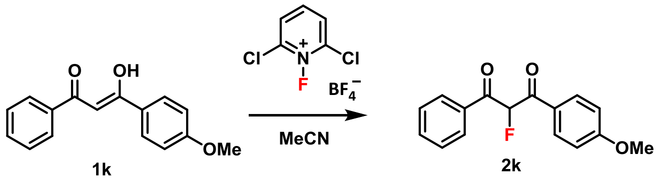

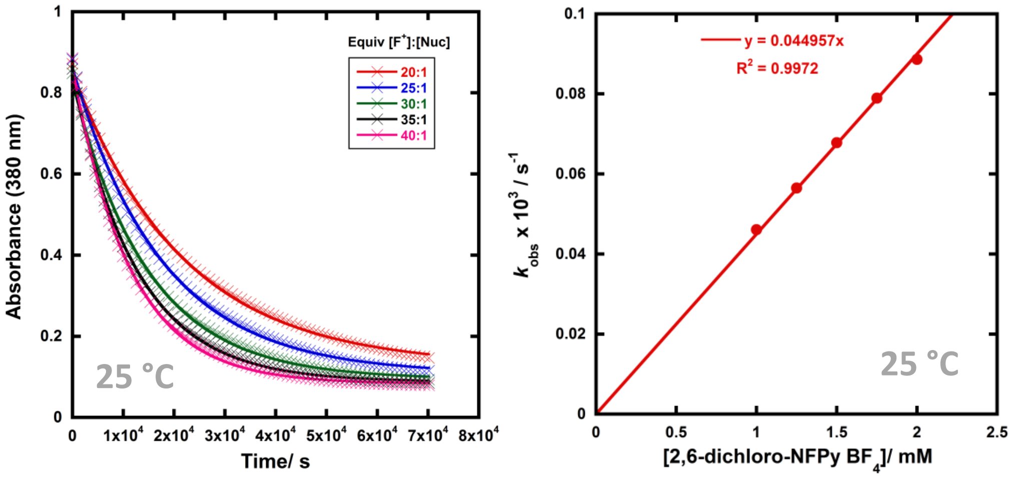

Table 55: k_{obs} values at different concentrations of 8b at 25 °C.

Experiment	Ratio of F^+ : Nuc	$[F^+]$: [Nuc]/ mM	$k_{obs} \times 10^4$ / s^{-1}
**1**	20:1	1.0 : 0.05	0.461 ± 0.005
**2**	25:1	1.25 : 0.05	0.565 ± 0.006
**3**	30:1	1.5 : 0.05	0.678 ± 0.007
**4**	35:1	1.75 : 0.05	0.790 ± 0.007
**5**	40:1	2.0 : 0.05	0.886 ± 0.009

5.9 Kinetics Reactions Involving 2,3,4,5,6-pentachloro-N-fluoropyridinium triflate (9)

5.9.1 Nucleophile **1a**

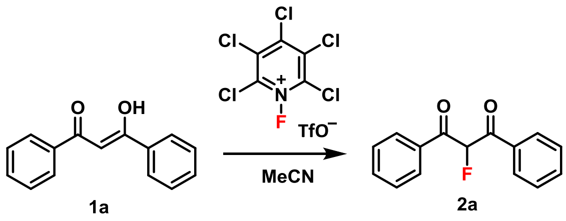

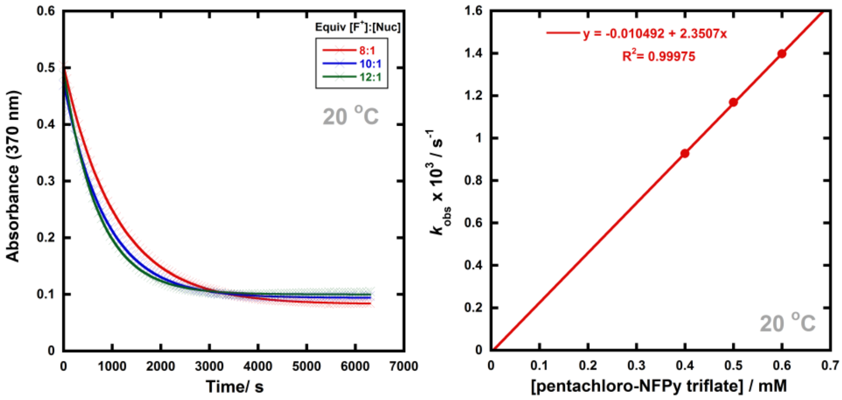

Table 56: k_{obs} values at different concentrations of 9 at 20 °C.

Experiment	Ratio of F^+ : Nuc	$[F^+]$: [Nuc]/ mM	$k_{obs} \times 10^3$ / s^{-1}
**1**	8:1	0.4 : 0.05	0.928 ± 0.001
**2**	10:1	0.5 : 0.05	1.169 ± 0.004
**3**	12:1	0.6 : 0.05	1.398 ± 0.008

5.9.2 Nucleophile **1c**

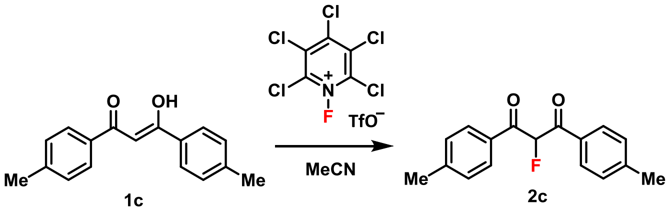

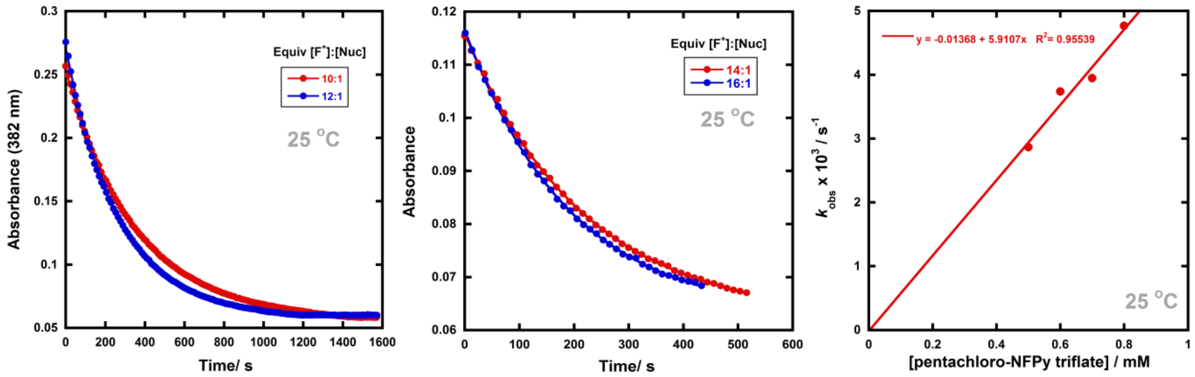

Table 57: k_{obs} values at different concentrations of 9 at 25 °C.

Experiment	Ratio of F^+ : Nuc	$[F^+]$: [Nuc]/ mM	$k_{obs} \times 10^3$ / s^{-1}
**1**	10:1	0.5 : 0.05	2.8651 ± 0.01
**2**	12:1	0.6 : 0.05	3.7365 ± 0.04
**3**	14:1	0.7 : 0.05	3.9460 ± 0.02
**4**	16:1	0.8 : 0.05	4.7655 ± 0.02

5.9.3 Nucleophile **1d**

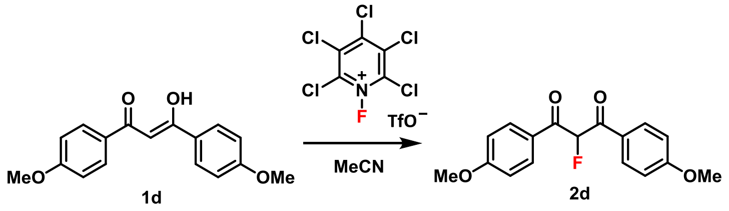

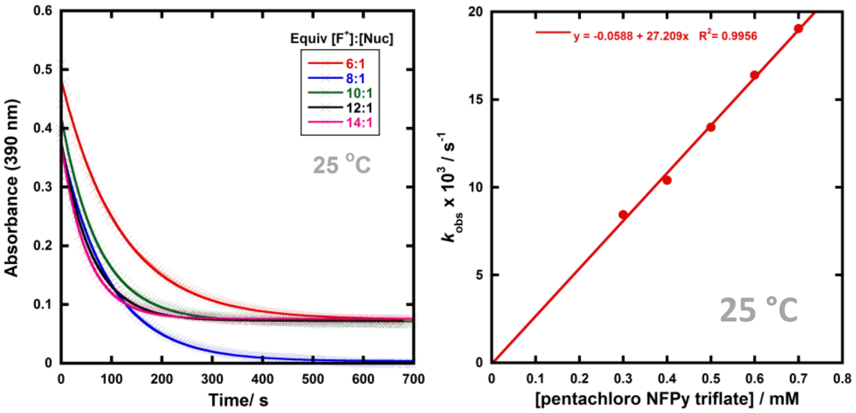

Table 58: k_{obs} values at different concentrations of 9 at 25 °C.

Experiment	Ratio of F^+ : Nuc	$[F^+]$: [Nuc]/ mM	$k_{obs} \times 10^3$ / s^{-1}
**1**	6:1	0.3 : 0.05	8.44 ± 0.09
**2**	8:1	0.4 : 0.05	10.4 ± 0.1
**3**	10:1	0.5 : 0.05	13.4 ± 0.2
**4**	12:1	0.6 : 0.05	16.4 ± 0.2
**5**	14:1	0.7 : 0.05	19.0 ± 0.2

5.9.4 Nucleophile **1e**

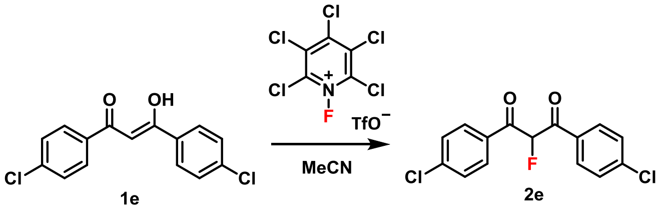

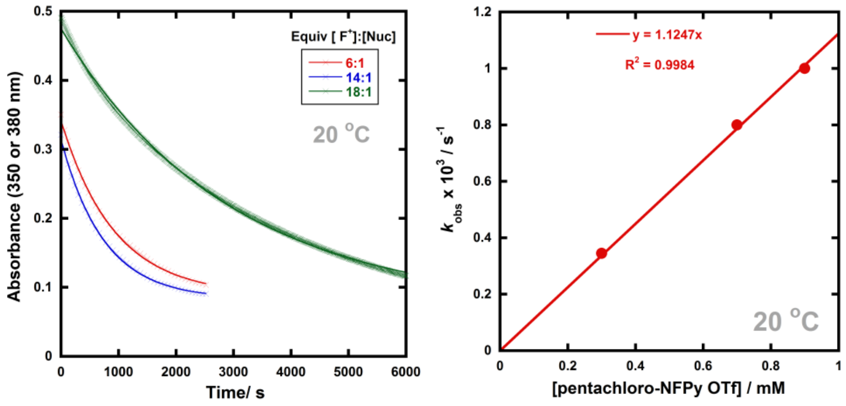

Table 59: k_{obs} values at different concentrations of 9 at 20 °C.

Experiment	Ratio of F^+ : Nuc	$[F^+]$: [Nuc]/ mM	$k_{obs} \times 10^3$ / s^{-1}
**1**	6:1	0.3 : 0.05	0.345 ± 0.002
**2**	14:1	0.7 : 0.05	0.801 ± 0.001
**3**	18:1	0.9 : 0.05	1.002 ± 0.001

6. Kinetics Studies Conducted by $^1H/^{19}F$ NMR

6.1 Fluorination of Nucleophile 1d by *N*-fluoropyridinium triflate (7a)

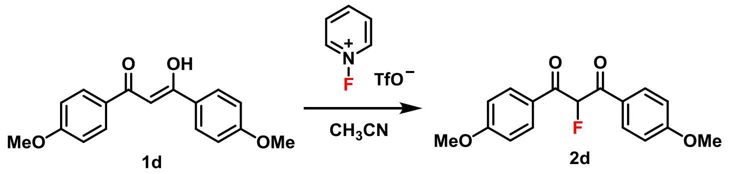

Enol **1d** (10 mg) and fluorinating reagent **7a** (86.9 mg) were dissolved in CH_3CN (0.65 mL) resulting in a 10-fold concentration difference between the reaction partners i.e. $[F^+]$:[Nuc] = 540 mM : 54 mM. The solution was transferred to an NMR tube containing a D_2O lock tube, and NMR spectra were acquired at ~24-hour intervals for 15 days. The NMR tube was kept in a 25 °C water bath when not acquiring NMR data. Using the MestreNova Data Analysis tool for stacked arrayed NMR data, the peak corresponding to the F atom in **2d** was integrated in each spectrum (**Figure 3**). The plot of relative peak area over time is also shown. This was fitted using the previously described method using KaleidaGraph software, and gave k_{obs} = 1.80 × 10^{-6} s^{-1}. The value for k_2 was calculated using $k_{obs} = k_2[F^+]$ to be 3.34 × 10^{-6} M^{-1} s^{-1}.

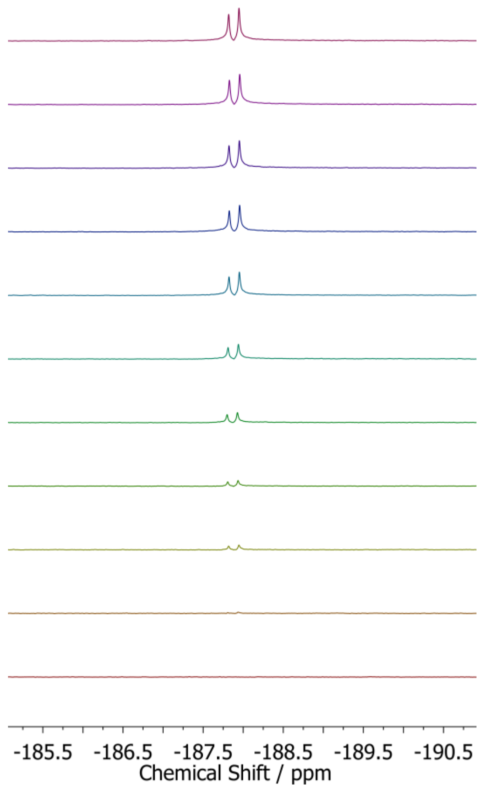

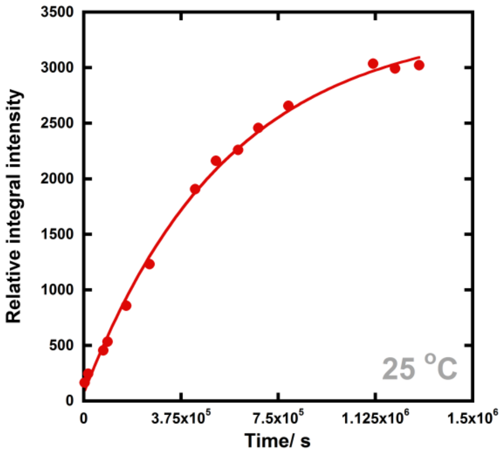

Figure 3: Fluorination of 1d-enol by 7a monitored by ^{19}F NMR.

The kinetics data from the NMR-monitored reaction were then compared to data from a UV/vis initial rates method, where the reaction was monitored for 5 days, at 25 °C. The k_{obs} values obtained were plotted against the concentration of fluorinating reagent **7a** to give $k_2 = 6.9 \times 10^{-6}\ M^{-1}\ s^{-1}$. The rate constants were within a factor of 2 of each, which is reasonable given the markedly different conditions and the many possibilities for error that could be introduced between the two experimental platforms.

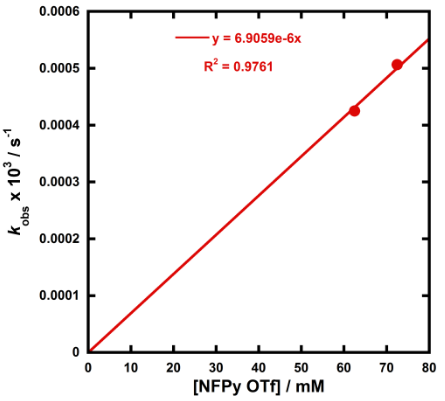

Figure 4: Plot of k_{obs} values versus concentration of 7a.

6.2 Fluorination of Nucleophile 1d by *N*-fluoropyridinium tetrafluoroborate (7b)

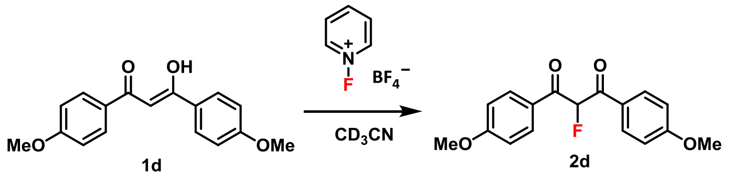

Enol **1d** and fluorinating reagent **7b** were dissolved in CD_3CN (concentrations shown in **Table 60**). ^{1}H and ^{19}F NMR spectra were acquired at ~24-hour intervals, for 9 days. The NMR tubes were kept in a 25 °C water bath when not acquiring NMR data. Using the MestreNova Data Analysis tool for stacked arrayed NMR data, the peak corresponding to *H*-2 in **2d** was integrated in each ^{1}H NMR spectrum (**Figure 5**). **Figure 6** shows a plot of relative integral intensity over time corresponding to each experiment. The k_{obs} values were fitted using the previously described method using KaleidaGraph software and gave $k_2 = 6.29 \times 10^{-6}\ M^{-1}\ s^{-1}$.

Table 60: Quantities used in NMR kinetics experiments.

Experiment	Equiv [7b]:[1d]	[7b]/ mM	[1d]/ mM	k_{obs} x 10^3/ s^{-1}
**1**	10:1	270	27	0.0018 ± 0.0003
**2**	15:1	406	27	0.0025 ± 0.0003

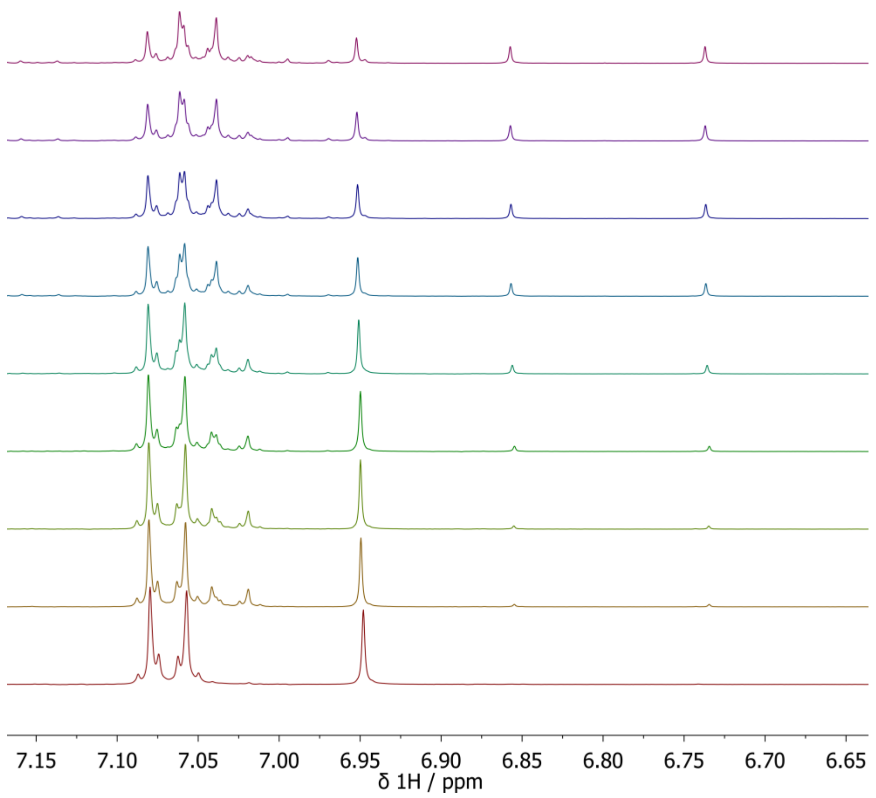

Figure 5: Fluorination of 1d-enol by 7b (Experiment 1), monitored discontinuously by ^{1}H NMR, with time intervals of ~1 day between each spectrum. The doublet at 6.80 ppm corresponds to *H*-2, which was integrated over time to determine the k_{obs} for the reaction. The singlet at 6.95 ppm and multiplet at 7.05-7.10 ppm correspond to 1d-enol.

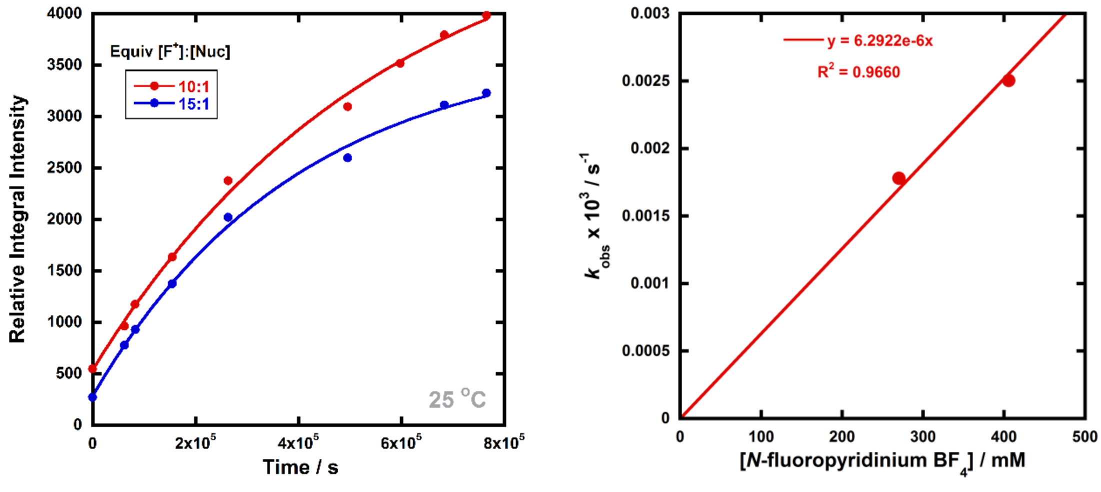

Figure 6: Fluorination of 1d-enol by 7b monitored by ^{1}H NMR.

6.3 Fluorination of Nucleophile 1d by 2,4,6-trimethyl-*N*-fluoropyridinium triflate (6a)

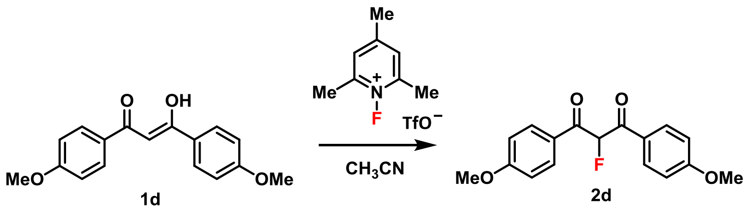

A similar method to that described in Section 6.1 was used to monitor kinetics of fluorination of enol **1d** by reagent **6a**, by ^{19}F NMR. The reaction was monitored for 50 days. Using the data obtained, it was determined that $k_2 = 1.34 \times 10^{-6}$.

Table 61: Quantities used in NMR kinetics experiments.

Experiment	Equiv [6a]:[1d]	[6a]/ mM	[1d]/ mM	k_{obs} x 10^3/ s^{-1}
**1**	10:1	439	44	0.00059 ± 0.00003
**2**	15:1	659	44	0.00088 ± 0.00004

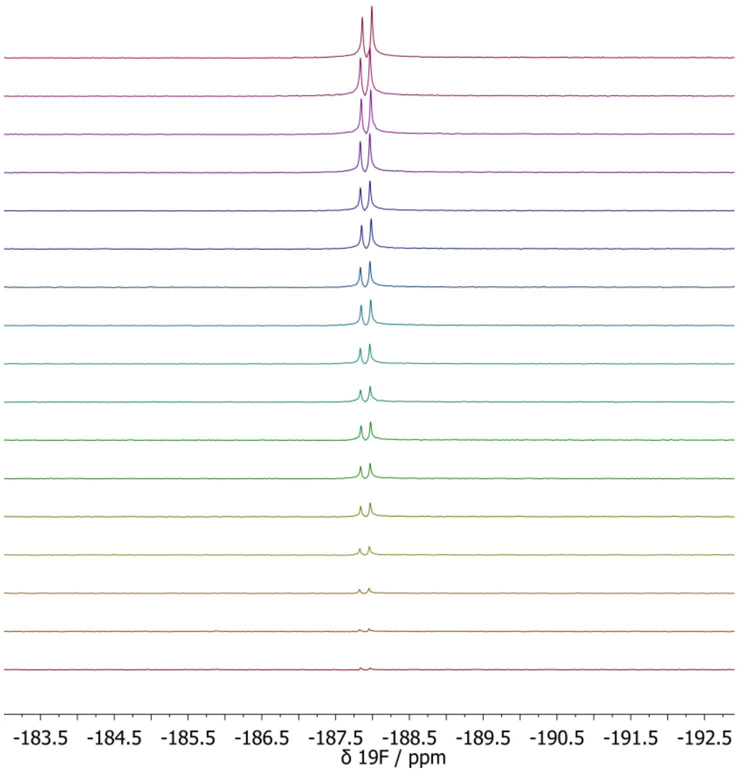

Figure 7: Fluorination of 1d-enol by 6a (Experiment 1), monitored discontinuously by ^{19}F NMR, with time intervals of ~1 day between each spectrum.

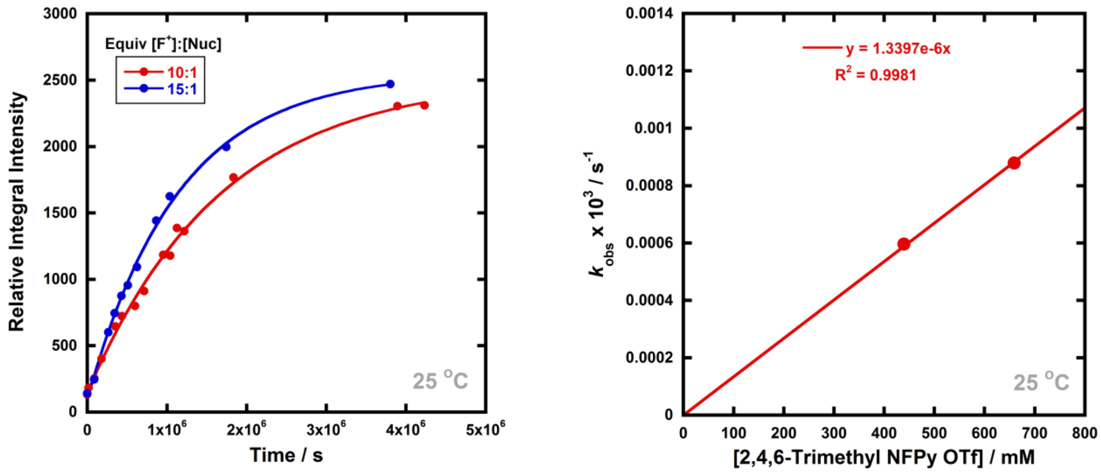

Figure 8: Fluorination of 1d by 6a monitored by ^{19}F NMR.

6.4 Fluorination of Nucleophile 1d by 2,4,6-trimethyl-*N*-fluoropyridinium BF_4^- (6b)

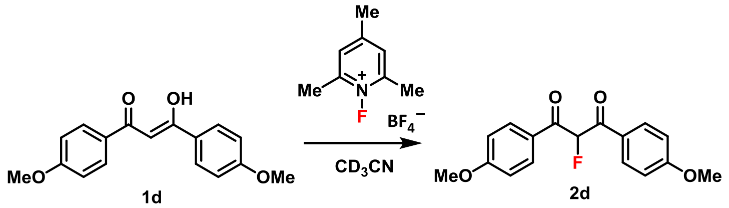

A similar method to that described in Section 6.2 was used to monitor kinetics of fluorination of enol **1d** by reagent **6b**, by ^{1}H NMR. Spectra were acquired at ~24 h intervals, for 50 days. Quantities used are shown in **Table 62**. Using the data obtained, it was determined that $k_2 = 2.63 \times 10^{-6}$.

Table 62: Quantities used in NMR kinetics experiments.

Experiment	Equiv [6b]:[1d]	[6b]/ mM	[1d]/ mM	k_{obs} x 10^3/ s^{-1}
**1**	12.75:1	280	22	0.00092 ± 0.00004
**2**	19:1	420	22	0.0011 ± 0.0002

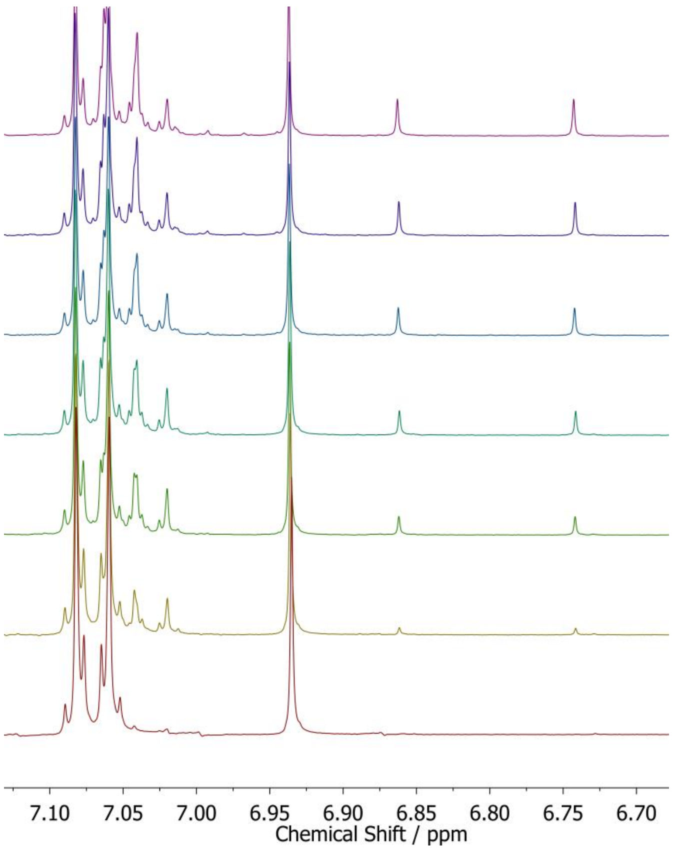

Figure 9: Fluorination of 1d by 6b monitored by ^{1}H NMR. Doublet at 6.80 ppm corresponds to 2d, which was integrated over time.

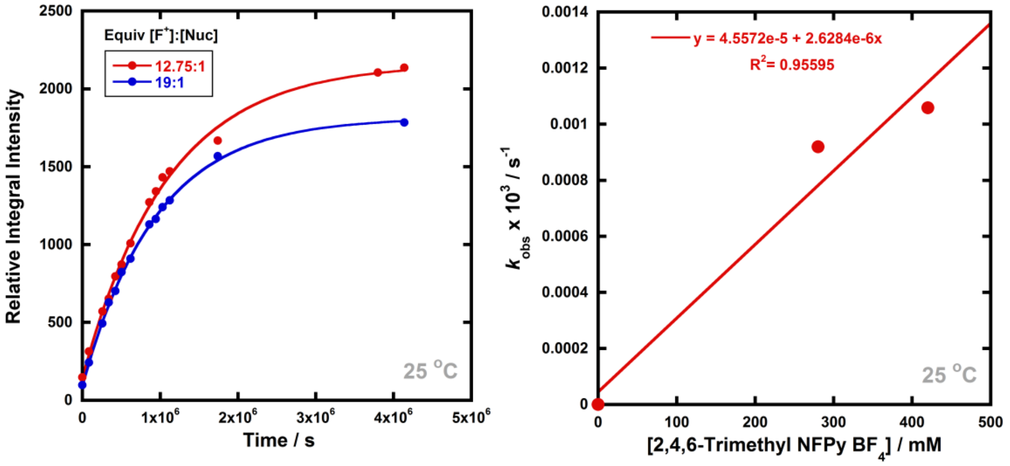

Figure 10: Fluorination of 1d by 6b monitored by ^{1}H NMR.

7. Reactions Monitored by LCMS

7.1 Distinguishing Keto and Enol Tautomers

Viewing the chromatograms at the λ_{max} values of the keto and enol forms (250 nm and 341 nm, respectively) allowed us to distinguish the identities of the peaks. The peaks corresponding to enol and keto forms of **1a** (chromatogram shown below) are labelled. As discussed in the main text, the enol tautomer absorbs at both 250 nm and 341 nm, with different extinction coefficients at each wavelength, as seen below.

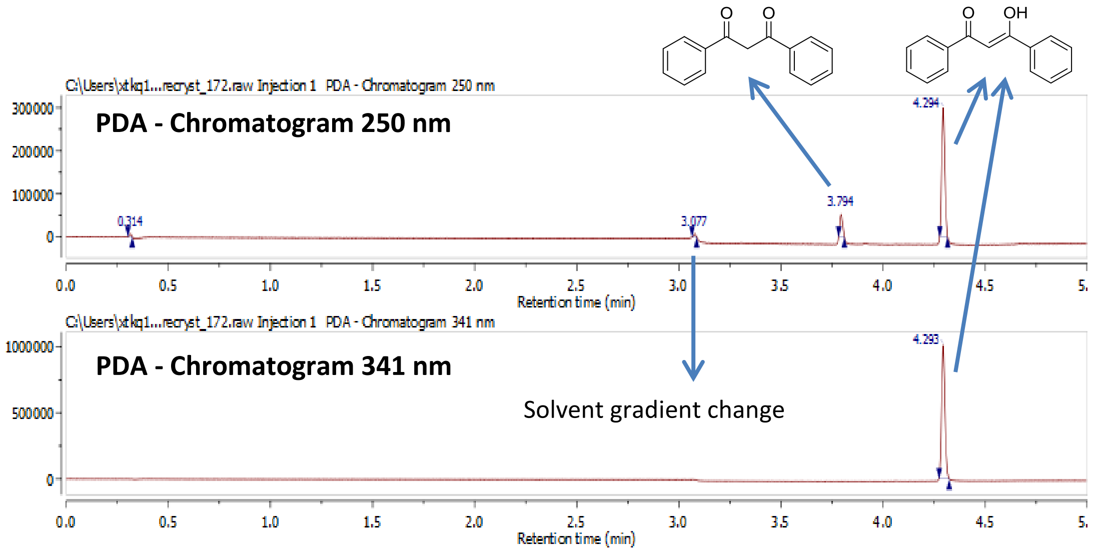

Figure 11: LC-MS trace for 1a showing separate peaks for keto and enol forms in the chromatogram, verified by viewing the chromatogram at different wavelengths.

7.2 Fluorination of 1a by Selectfluor™

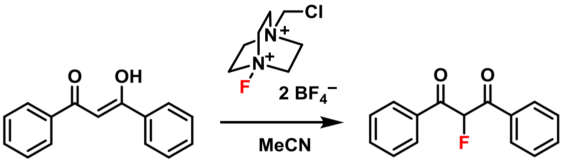

1a 2a

This reaction was conducted under pseudo-first order conditions with 10-fold excess of Selectfluor™. [Selectfluor™] = 0.5 mM; [**1a**] = 0.05 mM. LCMS spectra were acquired after 5 and 24 hours. Peak integrals of enol and keto starting materials are in the ratio of 4:1 at both time intervals; hence the ratios of both tautomers remain constant throughout the reaction.

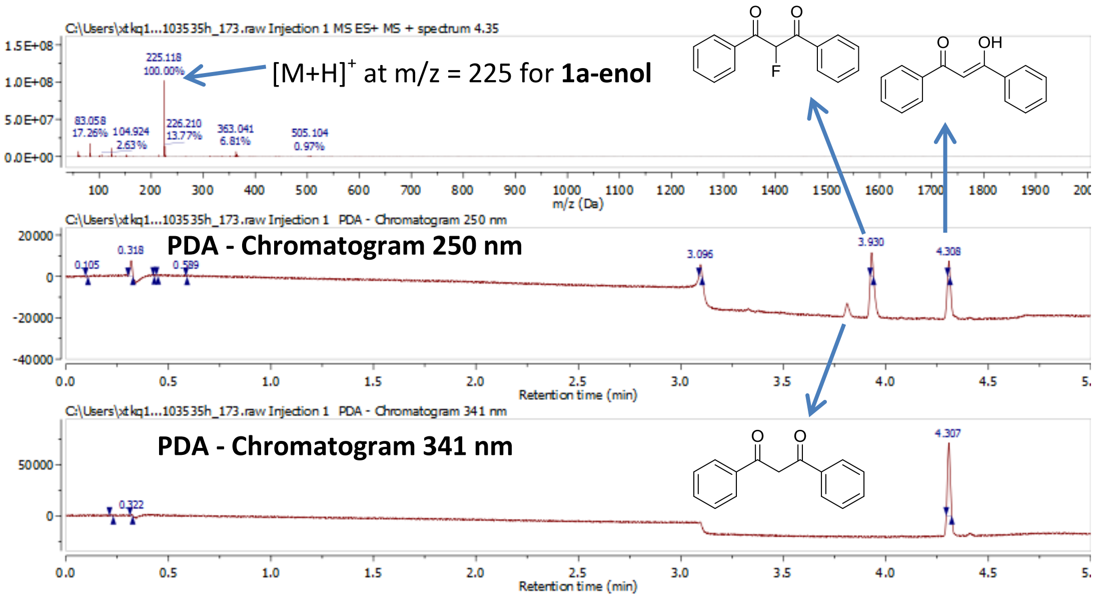

Figure 12: LCMS of reaction mixture after 5 hours.

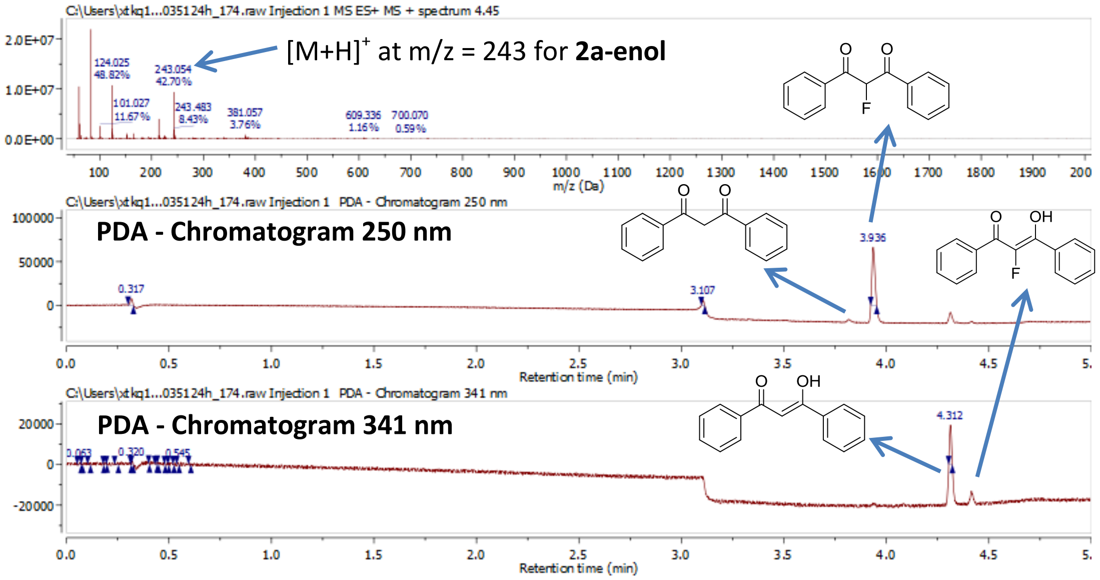

Figure 13: LCMS of reaction mixture after 24 hours.

7.3 Fluorination of 1d by 2,6-dichloro-NFPy tetrafluoroborate (8b)

The reaction below for fluorination of **1d** by **8b** was monitored by LCMS analysis, which was carried out under bimolecular conditions, where [**1d**] = [**8b**] = 3 mM. The appearance of product **2d** over time is shown in **Figure 3c** of the main text.

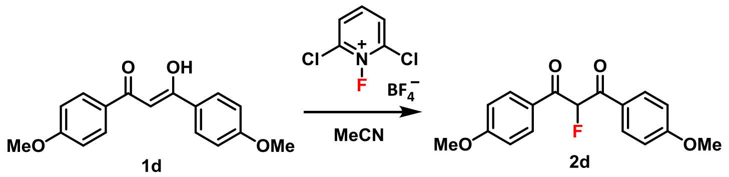

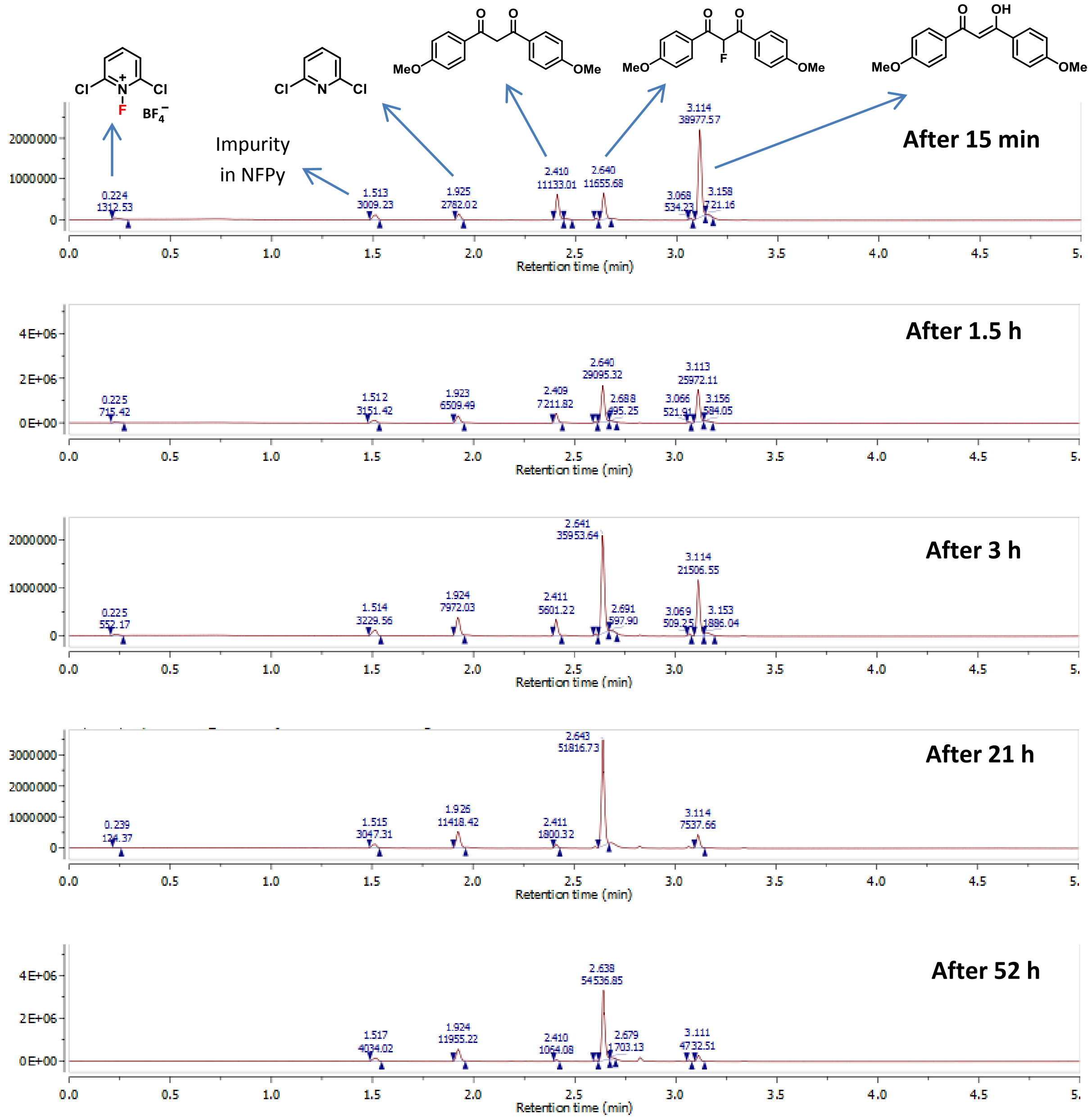

8. Kinetics of Fluorination of 2a

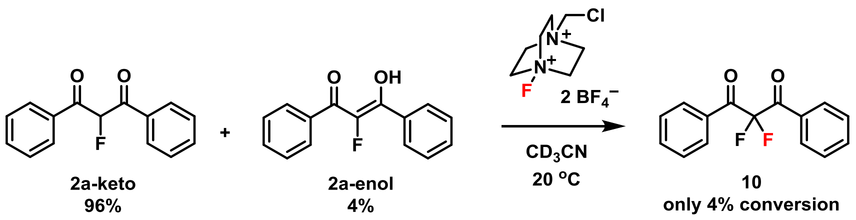

A ^{19}F NMR spectrum of recrystallized **2a** was acquired in CD_3CN (**Figure 14 Spectrum 1**), which shows that the keto-enol tautomers are present in a 96:4 ratio.

Selectfluor™ (7 mg) was added to a solution of compound **2a** (5 mg) in CD_3CN (0.7 mL), where [**2a**] = [Selectfluor™] = 29.5 mM. The ^{19}F NMR spectrum acquired after 20 min (**Figure 14 Spectrum 2**) showed that the peak at −170 ppm corresponding to **2a-enol** had disappeared. A new peak at −103 ppm appeared, corresponding to the difluoro product **10**. The reaction mixture was monitored by ^{19}F NMR for a further 4 days (**Figure 15**) at 20 °C, and showed no change in peak intensity of **2a-keto** (**Figure 16**). Thus the NMR spectra confirm that the tautomerism of **2a-keto** to **2a-enol** does not occur over this timescale.

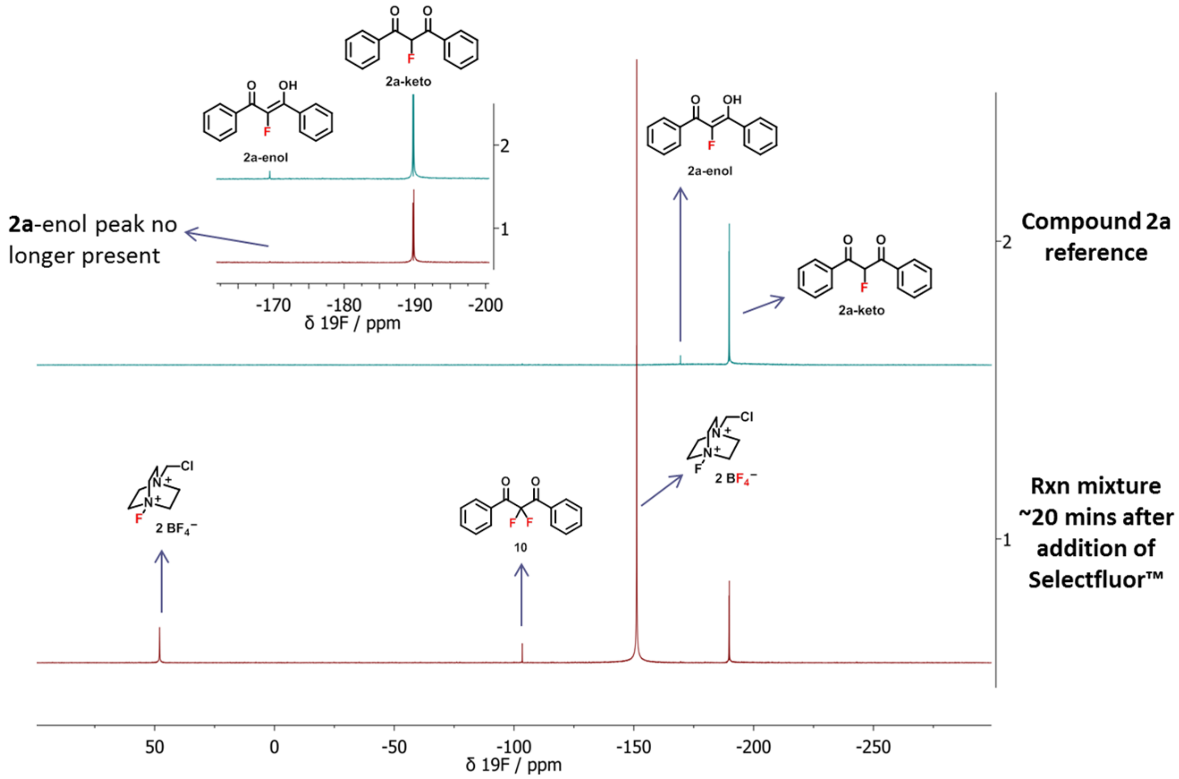

Figure 14: Spectrum 1) ^{19}F NMR spectrum for the addition of Selectfluor™ to compound 2a. Spectrum 2) ^{19}F NMR spectrum for keto-enol mixture 2a (96% keto form in CD_3CN).

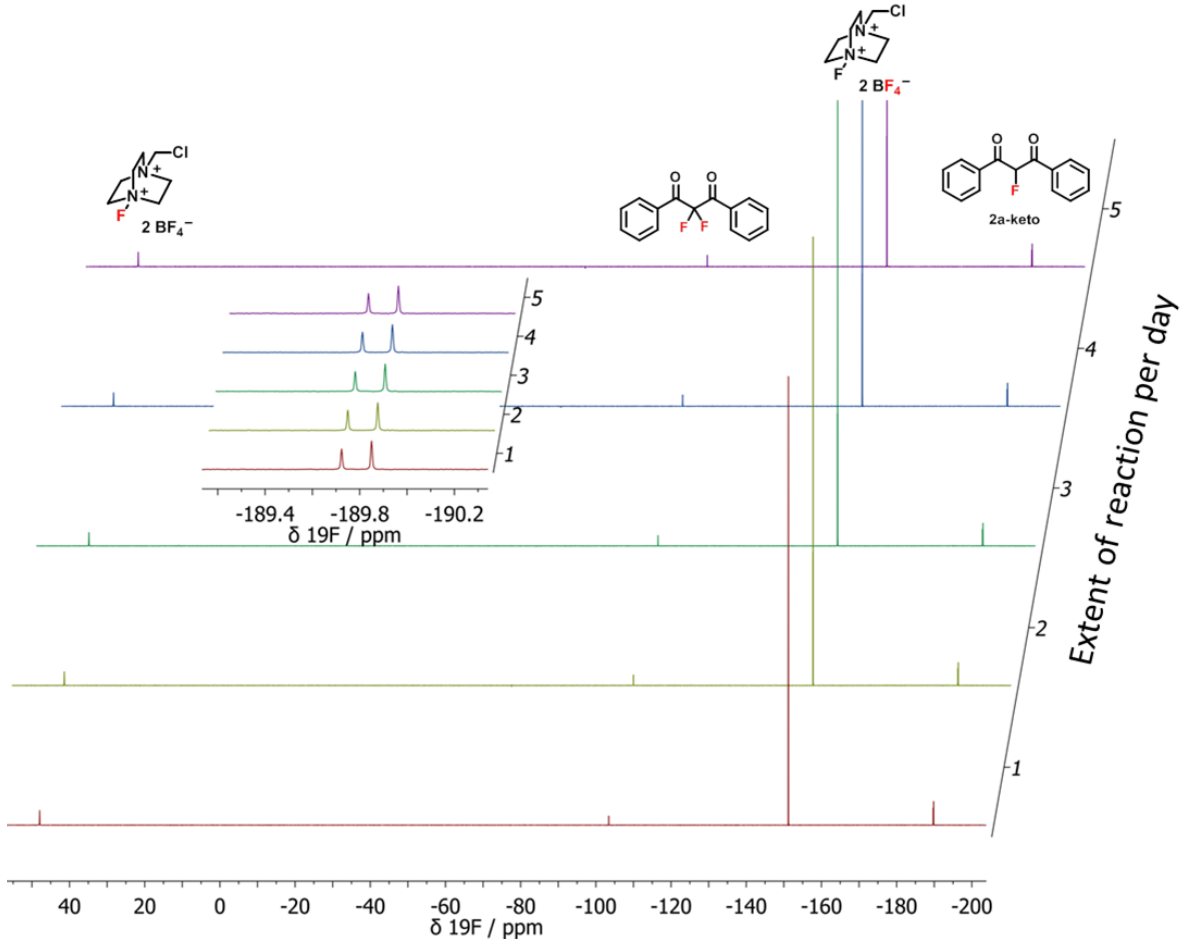

Figure 15: Reaction mixture monitored over 5 days, showing no change in the concentration of 2a-keto.

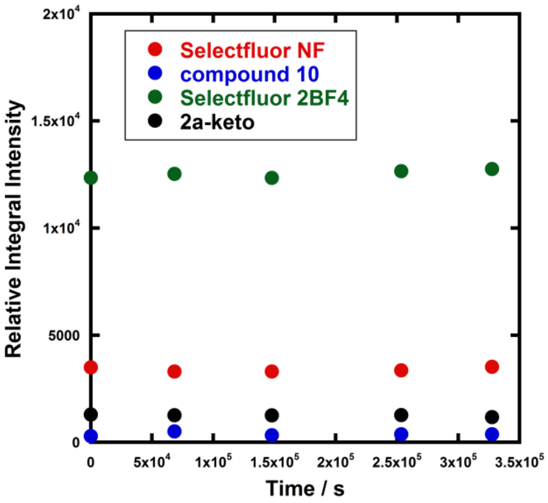

Figure 16: Graph of relative integral intensities over time, showing no change in peak intensity for 2a-keto (black data points), hence the fluorination reaction does not proceed via the fluoroketo form on this timescale at this concentration, at 20 °C.

9. References

1. N. Y. Yang, Z. L. Li, L. Ye, B. Tan and X. Y. Liu, *Chem. Comm.*, 2016, **52**, 9052-9055.
2. W. M. Nau, H. M. Harrer and W. Adam, *J. Am. Chem. Soc.*, 1994, **116**, 10972-10982.
3. H. Kaneyuki, *B. Chem. Soc. Jpn.*, 1962, **35**, 523-525.
4. J. Zawadiak and M. Mrzyczek, *Spectrochim. Acta A*, 2012, **96**, 815-819.
5. N. M. Shavaleev, R. Scopelliti, F. Gumy and J.-C. G. Bünzli, *Eur. J. Inorg. Chem.*, 2008, **9**, 1523-1529.
6. T. Kitamura, S. Kuriki, M. H. Morshed and Y. Hori, *Org. Lett.*, 2011, **13**, 2392-2394.
7. A. S. Reddy and K. K. Laali, *Tetrahedron Lett.*, 2015, **56**, 5495-5499.
8. B. Košmrlj and B. Šket, *Org. Lett.*, 2007, **9**, 3993-3996.
9. A. D. Becke, *J. Chem. Phys.*, 1993, **98**, 5648-5652.
10. C. T. Lee, W. T. Yang and R. G. Parr, *Phys. Rev. B*, 1988, **37**, 785-789.
11. G. A. Petersson, A. Bennett, T. G. Tensfeldt, M. A. Allaham, W. A. Shirley and J. Mantzaris, *J. Chem. Phys.*, 1988, **89**, 2193-2218.
12. G. A. Petersson and M. A. Allaham, *J. Chem. Phys.*, 1991, **94**, 6081-6090.
13. GAUSSIAN09, Revision A.02, M. J. Frisch et al, Gaussian, Inc., Wallingford CT, 2009.
14. J. Tomasi, B. Mennucci and E. Cances, *J. Mol. Struc. Theochem*, 1999, **464**, 211-226.
15. T. G. Cooper, K. E. Hejczyk, W. Jones and G. M. Day, *J. Chem. Theory Comput.*, 2008, **4**, 1795-1805.
16. A. J. Cruz-Cabeza and C. R. Groom, *CrystEngComm*, 2011, **13**, 93-98.
17. O. V. Dolomanov, L. J. Bourhis, R. J. Gildea, J. A. K. Howard and H. Puschmann, *J. Appl. Crystallogr.*, 2009, **42**, 339-341.
18. G. M. Sheldrick, *Acta Crystallogr., Sect. A*, 2008, **64**, 112-122.
19. N. Dege, I. Yildirim, A. Guldeste, H. Inac, I. Koca, N. Kahveci, A. Ozyetis and O. Buyukgungor, *Acta Crystallogr., Sect. E: Struct. Rep. Online*, 2005, **61**, O60-O62.
20. K. Sato, G. Sandford, K. Shimizu, S. Akiyama, M. J. Lancashire, D. S. Yufit, A. Tarui, M. Omote, I. Kumadaki, S. Harusawa and A. Ando, *Tetrahedron*, 2016, **72**, 1690-1698.
